# OMA1 protects from liver injury and tumorigenesis during aging by controlling hepatic immunogenicity

**DOI:** 10.1038/s44318-026-00839-4

**Published:** 2026-06-23

**Authors:** Yolanda Martí-Mateos, María del Mar Muñoz-Hernández, Manuel M Gómez de las Heras, José Ignacio Escrig-Larena, José Luis Cabrera-Alarcón, Rebeca Acín-Pérez, Enrique Calvo, Sara Natalia Jaroszewicz, Lucía de Prado-Rivas, Eva Raquel Martínez-Jiménez, Macarena De Andrés-Laguillo, Esther García-Domínguez, María Carmen Gómez-Cabrera, José Viña, Rui Benedito, Alejo Efeyan, Jesús Vázquez, María Mittelbrunn, María Concepción Jiménez-Gómez, José Antonio Enríquez

**Affiliations:** 1 Centro Nacional de Investigaciones Cardiovasculares Carlos III (CNIC), Madrid, Spain; 2 Centro de Biología Molecular Severo Ochoa (CBMSO), Consejo Superior de Investigaciones Científicas and Universidad Autónoma de Madrid (CSIC-UAM), Madrid, Spain; 3 Centro de Investigación Biomédica en Red Fragilidad y Envejecimiento Saludable. Instituto de salud Carlos III (CIBERFES), Madrid, Spain; 4 Centro de Investigación Biomédica en Red de Enfermedades Cardiovasculares. Instituto de salud Carlos III (CIBERCV), Madrid, Spain; 5 Centro Nacional de Investigaciones Oncológicas (CNIO), Madrid, Spain; 6 Freshage Research Group, Department of Physiology, Faculty of Medicine, University of Valencia, Fundación Investigación Hospital Clínico Universitario/INCLIVA, Valencia, Spain

**Keywords:** Cancer, Immunology, Metabolism

## Abstract

Hepatic inflammation and immunosurveillance play major roles in the progression of liver cancer. A common trigger for hepatic inflammation is oxidative stress, which stems from mitochondrial dysfunction. Here, we demonstrate that deletion of the mitochondrial stress integrator OMA1 increases hepatic primary tumor incidence and impairs survival in mice. Persistent activation of the KEAP1-Nrf2 oxidative stress pathway in the absence of OMA1 promotes early liver injury, which progresses into chronic hepatic inflammation and fibrosis during aging. Exhausted CD8^+^ and CD4^+^ T cells gradually accumulate in *Oma1*^KO^ livers, facilitating hepatic tumor progression. Adoptive transfer and bone marrow-transplant experiments indicate that hepatic immunogenicity increases in the absence of parenchymal OMA1. Furthermore, hepatocyte-specific *Oma1* deletion is sufficient to trigger NRF2 signaling, hepatocyte death, and immune exhaustion, suggesting that immunosurveillance during liver aging relies on the hepatic expression of OMA1. Given the therapeutic interest of OMA1 in several pathologies, these data are crucial to guide the generation of safe OMA1-targeted therapies.

## Synopsis

Modelling chronic liver disease in vivo remains challenging, and its molecular drivers prematurely explored. This study uncovers a liver-specific role for mitochondrial metalloprotease OMA1 in regulating hepatocyte metabolism and immunogenicity, preventing chronic inflammation, immune exhaustion, and tumorigenesis.

- OMA1 depletion in mice decreases longevity and accelerates hepatic tumorigenesis.
- Young *Oma1*^KO^ mice show precocious liver dysfunction and fibrosis, aberrant NRF2-mediated oxidative stress signaling, and deficient mitochondrial respiration.
- Hepatocyte-specific *Oma1* deletion triggers progressive immune exhaustion and increased immunogenicity in genetic background-specific manner.

## Abstract (web summary)

Hepatic mitochondrial metalloprotease OMA1 safeguards liver health by suppressing oxidative stress response and enabling immunosurveillance.

## Graphical abstract

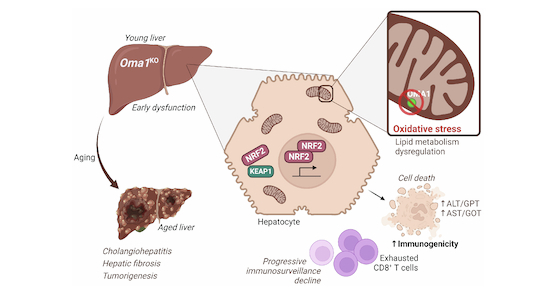

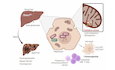

## Introduction

Cancer cells adapt their metabolism to their increased need for biosynthesis and antioxidants defences to allow proliferation, tumor growth, and metastasis. Nonetheless, there is considerable debate regarding whether this metabolic shift influences how immune cells identify and remove cancerous cells. Initial evidence emerged highlighting the significance of mitochondrial electron flow in determining the immune response, particularly in melanoma (Mangalhara et al, 2023). Given this context, it prompted us to inquire whether mitochondrial stress might influence cells’ immunogenicity.

The mitochondrial metalloprotease OMA1 is a central integrator of stress in the cell (Gilkerson et al, 2021). OMA1 is localized in the inner mitochondrial membrane and, upon stress activation, it proteolyzes several targets (i.e., OPA1, DELE1, DNAJC15, AIFM1). OMA1-mediated OPA1 proteolysis leads to mitochondrial fragmentation (Ehses et al, 2009; Head et al, 2009; Baker et al, 2014) and apoptosis (Frezza et al, 2006; Jiang et al, 2014), whereas DELE1 proteolysis activates the integrated stress response through HRI-mediated eIF2α phosphorylation (Guo et al, 2020; Fessler et al, 2020). OMA1 deletion has been regarded as protective in various pathological contexts, including heart failure (Acin-Perez et al, 2018; Hu et al, 2020), neurodegeneration (Korwitz et al, 2016), and ischemic kidney injury (Varanita et al, 2015). Whole-body ablation of *Oma1* in mice is not deleterious and allows normal embryonic development: animals reach a healthy adulthood and show normal fertility (Quiros et al, 2012). Therefore, OMA1 has been proposed as a potentially powerful therapeutic target (Alavi, 2021, 2022).

However, growing evidence indicates that *OMA1* deletion could also be detrimental in certain physiological scenarios, as this protease actually presents pro-survival functions relevant for enduring stress conditions. In the context of neurodegeneration, it has been shown that OMA1 presence delays spinal cord axon degeneration from *Yme1l*-deficient neurons (Sprenger et al, 2019). Similarly, OMA1 activity delayed ferroptosis-mediated cardiomyopathy in *Cox10*-deficient mice (Ahola et al, 2022), suggesting that OMA1 protein has an important homeostatic role under stress conditions. Since the liver is a central stress integrator in the body and ferroptosis is a known driving cause for liver disease (Chen et al, 2022), we wondered whether OMA1 depletion might mediate liver injury sensitivity during aging.

Chronic liver disease (CLD) refers to a broad array of conditions characterized by progressively decreased hepatic function, inflammation, and fibrosis, which can eventually lead to tumorigenesis (Sharma and Nagalli, 2023). When the liver fails, organismal life is compromised, as accounted for the CLD high mortality and morbidity worldwide (Huang et al, 2023). In 2019, CLD was associated with 2.4% of global deaths, ~60% among men and 40% among women (World Health Organisation, 2023). The most common causes for CLD are alcohol abuse, metabolic dysfunction-associated steatotic liver disease (MASLD), and viral hepatitis (Sharma and Nagalli, 2023).

Hepatic inflammation plays a major role in the progression of CLD and the development of hepatocellular carcinoma (HCC) (Del Campo et al, 2018). Under contexts of organ stress (e.g., infection, steatosis, or oxidative stress), molecules released by the liver may act as pathogen- or damage-associated molecular patterns (PAMPs or DAMPs, respectively), triggering inflammation (Del Campo et al, 2018). Chronic stress drives persistent immune activation and T cell exhaustion, contributing to the formation of tumors (Jiang et al, 2015).

T-cell exhaustion refers to a dysfunctional state of effector T cells defined by a reduced capacity to secrete cytokines and an enhanced expression of inhibitory receptors (such as PD-1 or Tim-3) (Jiang et al, 2015). T-cell exhaustion is thought to develop as a response to chronic antigen stimulation, aimed at limiting immunopathology and autoreactivity. With aging, immune exhaustion and senescence are enhanced (Carrasco et al, 2022), promoting chronic, nonresolving inflammation. CLD patients present increased expression of PD-1 (Lebossé et al, 2019; Ma et al, 2019; Carrasco et al, 2022), which further contributes to immunosuppression (Lebossé et al, 2019) and increased tumorigenesis (Jiang et al, 2015).

One of the main triggers for the initiation of hepatic inflammation is oxidative stress (Cichoz-Lach and Michalak, 2014). Under oxidative stress conditions, the KEAP1-Nrf2 signaling pathway gets activated, promoting antioxidant and detoxifying responses (Zhou et al, 2022). NRF2, as well as its transcriptional target NQO1 (Cheng et al, 2015), accumulates under CLD, suggesting that the KEAP1-Nrf2 pathway is activated in CLD. Given that mitochondria are the primary site for reactive oxygen species (ROS) generation in the cell (Hernansanz-Agustín and Enríquez, 2021), there is a manifest link between mitochondrial dysfunction and liver injury initiation (Scharping et al, 2021).

Analyzing aging in whole-body *Oma1*^WT^ and *Oma1*^KO^ mice, we found that *Oma1*^KO^ mice present increased mortality and hepatic primary tumors incidence. Prior to tumor development, young *Oma1*^KO^ mice already show early liver damage and increased NRF2-dependent oxidative stress, leading to increased cell death in the liver. With time, *Oma1*^KO^ CD8^+^ T cells become progressively exhausted, and the early liver damage observed in *Oma1*^KO^ progresses into chronic liver inflammation and hepatic fibrosis. The dysfunction seen in *Oma1*^KO^ animals originates in hepatocytes, which are sufficient to promote NRF2 signaling, hepatocyte death, and T-cell exhaustion, indicating that hepatic expression of OMA1 protects against CLD and tumorigenesis by controlling liver immunogenicity during aging.

## Results

**Figure 1:**
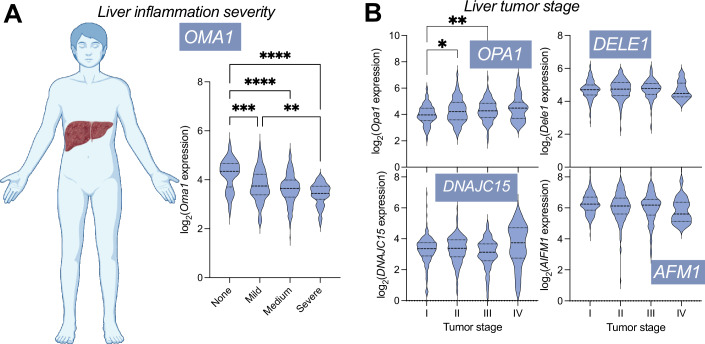
Impact of OMA1 expression and its proteolytic targets OPA1 and DELE1 in liver tumor stage. (**A**) Human data on the correlation of *OMA1* expression to liver inflammation severity. None vs. Mild *P* = 0.0004, None vs. Medium *P *< 0.0001, None vs. Severe *P* < 0.0001, Medium vs. Severe *P* = 0.0026. Sample size: None (*n* = 48), Mild (*n* = 148), Medium (*n* = 231), Severe (*n* = 47). (**B**) Human data on the correlation of *OPA1*, *DELE1, DNAJC15*, and *AIFM1* expression to liver tumor stage. OPA1: I vs. II *P* = 0.0295, I vs. III *P* = 0.0023. Sample size: I (*n* = 221), II (*n* = 148), III (*n* = 129), IV (*n* = 21). Data obtained from GepLiver and analyzed by one-way ANOVA and nonparametric Kuskal–Wallis test and Dunn’s multiple comparisons test *P* > 0.05, ^∗^*P* < 0.05, ^∗∗^*P* < 0.01, ^∗∗∗^*P* < 0.001, and ^∗∗∗∗^*P* < 0.0001. Source data are available online for this figure.

We first analyzed the relationship between OMA1 and liver disease using publicly available human data (GepLiver, (Li et al, 2023)), finding a correlation between the expression of *OMA1* and the severity of liver inflammation (Li et al, 2023) (Fig. 1A). Since the known OMA1 proteolytic targets are OPA1, DELE1, DNAJC15 and AIFM1 we next inquired if the expression of any of them correlated with human liver tumor stage. Interestingly, *OPA1* expression revealed a positive correlation with tumor severity (Fig. 1B), in agreement with the fact that OPA1 is an inactivating proteolytic target of OMA1 (in terms of mitochondrial dynamics, reduced *OPA1* expression mimics the effects of excessive OMA1 activity, while elevated *OPA1* expression phenocopies OMA1 inactivation).

On the other hand, no correlation between *DELE1*, *DNAJC15*, or *AIFM1* levels and liver tumor stage was observed (Fig. 1B). Thus, we concluded that the OMA1-OPA1 axis is likely playing a role in hepatic tumor progression in humans and decided to explore this link further using genetic mouse models.

### Increased mortality and hepatic primary tumor incidence in *Oma1*^KO^ mice

**Figure 2:**
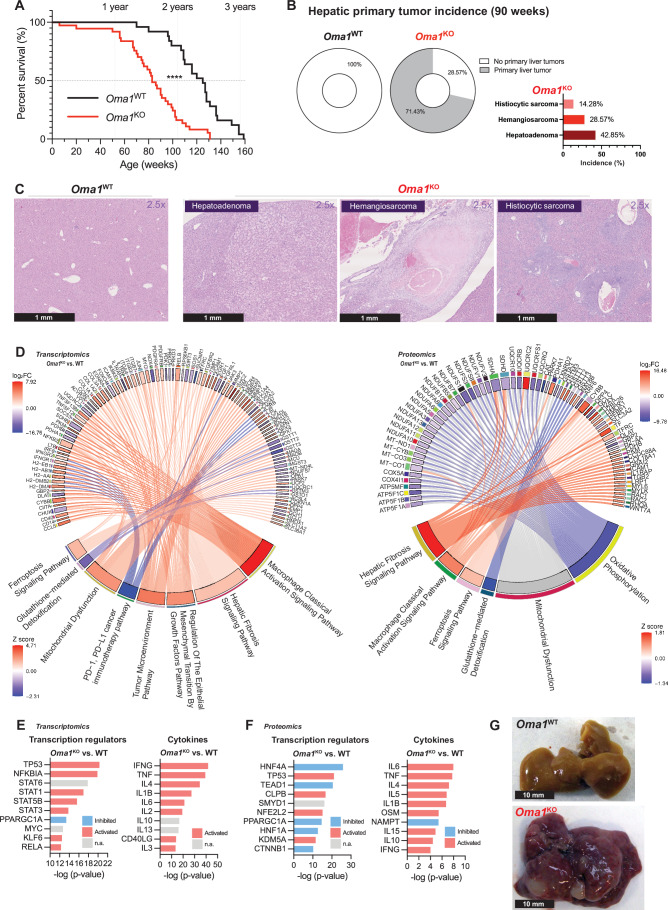
Decreased survival and increased hepatic primary tumor incidence in *Oma1*^KO^ mice. (**A**) Survival analysis of male *Oma1*^WT^ (*n* = 25) and *Oma1*^KO^ (*n* = 37) mice in an inbred C57BL6/JOlaHsd background. This graph contains data from two separate experiments. *P* value from Log-rank (Mantel–Cox) test is represented in the graph. *P* < 0.0001. (**B**) Hepatic primary tumor incidence in a cohort of male *Oma1*^WT^ (*n* = 5) and *Oma1*^KO^ (*n* = 7) mice euthanized at 90 weeks of age. Hepatic primary tumor type incidence found in these 90-week-old *Oma1*^KO^ mice is depicted in a bar graph below. (**C**) Representative H&E histological analyses of 90-week-old liver paraffin sections showing the diverse hepatic primary tumor types found in male *Oma1*^KO^ mice compared to *Oma1*^WT^ animals. (**D**) Chord diagrams depicting significantly up- or downregulated pathways of interest in 90-week-old male *Oma1*^KO^ mice compared to *Oma1*^WT^ animals according to Ingenuity Pathways Analysis (IPA) using transcriptomics (left) or proteomics (right) data. (**E**) IPA-obtained upstream regulators in 90-week-old male *Oma1*^KO^ mice compared to *Oma1*^WT^ animals filtered by transcription factors or cytokines from transcriptomics data. Benjamini–Hochberg method, adjusted *P* value < 0.05. (**F**) IPA-obtained upstream regulators in 90-week-old male *Oma1*^KO^ mice compared to *Oma1*^WT^ animals filtered by transcription factors or cytokines from proteomics data. Benjamini–Hochberg method, adjusted *P* value < 0.05. (**G**) Representative digital photographs from a euthanized 50-week-old male *Oma1*^WT^ mouse and an autopsy from an *Oma1*^KO^ mouse presenting liver tumors at 65 weeks of age. Color in images has been adjusted to match their divergences in lightning, so it is not informative. ^∗^*P* < 0.05, ^∗∗^*P* < 0.01, ^∗∗∗^*P* < 0.001, and ∗∗∗∗*P* < 0.0001*.* Source data are available online for this figure.

**Figure EV1:**
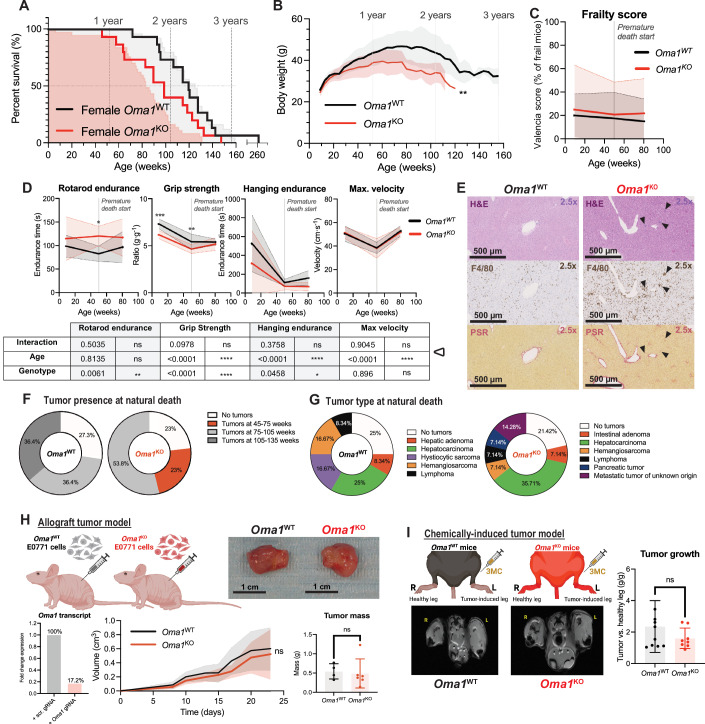
Decreased survival and increased hepatic primary tumor incidence in *Oma1*^KO^ mice. (**A**) Survival analysis of female *Oma1*^WT^ (*n* = 15) and *Oma1*^KO^ (*n* = 15) mice in an inbred C57BL6/JOlaHsd background. (**B**) Body weight evolution (*P* = 0.0012) of male *Oma1*^WT^ (*n* = 25) and *Oma1*^KO^ (*n* = 37) mice in an inbred C57BL6/JOlaHsd background. This graph contains data from two separate experiments. *P* value from a Mixed-effects model testing the “OMA1 expression” fixed effect is represented in the graph. (**C**) Frailty score from male *Oma1*^WT^ and *Oma1*^KO^ mice at 10, 50, and 80 weeks of age, including rotarod endurance, grip strength, hanging endurance, and maximum velocity. Mice cohorts at the analyzed time points were not the same individuals. Graphical representation using continuous lines has been chosen for visual simplicity. The number of animals used at these 10, 50, and 80 weeks of age cohorts were *Oma1*^WT^: *n* = 10, *n* = 10, and *n* = 5, respectively; and *Oma1*^KO^: *n* = 10, *n* = 12, and *n* = 8, respectively. (**D**) Performance evolution at individual functional tests using male *Oma1*^WT^ and *Oma1*^KO^ mice at 10, 50, and 80 weeks of age, including rotarod endurance (50w *P* = 0.0187), grip strength (9w *P* = 0.0056, 50w *P* < 0.0001), hanging endurance, and maximum velocity. Mice cohorts at the analyzed time points were not the same individuals. Graphical representation using continuous lines has been chosen for visual simplicity. The number of animals used at these 10, 50, and 80 weeks of age cohorts were *Oma1*^WT^: *n* = 10, *n* = 10, and *n* = 5, respectively; and *Oma1*^KO^: *n* = 10, *n* = 12, and *n* = 8, respectively. *P* values from two-way ANOVA tests assessing “Age”, “Genotype”, and “Interaction” as possible sources of variation are indicated below. (**E**) Representative H&E, F4/80, and PSR histological analysis from a male *Oma1*^KO^ mouse autopsy presenting liver damage at 58 weeks of age compared to a representative 60-week-old *Oma1*^WT^ liver. Arrows indicate multifocal liver microgranuloma lesions. (**F**) Percentage of analyzed *Oma1*^WT^ (*n* = 11) and *Oma1*^KO^ (*n* = 13) carcasses presenting tumors in survival experiments subdivided by their age at demise. (**G**) Tumor type percentages in analyzed *Oma1*^WT^ (*n* = 11) and *Oma1*^KO^ (*n *= 13) carcasses presenting tumors in survival experiments. (**H**) Allograft tumor model, depicting: the experimental design, consisting of the injection of *Oma1*^WT^ and *Oma1*^KO^ E0771 lines generated by CRISPR-Cas9 technology into *nude* mice (above, left); tumor volume growth estimation of *Oma1*^WT^ and *Oma1*^KO^ allografts (mean ± SD) (below, left); representative images of *Oma1*^WT^ and *Oma1*^KO^ allografts at sacrifice (above, right); and final tumor dimensions (volume and mass) of *Oma1*^WT^ and *Oma1*^KO^ allografts at sacrifice (below, right). “Tumor volume” and “Tumor mass” values did not pass normality. *P* values from Mann–Whitney tests are represented in each graph. (**I**) Chemically induced tumor model, depicting: the experimental design, consisting of the intramuscular injection of 3MC in male constitutive *Oma1*^WT^ and *Oma1*^KO^ mice (above, left); representative MRI images from *Oma1*^WT^ (*n* = 10) and *Oma1*^KO^ (*n* = 9) mice after 3 months of 3MC-induction (R = right, L = left) (below, left); and final tumor-induced to healthy leg mass from *Oma1*^WT^ and *Oma1*^KO^ mice after 3 months of 3MC-induction (mean ± SD) (right). “Tumor growth” values passed normality. *P* value from an unpaired two-tailed Student’s *t* test is represented in the graph. ns *P* > 0.05, ^∗^*P* < 0.05, ^∗∗^*P* < 0.01, ^∗∗∗^*P* < 0.001, and ^∗∗∗∗^*P* < 0.0001.

To determine if the constitutive deletion of *Oma1* had any physiological effects on liver aging in mice, we performed survival experiments. Interestingly, *Oma1*^KO^ mice longevity was significantly reduced (Fig. 2A). Median survival in male *Oma1*^KO^ mice was reduced by 33.6% (from 125 to 83 weeks), and their maximum survival dropped by 25.5% (from 149 to 111 weeks). Conversely, median survival in female *Oma1*^KO^ mice was only reduced an 18% (from 120 to 99 weeks) (Fig. EV1A), suggesting that the drop in survival is more pronounced in males, in agreement with epidemiological data of chronic liver disease in MASLD and HCC patients (Vernon et al, 2011; El-Serag et al, 2003). Given this sex-dependent effect, we selected male mice for our follow-up experiments. *Oma1*^KO^ early mortality was paralleled by a progressive reduction in body weight (Fig. EV1B). However, *Oma1*^KO^ mice did not show an accelerated aging phenotype according to functional tests assessing frailty (Fig. EV1C,D).

Histological and macroscopic analyses from aged *Oma1*^KO^ dying animals revealed an unusually high incidence of liver fibrosis and tumors earlier in time (Fig. EV1E,F), representing their most likely cause of death. C57BL/6 mice are known to naturally develop hepatic tumors during aging (Nakamura et al, 1992), but in the absence of OMA1 its onset seemed to be accelerated. To discern if primary hepatic tumor incidence was in fact increased in aged *Oma1*^KO^ mice, we euthanized and analyzed 90-week-old animals. *Oma1*^KO^ primary hepatic tumor incidence was 71.43%, while control mice did not present hepatic primary tumors yet (Fig. 2B). Among these, the most common tumor type was hepatoadenoma (42.85%), followed by hemangiosarcoma (28.57%) and histiocytic sarcoma (14.28%), as revealed by the histopathological analysis (Figs. 2B,C and  EV1G). Some mice presented various hepatic primary tumors simultaneously. With time, *Oma1*^KO^ hepatoadenoma likely progresses to hepatocellular carcinoma, as accounted for the autopsies of *Oma1*^KO^ mice dying in survival experiments (Fig. 2G).

Bulk transcriptomics and proteomics analyses from 90-week-old liver tissue confirmed a proinflammatory, fibrotic and tumorigenic signature in *Oma1*^KO^ mice (Fig. 2D). Tumoral *Oma1*^KO^ livers presented a metabolic shutdown and a reduction in their glutathione-mediated detoxification ability, together with an increase in the ferroptosis pathway (Fig. 2D). Regarding the role of the immune system in the *Oma1*^KO^ tumoral microenvironment, we found that *Oma1*^KO^ cells promoted an inhibitory action on T cells through the PD-1/PD-L1 pathway and that macrophages were prominently activated (Fig. 2D). *Oma1*^KO^ tumoral livers had an increased tendency to suffer the epithelial to mesenchymal transition by growth factors (Fig. 2D).

P53 was the most upregulated upstream transcription regulator according to both transcriptomics and proteomics data (Fig. 2E,F). In turn, the most downregulated upstream transcription regulator was HNF4α (Fig. 2F), whose levels tend to decrease with liver function deterioration. Among the upstream cytokine regulators, we found an increase in TNF, IL6, IFNG, or IL4 (Fig. 2F).

To ascertain whether the absence of OMA1 accelerates tumorigenesis by enhancing tumor aggressiveness independently of the tissue, we tried two different tumor models: an allograft and a chemically induced tumor. For the allograft tumor model, we generated and injected *Oma1*^WT^ or *Oma1*^KO^ E0771 mouse breast cancer cells in the flanks of immunocompromised mice (Fig. EV1H).

Allografts reached equivalent sizes independently of the presence or the absence of OMA1 (Fig. EV1H), suggesting that tumor growth is not inherently accelerated by the deletion of OMA1. For the chemically induced tumor model, we injected intramuscularly 3-methylcholanthrene (3MC) in the constitutive *Oma1*^WT^ or *Oma1*^KO^ left legs (Fig. EV1I). Fibrosarcoma reached equivalent sizes independently of the genotype (Fig. EV1I), showing that tumor growth was not enhanced by the deletion of OMA1, both in the tumor and in its environment.

These results show that the relation between OMA1 and tumorigenesis is not strictly direct and universal, but rather cell-type specific and, probably, very dependent on the context found in the liver.

### Early liver dysfunction in young *Oma1*^KO^ mice

**Figure 3:**
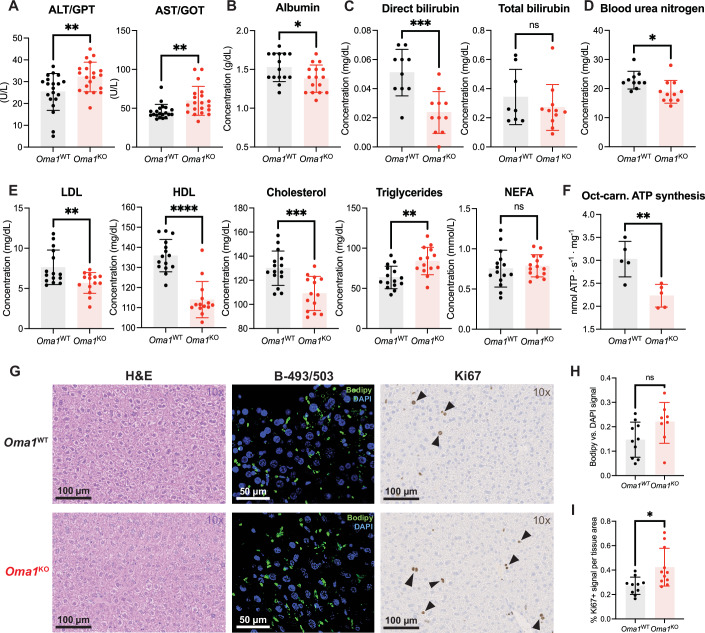
Early liver dysfunction in young *Oma1*^KO^ mice. (**A**) Plasma ALT-GPT (*P* = 0.0074), AST-GOT (*P* = 0.0021) from 10-week-old male *Oma1*^WT^ (*n* = 20) and *Oma1*^KO^ (*n* = 20) mice. “AST-GOT” values did not pass normality, while “ALT-GPT” passed normality. (**B**) Plasma albumin (*P *= 0.0319) from 10-week-old male *Oma1*^WT^ (*n* = 15) and *Oma1*^KO^ (n = 16) mice. “Albumin” values passed normality. (**C**) Plasma direct bilirubin (*P* = 0.0006) and total bilirubin from 10-week-old male *Oma1*^WT^ (*n* = 10) and *Oma1*^KO^ (*n* = 11) mice. “Direct bilirubin” values passed normality, while “Total bilirubin” data did not pass normality. (**D**) Plasma BUN (0.0179) from 10-week-old male *Oma1*^WT^ (*n* = 10) and *Oma1*^KO^ (*n* = 11) mice. “BUN” values passed normality. (**E**) Plasma LDL (*P* = 0.0068), HDL (*P* < 0.0001), total cholesterol (*P* = 0.0005), NEFAs and TAGs (0.0014) in 10-week-old male *Oma1*^WT^ (*n* = 15), and *Oma1*^KO^ (*n* = 14) animals fasted for 6 h. “HDL” values did not pass normality, while “LDL”, “Cholesterol”, “Triglycerides”, and “NEFA” passed normality. (**F**) ATP synthesis production using octanoyl carnitine (*P* = 0.0063) in liver isolated mitochondria from 10-week-old fed male *Oma1*^WT^ (*n* = 5) and *Oma1*^KO^ (*n* = 5) mice. “Oct-carn. ATP synthesis” values passed normality. (**G**) Representative H&E, bodipy, and Ki67 staining analysis of 10-week-old fed *Oma1*^WT^ (*n* = 10) and *Oma1*^KO^ (*n* = 11) paraffin liver sections or whole-mount liver samples. Arrows indicate Ki67 staining-positive areas in liver tissue. (**H**) Bodipy fluorescence signal quantification (*P* = 0.1238) in 10 weeks-old fed male *Oma1*^WT^ (*n* = 10) and *Oma1*^KO^ (*n* = 8) whole-mount liver samples. “Bodipy signal” values passed normality. (**I**) Ki67 signal quantification (*P* = 0.0127) in 10-week-old fed male *Oma1*^WT^ (*n* = 10) and *Oma1*^KO^ (*n* = 11) paraffin liver sections. “%Ki67 signal” values did not pass normality. *P* value from unpaired two-tailed Student’s *t* test (when data passed normality) or Mann–Whitney test (when data did not pass normality) is represented in each graph. ns *P* > 0.05, ^∗^*P* < 0.05, ^∗∗^*P* < 0.01, ^∗∗∗^*P* < 0.001, and ^∗∗∗∗^*P* < 0.0001. Source data are available online for this figure.

We analyzed young *Oma1*^KO^ mice (at 9–14 weeks old) to find out whether the appearance of tumors during aging was preceded by an early impairment of liver function. Blood plasma analyses in 10-week-old *Oma1*^KO^ mice revealed evidence of liver damage, as shown by their elevated plasma transaminases (Fig. 3A), decrease in plasma albumin (Fig. 3B), direct bilirubin (Fig. 3C), and blood urea nitrogen (Fig. 3D), whose levels are tightly dependent on liver function.

**Figure EV2:**
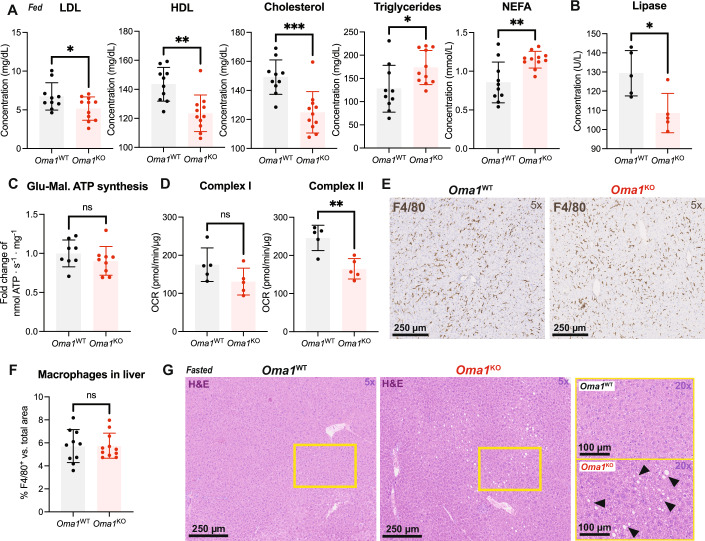
Early liver dysfunction in young *Oma1*^KO^ mice. (**A**) Fed-state values of plasma LDL (*P* = 0.0410), HDL (*P* = 0.0014), total cholesterol (*P* = 0005), NEFAs (*P* = 0.0029), and TAGs (*P* = 0.0272) in 10-week-old male *Oma1*^WT^ (*n* = 10) and *Oma1*^KO^ (*n* = 11) mice (mean ± SD). (**B**) Plasma lipase (*P* = 0.0180) in 10-week-old male *Oma1*^WT^ (*n* = 5) and *Oma1*^KO^ (*n* = 5) mice. (**C**) ATP synthesis production using glutamate-malate in liver isolated mitochondria from 10-week-old male mice fed *Oma1*^WT^ (*n* = 8) and *Oma1*^KO^ (*n* = 9) mice (mean ± SD). (**D**) Basal oxygen consumption rate from complex I and complex II (*P* = 0.0028) substrates in frozen isolated mitochondria from male *Oma1*^WT^ (*n* = 5) and *Oma1*^KO^ (*n* = 5) livers (left). Complex II-driven OCR seahorse profile (right) (mean ± SD). (**E**) Representative F4/80 staining analysis of 10-week-old male fed *Oma1*^WT^ (*n* = 10) and *Oma1*^KO^ (*n* = 11) paraffin liver sections. (**F**) F4/80 staining analysis of 10-week-old male fed *Oma1*^WT^ (*n* = 10) and *Oma1*^KO^ (*n* = 11) paraffin liver sections (mean ± SD). (**G**) Representative H&E staining analysis of 6-h fasted 10-week-old male *Oma1*^WT^ (*n* = 10) and *Oma1*^KO^ (*n* = 11) paraffin liver sections. Magnifications are depicted to the right to better visualize lipid droplets (black arrows) in fasted livers. *P* value from unpaired two-tailed Student’s *t* test (when data passed normality) or Mann–Whitney test (when data did not pass normality) is represented in each graph. ns *P* > 0.05, ^∗^*P* < 0.05, ^∗∗^*P* < 0.01, ^∗∗∗^*P* < 0.001, and ^∗∗∗∗^*P* < 0.0001.

In fasted 10-week-old *Oma1*^KO^ animals, plasma lipoprotein levels were reduced—especially HDL—and circulating triglycerides were increased when compared to their controls (Fig. 3E). Fed-*Oma1*^KO^ animals presented an equivalent lipid profile in plasma (Fig. EV2A). Interestingly, circulating lipase was decreased in *Oma1*^KO^ mice (Fig. EV2B). Ex vivo ATP synthesis analysis showed that mitochondria isolated from 10-week-old *Oma1*^KO^ mice livers presented a specific decrease in β-oxidation, as they produced less ATP from octanoyl-carnitine (Fig. 2F), while no reduction by complex I-dependent substrates was observed (Fig. EV2C). Respirometry assays confirmed the functionality of complex I-mediated respiration but showed a reduction in complex II-driven respiration (Fig. EV2D).

Histological and immunofluorescence analyses showed no evident accumulation of fat in fed 10-week-old *Oma1*^KO^ livers (Fig. 3G,H). However, after 6 h of fasting, *Oma1*^KO^ mice accumulated fat in bigger lipid droplets than their controls (Fig. EV2G), evidencing their lipid metabolism dysregulation. Histological analysis revealed an abnormally high cellular proliferation rate in 10-week-old *Oma1*^KO^ livers (Fig. 3I), whose nature was not further determined. At this age, the total amount of macrophages in the liver was not different between genotypes (Fig. EV2E,F).

These data suggest that *Oma1* ablation in young animals is associated with early liver proliferative lesions, as well as impairment of hepatic lipid utilization, plasma lipoprotein levels, and overall liver function.

### Increased oxidative stress and cell death in young *Oma1*^KO^ livers

**Figure EV3:**
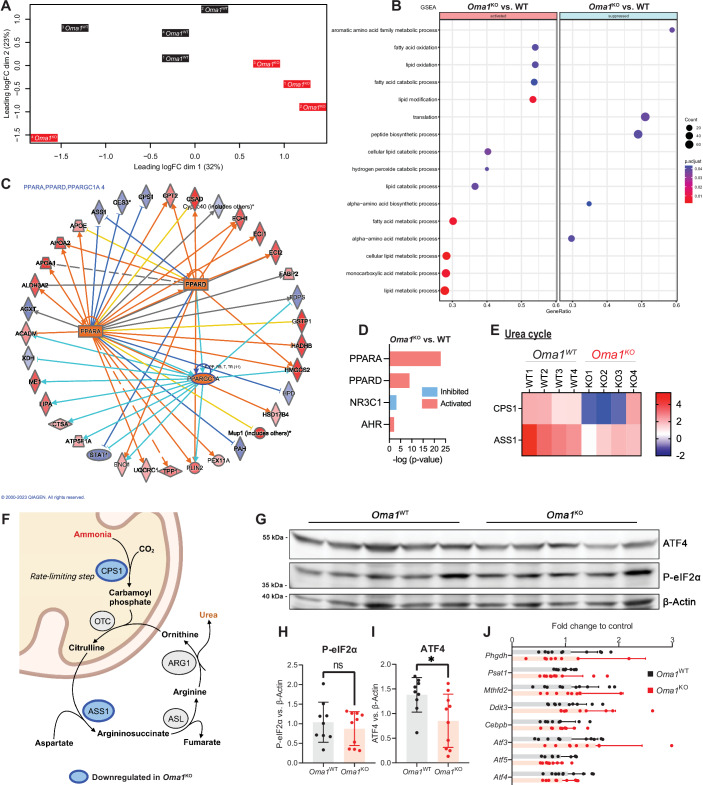
Increased oxidative stress and liver degeneration in young *Oma1*^KO^ livers. (**A**) Principal Component Analysis using proteomics data from 9-week-old *Oma1*^WT^ (*n* = 4) and *Oma1*^KO^ (*n* = 4) liver samples. (**B**) Gene Set Enrichment Analysis using proteomics data from 9-week-old male *Oma1*^WT^ (*n* = 4) and *Oma1*^KO^ (*n* = 4) liver samples. Kolmogorov–Smirnov test (one-sided) and Benjamini–Hochberg method, adjusted *P* value < 0.05. (**C**) Gene networks under the control of the predicted upstream regulators PPARA, PPARD, and PPARGC1A according to IPA analysis using proteomics data from 9-week-old male *Oma1*^KO^ (*n* = 4) liver proteomics samples compared to *Oma1*^WT^ (*n* = 4) mice. (**D**) IPA-obtained upstream regulators in young 9-week-old male *Oma1*^KO^ (*n* = 4) mice compared to *Oma1*^WT^ (*n* = 4) animals filtered by ligand-dependent nuclear receptors. Benjamini–Hochberg method, adjusted *P* value < 0.05. (**E**) Proteomics heatmap of urea cycle gene expression in young 9-week-old male *Oma1*^KO^ (*n* = 4) mice compared to *Oma1*^WT^ (*n* = 4) animals. (**F**) Simplified representation of the urea cycle showing the downregulated proteins according to proteomics in 9-week-old male *Oma1*^KO^ (*n* = 4) mice compared to *Oma1*^WT^ (*n* = 4) animals. (**G**) Representative western blot analysis of ATF4 and P-eIF2α protein signal from young 10 weeks-old male *Oma1*^WT^ (*n* = 5) and *Oma1*^KO^ (*n* = 5) homogenate liver samples, using β-Actin as a loading control. (**H**) Quantification of relative P-eIF2α to β-Actin protein signal (*P* = 0.7802) according to Western Blot analyses from young 10-week-old male *Oma1*^WT^ (*n* = 9) and *Oma1*^KO^ (*n* = 10) homogenate liver samples. The data did not pass normality. *P* value from the Mann–Whitney test is represented in the graph. (**I**) Quantification of relative ATF4 to β-Actin protein signal (*P* = 0.0223) according to western blot analyses from young 10-week-old *Oma1*^WT^ (*n* = 9) and *Oma1*^KO^ (*n* = 10) homogenate liver samples. Data passed normality. *P* value from Welch’s *t* test (SDs differed) is represented in the graph. (**J**) Relative expression of *Atf4* and *Atf4*-dependent transcriptional targets in young 10 weeks-old *Oma1*^WT^ (*n* = 10) and *Oma1*^KO^ (*n* = 10) liver samples. ns *P* > 0.05, ^∗^*P* < 0.05, ^∗∗^*P* < 0.01, ^∗∗∗^*P* < 0.001, and ^∗∗∗∗^*P* < 0.0001.

To understand the molecular mechanism behind the young *Oma1*^KO^ liver dysfunction, we performed proteomics analyses on 9-week-old *Oma1*^WT^ and *Oma1*^KO^ livers (Fig. EV3A). Lipid metabolism dysregulation in 9-week-old *Oma1*^KO^ livers was confirmed by gene set enrichment analysis (GSEA) and Ingenuity Pathways Analysis (IPA) (Fig. EV3B,C). Ligand-dependent nuclear receptors PPARA, PPARD, and PPARGC1A were predicted to govern this lipid dysregulation (Fig. EV3D). Interestingly, a marked decrease in the urea cycle was observed in 9-week-old *Oma1*^KO^ livers (Fig. EV3E,F), which may compromise the detoxification ability of *Oma1*^KO^ hepatocytes.

**Figure 4:**
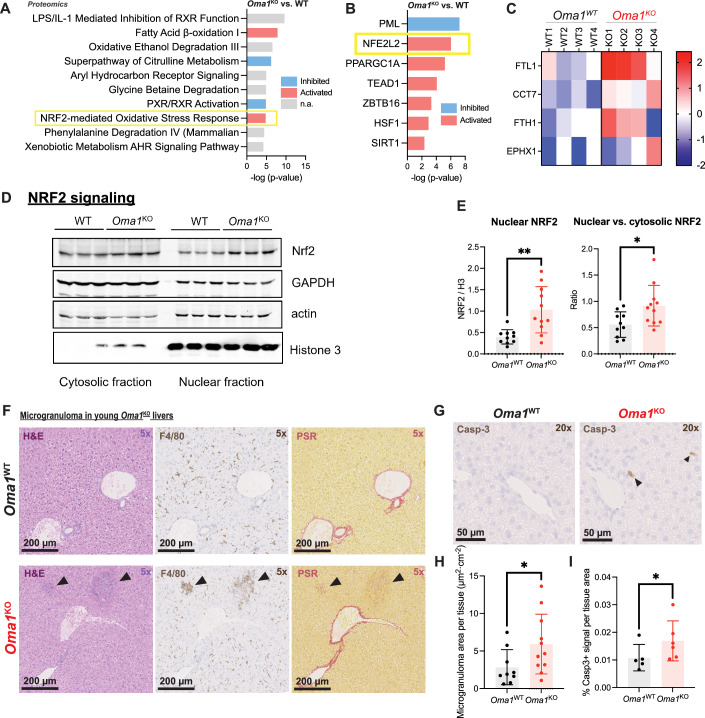
Increased oxidative stress and liver degeneration in young *Oma1*^KO^ livers. (**A**) IPA-obtained top significantly different pathways using proteomics data in young 9-week-old male *Oma1*^KO^ (*n* = 4) mice compared to *Oma1*^WT^ (*n* = 4) animals. Benjamini–Hochberg method, adjusted *P* value < 0.05. (**B**) IPA-obtained upstream regulators in young male 9-week-old *Oma1*^KO^ (*n* = 4) mice compared to *Oma1*^WT^ (*n* = 4) animals filtered by transcription factors. Benjamini–Hochberg method, adjusted *P* value < 0.05. (**C**) Heatmap from proteomics data of NRF2-target genes expression in young 10-week-old male *Oma1*^KO^ (*n* = 4) mice compared to *Oma1*^WT^ (*n* = 4) animals. (**D**) Representative western blot analysis of NRF2 protein signal in the nuclear and cytosolic subcellular fractions from young 10 weeks-old male *Oma1*^WT^ (*n* = 5) and *Oma1*^KO^ (*n* = 6) liver samples, including GAPDH and H3 as subcellular location-specific loading controls. (**E**) NRF2 subcellular localization quantification according to Western Blot analysis from young 10-week-old male *Oma1*^WT^ (*n* = 10) and *Oma1*^KO^ (*n* = 11) mice. “Nuclear NRF2” and “Nuclear vs. cytosolic NRF2” values passed normality. *P* values from unpaired two-tailed Student’s *t* tests are represented in each graph. (**F**) Representative H&E, F4/80, and PSR-stained images from multifocal liver granuloma in young 10-week-old male *Oma1*^KO^ mice compared to *Oma1*^WT^ animals. Arrows indicate liver microgranuloma lesions. (**G**) Representative Caspase-3 staining analysis in liver paraffin sections from young 10 weeks-old male *Oma1*^WT^ and *Oma1*^KO^ mice. Arrows indicate Caspase-3 staining-positive areas. (**H**) Relative multifocal liver granuloma areas to total tissue quantification in young 10-week-old male *Oma1*^KO^ (*n* = 11) mice compared to *Oma1*^WT^ (*n* = 10) animals. “Relative microgranuloma area” values did not pass normality. *P* value from a Mann–Whitney test is represented in the graph. (**I**) Caspase-3 staining analysis quantification in liver paraffin sections from young 10-week-old male *Oma1*^WT^ (*n* = 5) and *Oma1*^KO^ (*n* = 6) mice. *P* value from a Kolmogorov–Smirnov test is represented in the graph. ns *P* > 0.05, ^∗^*P* < 0.05, ^∗∗^*P* < 0.01, ^∗∗∗^*P* < 0.001, and ^∗∗∗∗^*P* < 0.0001. Source data are available online for this figure.

According to IPA analysis, the most upregulated pathway in the *Oma1*^KO^ liver proteome was the “NRF2-mediated oxidative stress response” pathway (Fig. 4A). In fact, NRF2 was the top transcriptional upstream regulator in *Oma1*^KO^ livers predicted to explain the proteomic changes (Fig. 4B) and various transcriptional NRF2 targets—including FTL1, CCT7, FTH1 and EPHX1—were significantly upregulated (Fig. 4C). The activation of the oxidative stress response mediated by NRF2 was confirmed using Western Blot following subcellular fractionation of liver tissue (Fig. 4D). *Oma1*^KO^ mice presented an enhanced nuclear localization of NRF2 (Fig. 4E), whose translocation to the nucleus is known to occur upon oxidative stress. Therefore, we confirmed that the absence of OMA1 activates this oxidative stress pathway in the liver.

Given the known link between OMA1 and the integrated stress response through the OMA1-DELE1-HRI axis, we studied the phosphorylation of eIF2α and the levels of ATF4 protein (Fig. EV3G). We found no significant differences in the amount of P-eIF2α (Fig. EV3H). ATF4 protein levels were subtly reduced (Fig. EV3I), but the expression levels of its classical transcriptional targets were not altered (Fig. EV3J), implying that basal OMA1-mediated ISR is not functionally altered in this hepatic phenotype.

As a proxy for liver damage, we analyzed the incidence of liver microgranuloma in young *Oma1*^WT^ and *Oma1*^KO^ mice through histopathology. These histological findings are typical of liver injury (Lukita-Atmadja et al, 1993) or liver infection models (Martino et al, 2005). We found an increase in microgranuloma lesions in young *Oma1*^KO^ mice (Fig. 4F), who presented bigger areas of cell clumps containing infiltrated macrophages. Caspase-3 staining revealed that *Oma1*^KO^ livers presented a clear but not very extensive increase in liver cell apoptosis (Fig. 4G,H), confirming that oxidative stress-exposed *Oma1*^KO^ livers are more prone to cell death and liver damage.

### Chronic liver inflammation and fibrosis in *Oma1*^KO^ mice

**Figure 5:**
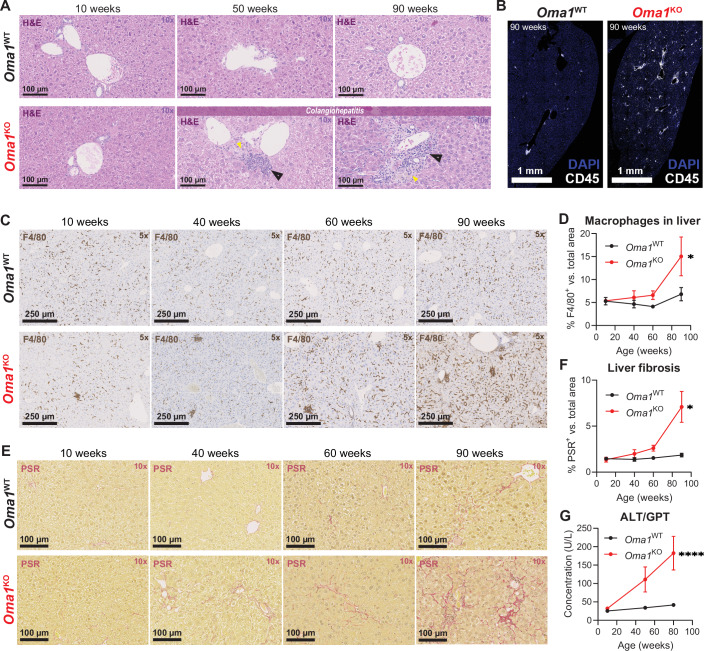
Chronic liver inflammation and fibrosis in *Oma1*^KO^ mice. (**A**) Representative H&E analysis of cholangiohepatitis (black and yellow arrows are used to delimit the area of interest) initiation and progression in male *Oma1*^KO^ tumor-free livers compared to *Oma1*^WT^ animals at increasing ages, including 10, 50, and 90 weeks of age. (**B**) Representative CD45-stained immunofluorescence images from 90-week-old male *Oma1*^WT^ and *Oma1*^KO^ tumor-free liver tissue. (**C**) Representative F4/80 staining analysis of 10, 40, 60, and 90 weeks-old male *Oma1*^WT^ and *Oma1*^KO^ tumor-free liver tissue. (**D**) Relative F4/80-stained area to whole liver tissue area quantification (p = 0.0360) from 10, 40, 60, and 90 weeks-old *Oma1*^WT^ and *Oma1*^KO^ livers. Mice cohorts at the analyzed time points were not the same individuals. Graphical representation using continuous lines has been chosen for visual simplicity. The number of animals used at these 10, 40, 60, and 90 weeks of age cohorts were *Oma1*^WT^: *n* = 5, *n* = 5, *n* = 9, and *n* = 4, respectively; and *Oma1*^KO^: *n* = 5, *n* = 4, *n* = 9, and *n* = 7, respectively. *P* value from a two-way ANOVA testing “OMA1 expression” as a source of variation is represented in the graph. (**E**) Representative PSR staining analysis of 10, 40, 60, and 90 weeks-old male *Oma1*^WT^ and *Oma1*^KO^ tumor-free liver tissue. (**F**) Relative PSR-stained area to whole liver tissue area quantification (*P* = 0.0138) from 10, 40, 60, and 90 weeks-old *Oma1*^WT^ and *Oma1*^KO^ livers. Mice cohorts at the analyzed time points were not the same individuals. Graphical representation using continuous lines has been chosen for visual simplicity. The number of animals used at these 10, 40, 60, and 90 weeks of age cohorts were *Oma1*^WT^: *n* = 5, *n* = 5, *n* = 5, and *n* = 4, respectively; and *Oma1*^KO^: *n* = 5, *n* = 4, *n* = 4, and *n* = 7, respectively. *P* value from a two-way ANOVA testing “OMA1 expression” as a source of variation is represented in the graph. (**G**) Plasma ALT-GPT values (*P* < 0.0001) from 10, 50, and 80-week-old male *Oma1*^WT^ and *Oma1*^KO^ mice. Mice cohorts at the analyzed time points were not the same individuals. Graphical representation using continuous lines has been chosen for visual simplicity. The number of animals used at these 10, 50, and 80 weeks of age cohorts were *Oma1*^WT^: *n* = 20, *n* = 10, and *n* = 5, respectively; and *Oma1*^KO^: *n* = 20, *n* = 12, and *n* = 8, respectively. *P* value from a two-way ANOVA testing “OMA1 expression” as a source of variation is represented in the graph. ns *P* > 0.05, ^∗^*P* < 0.05, ^∗∗^*P* < 0.01, ^∗∗∗^*P* < 0.001, and ^∗∗∗∗^*P* < 0.0001. Source data are available online for this figure.

**Figure EV4:**
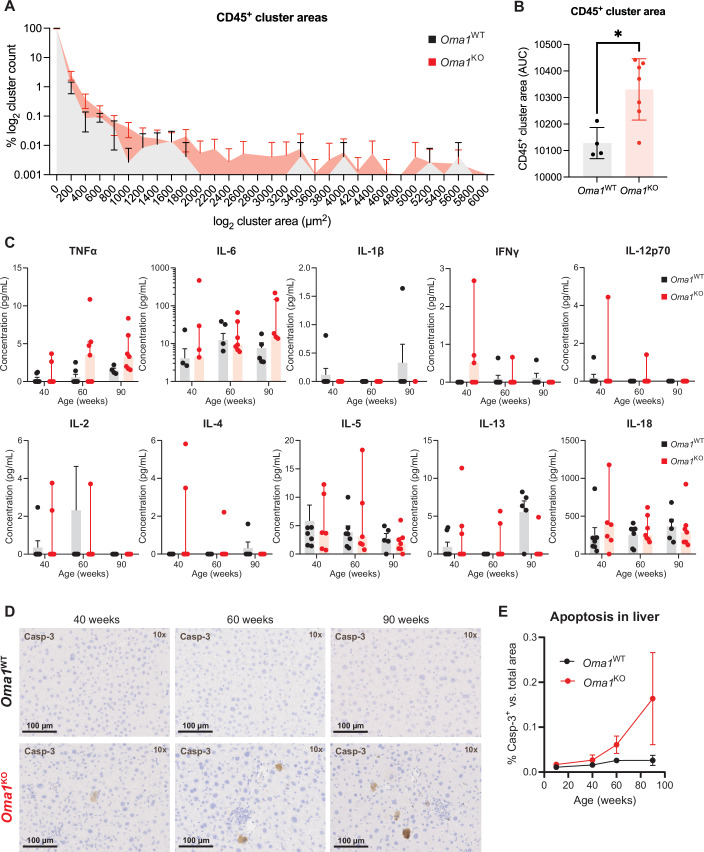
Chronic liver inflammation and fibrosis in *Oma1*^KO^ mice. (**A**) CD45^+^ cluster areas histogram from 90-week-old male *Oma1*^WT^ (*n* = 4) and *Oma1*^KO^ (*n* = 7) immunofluorescence liver images (mean ± SD). (**B**) Area under the curve (AUC) from CD45^+^ cluster areas histograms using 90-week-old male *Oma1*^WT^ (*n* = 4) and *Oma1*^KO^ (*n* = 7) immunofluorescence liver images (mean ± SD). Data passed normality, *P* value from unpaired two-tailed Student’s *t* test is represented in the graph. (**C**) Plasma cytokine values from 40, 60, and 90-week-old male *Oma1*^WT^ and *Oma1*^KO^ mice. Mice cohorts at the analyzed time points were not the same individuals. The number of animals used at these 40, 60, and 90 weeks of age cohorts were *Oma1*^WT^: *n* = 7, *n* = 7, and *n* = 5, respectively; and *Oma1*^KO^: *n* = 7, *n* = 7, and *n* = 7, respectively (mean ± SD). Two-way ANOVA. (**D**) Representative Caspase-3 staining analysis of 10, 40, 60, and 90-week-old male *Oma1*^WT^ and *Oma1*^KO^ tumor-free liver tissue. (**E**) Relative Caspase-3-stained area to whole liver tissue area quantification from 10, 40, 60, and 90-week-old *Oma1*^WT^ and *Oma1*^KO^ livers. Mice cohorts at the analyzed time points were not the same individuals. Graphical representation using continuous lines has been chosen for visual simplicity. The number of animals used at these 10, 40, 60, and 90 weeks of age cohorts were *Oma1*^WT^: *n* = 5, *n* = 5, *n* = 5, and *n* = 4, respectively; and *Oma1*^KO^: *n* = 6, *n* = 4, *n* = 4, and *n* = 7, respectively (mean ± SD). *P* value from a two-way ANOVA testing “OMA1 expression” as a source of variation is represented in the graph. ns *P* > 0.05, ^∗^*P* < 0.05, ^∗∗^*P* < 0.01, ^∗∗∗^*P* < 0.001, and ^∗∗∗∗^*P* < 0.0001.

Next, we characterized the potential progression of liver damage in *Oma1*^KO^ animals with age. We determined that *Oma1*^KO^ liver injury progressed over time until the development of cholangiohepatitis (Fig. 5A), from 50 weeks of age onwards. Using immunofluorescence, we identified CD45^+^ cells at these inflammatory sites, implying an increased immune infiltration (Fig. 5B). CD45^+^ signal quantification showed that *Oma1*^KO^ immune clusters in the liver were bigger in size (Fig. EV4A,B). At early stages of *Oma1*^KO^ cholangiohepatitis, no primary hepatic liver tumors were detected through histopathological examination, suggesting that hepatic immune infiltration in aged *Oma1*^KO^ mice precedes tumorigenesis.

Along with the development of cholangiohepatitis, macrophages also accumulated in *Oma1*^KO^ livers with age, clustering increasingly at microgranuloma areas (Fig. 5C,D). To determine whether *Oma1*^KO^ age-related inflammation was circumscribed to the liver or rather a systemic hyperinflammatory effect, we measured circulating cytokines in *Oma1*^WT^ and *Oma1*^KO^ plasma samples from increasing age. No changes in circulating cytokines were detected in *Oma1*^KO^ plasma samples with age (Fig. EV4C), meaning that this is a local inflammatory phenomenon.

To study the potential progression of hepatic fibrosis, we used Picro-Sirius Red staining on *Oma1*^WT^ and *Oma1*^KO^ liver paraffin sections. We observed that in parallel to inflammation, hepatic fibrosis was increasingly present in *Oma1*^KO^ mice (Fig. 5E,F). Caspase-3 staining also increased with aging in *Oma1*^KO^ livers (Fig. EV4D,E). Accordingly, *Oma1*^KO^ ALT-GPT plasma levels massively rose over time (Fig. 5G), evidencing the chronicity and severity progression of liver injury in Oma1^KO^ animals with age.

### Progressive immune exhaustion in *Oma1*^KO^ livers

**Figure EV5:**
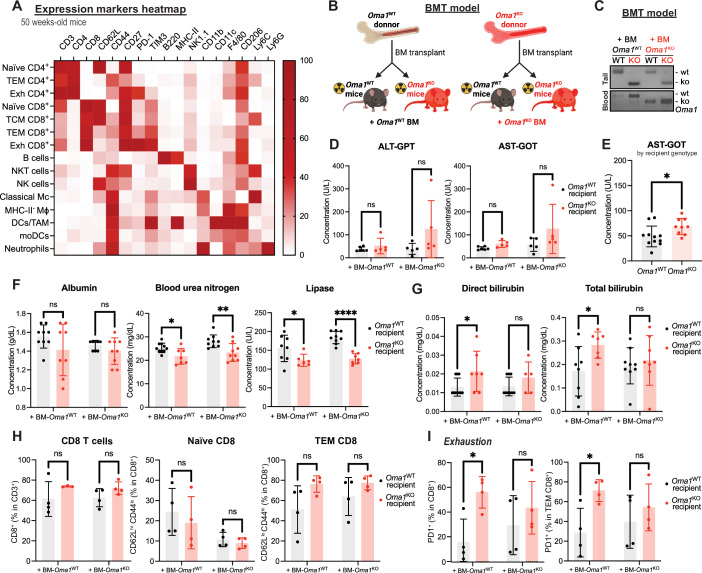
Progressive immune exhaustion in *Oma1*^KO^ livers. (**A**) Expression markers heatmap from 50-week-old male *Oma1*^WT^ (*n* = 4) and *Oma1*^KO^ (*n* = 5) mice, assigning expression patterns to diverse immune populations using OMIQ. (**B**) “BMT model” experimental design scheme, depicting *Oma1*^WT^ and *Oma1*^KO^ bone marrow transfer to male *Oma1*^WT^ (*n* = 10 and *n *= 9, respectively) and *Oma1*^KO^ (*n* = 9 and *n* = 9, respectively) lethally irradiated mice. (**C**) *Oma1* genotype in tail and blood samples from male irradiated *Oma1*^WT^ and *Oma1*^KO^ mice transferred with *Oma1*^WT^ and *Oma1*^KO^ BM. (**D**) Plasma ALT-GPT and AST-GOT values from 40-week-old male irradiated *Oma1*^WT^ and *Oma1*^KO^ mice transferred with *Oma1*^WT^ (*n* = 5 and *n* = 5, respectively) and *Oma1*^KO^ (*n* = 5 and *n* = 5, respectively) BMs. *P* values from Šídák’s multiple comparisons tests comparing *Oma1*^WT^ and *Oma1*^KO^ recipient animals in the presence of each bone marrow are represented in each graph. (**E**) Plasma AST-GOT values (*P* = 0.0313) from 40-week-old male irradiated *Oma1*^WT^ and *Oma1*^KO^ mice, independently of the bone marrow genotype (*Oma1*^WT^ or *Oma1*^KO^) they were transferred with. “AST-GOT” values passed normality. (**F**) Plasma albumin, blood urea nitrogen ( + BM-*Oma1*^WT^ *P* = 0.0411, +BM-*Oma1*^KO^ *P* = 0.0038) and lipase ( + BM-*Oma1*^WT^ *P* = 0.0306, +BM-*Oma1*^KO^ *P* < 0.0001) values from 20 weeks-old male irradiated *Oma1*^WT^ and *Oma1*^KO^ mice transferred with *Oma1*^WT^ (*n* = 10 and *n* = 9, respectively) and *Oma1*^KO^ (*n* = 9 and *n* = 9, respectively) BMs. *P* values from Šídák’s multiple comparisons tests comparing *Oma1*^WT^ and *Oma1*^KO^ recipient animals in the presence of each bone marrow are represented in each graph. (**G**) Plasma direct bilirubin ( + BM-*Oma1*^WT^ *P* = 0.0470) and total bilirubin values (+BM-*Oma1*^WT^ *P* = 0.0440) from 20-week-old male irradiated *Oma1*^WT^ and *Oma1*^KO^ mice transferred with *Oma1*^WT^ (*n* = 10 and *n* = 9, respectively) and *Oma1*^KO^ (*n* = 9 and *n* = 9, respectively) BMs. *P* values from Šídák’s multiple comparisons tests comparing *Oma1*^WT^ and *Oma1*^KO^ recipient animals in the presence of each bone marrow are represented in each graph. (**H**) Liver CD8^+^ T cells populations from 20-week-old male irradiated *Oma1*^WT^ and *Oma1*^KO^ mice transferred with *Oma1*^WT^ (*n* = 10 and *n* = 9, respectively) and *Oma1*^KO^ (*n* = 9 and *n* = 9, respectively) BMs, including percentages of total, naive and TEM CD8^+^ T cell populations. *P* values from Šídák’s multiple comparisons tests comparing *Oma1*^WT^ and *Oma1*^KO^ recipient animals in the presence of each bone marrow are represented in each graph. (**I**) Percentages of exhausted (PD-1^+^) liver CD8^+^ T cells ( + BM-*Oma1*^WT^ *P* = 0.0267) and TEM CD8^+^ cells ( + BM-*Oma1*^WT^ *P* = 0.0393) populations from 20 weeks-old male irradiated *Oma1*^WT^ and *Oma1*^KO^ mice transferred with *Oma1*^WT^ (*n* = 10 and *n* = 9, respectively) and *Oma1*^KO^ (*n* = 9 and *n* = 9, respectively) BMs. *P* values from Šídák’s multiple comparisons tests comparing *Oma1*^WT^ and *Oma1*^KO^ animals within every cell population are represented in each graph. ns *P* > 0.05, ^∗^*P* < 0.05, ^∗∗^*P* < 0.01, ^∗∗∗^*P* < 0.001, and ^∗∗∗∗^*P* < 0.0001.

**Figure 6:**
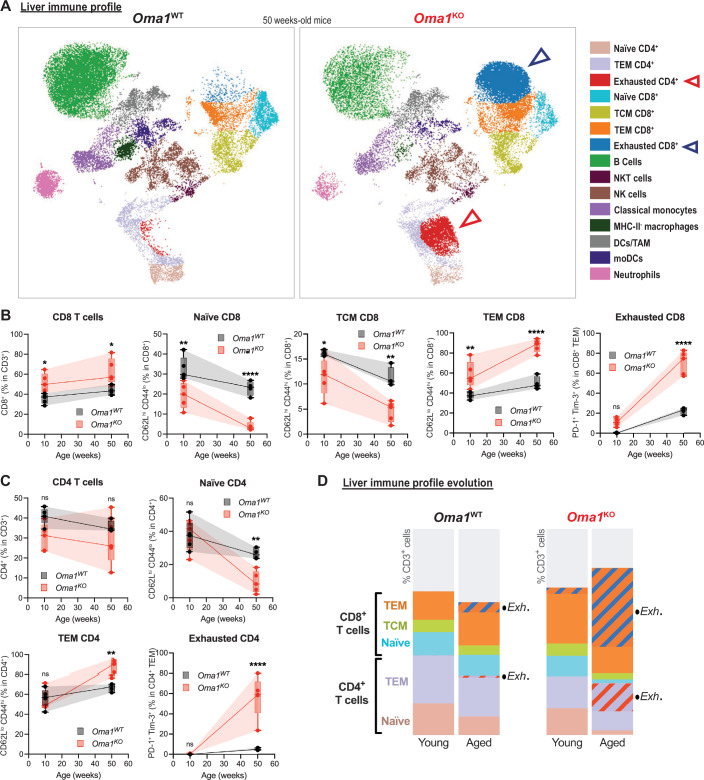
Progressive immune exhaustion in *Oma1*^KO^ livers. (**A**) Representative UMAP plot of the liver immune profile from 50-week-old male *Oma1*^WT^ (*n* = 4) and *Oma1*^KO^ (*n* = 5) mice. Spatial single-cell plot location was assigned according to the individual expression pattern of 18 surface markers. (**B**) Liver CD8^+^ T cells populations’ shift in aging male *Oma1*^WT^ and *Oma1*^KO^ mice, including percentage of total CD8^+^ T cells in CD3^+^ cells (14w *P* = 0.0295 and 50w *P* = 0.0147), percentages of naive (14w *P* = 0.0013, 50w *P* < 0.0001), TCM (14w *P* = 0.0321, 50w *P* = 0.0026) and TEM CD8^+^ T cells populations (14w *P* = 0.0024, 50w *P* < 0.0001) and percentage of exhausted (PD-1^+^ and Tim-3^+^) TEM CD8^+^ T cells (14w *P* = 0.8191, 50w *P* = 0.0004). Mice cohorts at the analyzed time points were not the same individuals. Graphical representation using continuous lines has been chosen for visual simplicity. The number of animals used at these 14 and 50 weeks of age cohorts were *Oma1*^WT^: *n* = 5 and *n* = 4, respectively; and *Oma1*^KO^: *n* = 5 and *n* = 5, respectively. Boxes represent the 25th to 75th percentiles, with the median in the center and whiskers extending to the minimum and maximum data points. Ordinary two-way ANOVA was performed with Šídák’s multiple comparisons tests to estimate significant differences comparing *Oma1*^WT^ and *Oma1*^KO^ animals at every timepoint are represented in each graph. (**C**) Liver CD4^+^ T cells populations’ shift in aging male *Oma1*^WT^ and *Oma1*^KO^ mice, including percentage of total CD4^+^ T cells in CD3^+^ cells (14w *P* = 0.0892, 50w *P* = 0.3846), percentages of naive (14w *P* = 0.9940, 50w *P* = 0.0093) and TEM CD4^+^ T cells (14w *P* = 0.8800, 50w *P* = 0.0083) populations and percentage of exhausted (PD-1^+^ and Tim-3^+^) TEM CD4^+^ T cells (14w *P* = 0.9986, 50w *P* < 0.0001). Mice cohorts at the analyzed time points were not the same individuals. Graphical representation using continuous lines has been chosen for visual simplicity. The number of animals used at these 14 and 50 weeks of age cohorts were *Oma1*^WT^: *n* = 5 and *n* = 4, respectively; and *Oma1*^KO^: *n* = 5 and *n* = 5, respectively. Boxes represent the 25th to 75th percentiles, with the median in the center and whiskers extending to the minimum and maximum data points. Ordinary two-way ANOVA was performed with Šídák’s multiple comparisons tests to estimate significant differences comparing *Oma1*^WT^ and *Oma1*^KO^ animals at every timepoint are represented in each graph. Comparing *Oma1*^WT^ and *Oma1*^KO^ animals at every timepoint are represented in each graph. (**D**) Scaled graphical summary of CD8^+^ and CD4^+^ T cell populations’ shift relative to CD3^+^ cells in *Oma1*^WT^ and *Oma1*^KO^ mice^,^ comparing young (14-week-old) vs. aged (50-week-old) liver immune profiles. ns *P* > 0.05, ^∗^*P* < 0.05, ^∗∗^*P* < 0.01, ^∗∗∗^*P* < 0.001, and ^∗∗∗∗^*P* < 0.0001. Source data are available online for this figure.

Given that immune infiltration was playing a major role in *Oma1*^KO^ liver pathogenesis, we decided to characterize their immune profile at the early stage of cholangiohepatitis development (50-week-old). To do so, we isolated the immune fraction from liver homogenates from 14-week-old (young) and 50-week-old (aged) *Oma1*^WT^ and *Oma1*^KO^ mice and classified their immune populations by multiparametric spectral flow cytometry using an extensive panel of 18 surface markers representing the most common immune subsets (Fig. EV5A). Based on total CD45^+^ cells, we performed a dimensional reduction followed by clusterization analysis and obtained 15 different immune clusters (Fig. 6A).

Remarkably, there was a massive phenotype of T cell exhaustion in *Oma1*^KO^ mice (Fig. 6A), at the early onset of *Oma1*^KO^ cholangiohepatitis. Both CD8^+^ and CD4^+^ T cells revealed a robust shift towards an activated state, and, within this enlarged population, most cells had become exhausted, as revealed by increased expression of PD-1 and Tim-3 exhaustion markers.

This was a progressive phenotype that aggravated over time. In the case of CD8^+^ T cells, even young *Oma1*^KO^ mice presented a decrease in the percentage of naive and central memory T (TCM) cells and an increase in effector memory T (TEM) cells (Fig. 6B), already showing enhanced exhaustion (Fig. 6B). Upon aging, these tendencies became even clearer (Fig. 6B). In fact, *Oma1*^KO^ aged livers reached a fourfold increase in the percentage of PD-1^+^ Tim-3^+^ CD8^+^ TEM cells, showing a severe exhausted phenotype, possibly suggestive of a diminished tumor-killing effector function.

In the case of CD4^+^ T cells, the differences in population shift were only evident upon aging, suggesting a progressive phenotype that is delayed from the CD8^+^ T-cell dysregulation. The evolution in the CD4^+^ T-cell population in the *Oma1*^KO^ mice consisted of a progressive decrease in the percentage of naive cells and an enrichment in TEM cells, which increasingly became more exhausted (Fig. 6C). In aged *Oma1*^KO^ mice, a tenfold increase in the percentage of PD-1^+^ Tim-3^+^ CD4^+^ TEM cells was estimated. In conclusion, there is an unusual progressive immune exhaustion phenotype during aging in *Oma1*^KO^ mice that likely contributes to tumor development (Fig. 6D).

To determine whether OMA1 absence in immune cells can intrinsically promote T cell exhaustion, we performed *Oma1*^WT^ and *Oma1*^KO^ bone marrow transplantation experiments. To do so, we lethally irradiated young *Oma1*^WT^ and *Oma1*^KO^ mice and transferred half of the mice from each genotype with bone marrow that either expressed or lacked OMA1 (Fig. EV5B,C). Twelve weeks after the irradiation, mice were euthanized, and their liver and blood immune profiles were analyzed.

Plasma transaminases were abnormally elevated in all irradiated mice (Fig. EV5D) (i.e., ALT-GPT was 80% higher and AST-GOT 51% compared to non-irradiated 10-week-old WT mice); however, when analyzed exclusively by their recipient genotype at 30 weeks after irradiation, *Oma1*^KO^ mice presented elevated AST-GOT values compared to *Oma1*^WT^ animals (Fig. EV5E). In addition, plasma parameters evidencing liver dysfunction were lower in *Oma1*^KO^ recipient mice, independently of their bone marrow genotype (Fig. EV5F). Direct and total bilirubin tended to be increased in *Oma1*^KO^ recipient mice (Fig. EV5G), but it should be noted that all irradiated mice presented abnormally low levels of these parameters, too. Overall, *Oma1*^KO^ liver damage was shown to be dependent on the recipient’s genotype, rather than the genotype of the bone marrow.

Liver immune profiling from irradiated *Oma1*^WT^ and *Oma1*^KO^ mice transferred with OMA1-expressing or OMA1-lacking bone marrow showed a tendency towards increasing CD8^+^ T cell exhaustion depending on the recipient’s genotype. Naive CD8^+^ T cells were remarkably affected by OMA1 expression in the bone marrow, being reduced in mice transplanted with *Oma1*^KO^ bone marrow (Fig. EV5H). However, TEM CD8^+^ cells were elevated in *Oma1*^KO^ livers independently of their bone marrow’s genotype (Fig. EV5H). PD-1-expressing CD8^+^ and TEM CD8^+^ cells were also augmented in *Oma1*^KO^ recipient mice (Fig. EV5I), showing that the exhaustion enhancement is not an intrinsic characteristic of *Oma1*^KO^ CD8^+^ T cells, but rather determined by the absence of OMA1 in the liver parenchyma. These data suggest that the liver *Oma1*^KO^ immune phenotype is not intrinsic to the immune system but rather a consequence of enhanced immunosurveillance and immunogenicity of the liver.

### Increased immunogenicity in *Oma1*^KO^ hepatocytes

**Figure 7:**
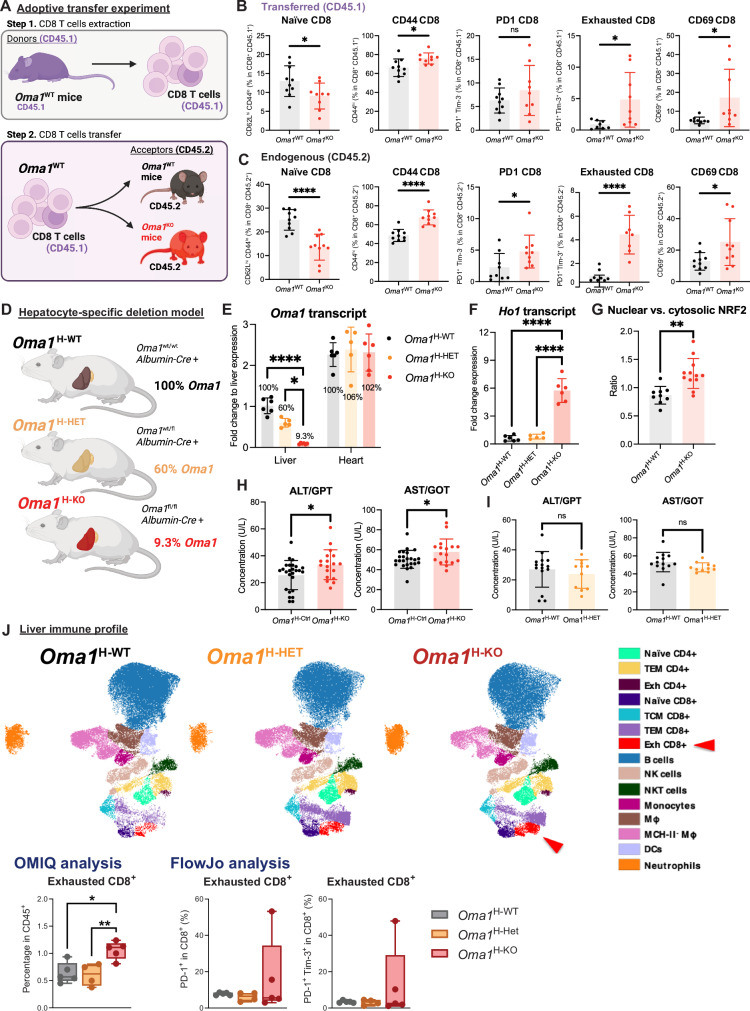
*Oma1* deletion in hepatocytes is sufficient to promote immune exhaustion. (**A**) Experimental design scheme for adoptive transfer. Briefly, CD45.1 CD8^+^ T cells were extracted from 16-week-old male *Oma1*^WT^ CD45.1 mice (*n* = 20) using their spleen and ganglia. Extracted CD45.1 CD8^+^ T cells were further transferred to 15-week-old male *Oma1*^WT^ (*n* = 10) and *Oma1*^KO^ (*n* = 10) CD45.2 mice. (**B**) Activation and exhaustion profile of *Oma1*^WT^ transferred liver CD8^+^ T cells (CD45.1) in *Oma1*^WT^ (*n* = 10) and *Oma1*^KO^ (*n* = 10) mice, including percentage of naive CD8^+^ T cells (*P* = 0.0373), total CD44^+^ CD8^+^ T cells (*P* = 0.0144), PD-1^+^ CD8^+^ T cells (*P* = 0.2747), PD-1^+^ Tim-3^+^ CD8^+^ T cells (*P* = 0.0228) and CD69^+^ CD8^+^ T cells (*P* = 0.0257). *P* values from two-tailed Student’s *t* tests. (**C**) Activation and exhaustion profile of endogenous liver CD8^+^ T cells (CD45.2) in *Oma1*^WT^ (*n* = 10) and *Oma1*^KO^ (*n* = 10) mice, including percentage of naive CD8^+^ T cells (*P* < 0.0001), total CD44^+^ CD8^+^ T cells (*P* < 0.0001), PD-1^+^ CD8^+^ T cells (*P* = 0.0410), PD-1^+^ Tim-3^+^ CD8^+^ T cells (*P* < 0.0001) and CD69^+^ CD8^+^ T cells (*P* = 0.0332). All datasets except “CD69%CD8 CD45.1”, “PD1%CD8 CD45.2” and “PD1 Tim3%CD8 CD45.2” passed normality. When normality was not passed, a Mann–Whitney test was used to obtain the *P* value. When normality was passed, a two-tailed Student’s *t* test was used to obtain the *P* value. ns *P* > 0.05, ^∗^*P* < 0.05, ^∗∗^*P* < 0.01, ^∗∗∗^*P* < 0.001, and ^∗∗∗∗^*P* < 0.0001. (**D**) Hepatocyte-specific *Oma1* deletion model scheme, depicting hepatocyte-specific *Oma1*^WT^ and *Oma1*^KO^ animals expressing Albumin-Cre and presenting *Oma1*^wt/wt^ or *Oma1*^fl/fl^ gene. (**E**) Relative *Oma1* transcript in the liver and heart from 10-week-old hepatocyte-specific *Oma1-*expressing (*Oma1*^H-WT^ *n* = 6 and *Oma1*^H-HET^ *n* = 5) or *-*deleted animals (*Oma1*^H-KO^ *n* = 6). Liver: *Oma1*^H-KO^ vs. *Oma1*^H-WT^ *P* < 0.0001, *Oma1*^H-KO^ vs. *Oma1*^H-HET^ *P* = 0.0276. Two-way ANOVA, Tukey’s multiple comparisons test. (**F**) Relative *Ho1* transcript in the liver from 10-week-old hepatocyte-specific male *Oma1-*expressing (*Oma1*^H-WT^ *n* = 6 and *Oma1*^H-HET^ *n* = 5) or *-*deleted animals (*Oma1*^H-KO^ *n* = 6). *Oma1*^H-KO^ vs. *Oma1*^H-WT^ *P* < 0.0001, *Oma1*^H-KO^ vs. *Oma1*^H-HET^ *P* < 0.0001. *“Ho1* transcript” values passed normality. One-way ANOVA, Tukey’s multiple comparisons test. (**G**) Nuclear vs. cytosolic NRF2 quantification (*P* = 0.0026) from 10-week-old male hepatocyte-specific *Oma1-*expressing (*Oma1*^H-WT^ *n* = 10) or *-*deleted animals (*Oma1*^H-KO^ *n* = 10). Values passed normality. Unpaired *t* test. (**H**) Plasma ALT-GPT (*P* = 0.0324) and AST-GOT (*P* = 0.0364) levels from 10-week-old male hepatocyte-specific *Oma1-*expressing (*Oma1*^H-WT^ *n* = 15 and *Oma1*^H-HET^ *n* = 12) or *-*deleted animals (*Oma1*^H-KO^ *n* = 19). “ALT-GPT” values did not pass normality (Mann–Whitney test), while “AST-GOT” values did (unpaired *t* test). (**I**) Plasma ALT-GPT and AST-GOT levels from 10-week-old male hepatocyte-specific *Oma1-*expressing animals, comparing between *Oma1*^H-WT^ *n* = 15 and *Oma1*^H-HET^ *n* = 12. “ALT-GPT” values did not pass normality, while “AST-GOT” values did. *P* value from unpaired two-tailed Student’s *t* test (when data passed normality) or Mann–Whitney test (when data did not pass normality) is represented in each graph. (**J**) Representative UMAP plots of liver CD45^+^ populations in >50 weeks-old mice from male hepatocyte-specific *Oma1-*expressing (*Oma1*^H-WT^ *n* = 5 and *Oma1*^H-HET^ *n* = 4) or *-*deleted animals (*Oma1*^H-KO^ *n* = 5) analyzed by OMIQ using 17 markers. Analysis of exhausted CD8+ populations both by OMIQ (WT vs. KO *P* = 0.0109, HET vs. KO *P* = 0.0096) and by FlowJo analysis. Boxes represent the 25th to 75th percentiles, with the median in the center and whiskers extending to the minimum and maximum data points. ns *P* > 0.05, ^∗^*P* < 0.05, ^∗∗^*P* < 0.01, ^∗∗∗^*P* < 0.001, and ^∗∗∗∗^*P* < 0.0001. Source data are available online for this figure.

To determine whether the increased CD8^+^ T cell activation stems from abnormally increased parenchymal immunogenicity in *Oma1*^KO^ livers, we transferred *Oma1*^WT^ CD45.1 CD8^+^ T cells to 15-week-old CD45.2 *Oma1*^WT^ or *Oma1*^KO^ mice (Fig. 7A) and analyzed both their transferred and endogenous liver CD8^+^ T cell populations after 16 days.

**Figure EV6:**
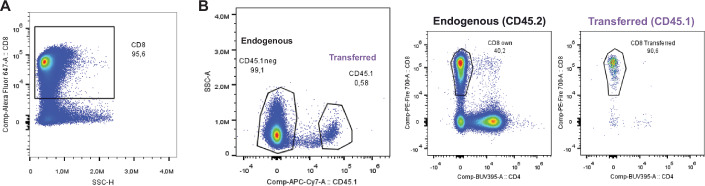
Analysis of transferred and endogenous liver CD8^+^ T cells populations. (**A**) CD8^+^ cells content in CD45.1 CD8^+^ T cells extracts used for adoptive transfer. (**B**) Gating strategy to analyze separately CD45.2 (endogenous) from CD45.1 (transferred) liver CD8^+^ T cells after 16 days of the adoptive transfer experiment. Cells were gated separately according to their CD45.1 level of expression (left). CD8^+^ vs. CD4^+^ expression pattern in CD45.1-negative cells (endogenous) and CD45.1-expressing cells (transferred) (right).

Transferred *Oma1*^WT^ CD45.1 CD8^+^ T cells extracts were 95.6% pure (Fig. EV6A) and 16 days after the adoptive transfer, liver CD8^+^ T cells consisted of both endogenous (CD45.2) and transferred (CD45.1) CD8^+^ T cells (Fig. EV6B). Transferred *Oma1*^WT^ CD8^+^ T cells became more activated and exhausted in *Oma1*^KO^ livers (Fig. 7B) and their profile phenocopied the endogenous CD8^+^ T-cell pattern (Fig. 7C), demonstrating that the absence of OMA1 in the liver parenchyma increases its immunogenicity.

**Figure EV7:**
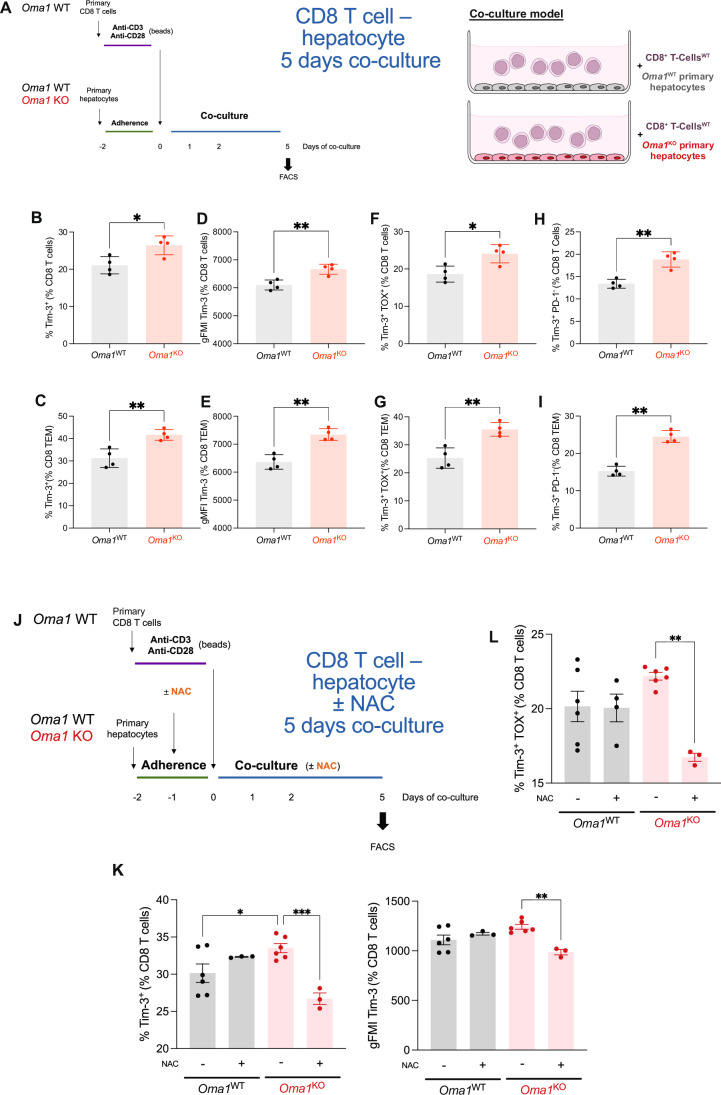
Assessment of immunogenicity of *Oma1*^KO^ hepatocytes. (**A**) Co-culture model: primary hepatocytes were isolated from 26-week-old male *Oma1*^WT^ (*n* = 2) and *Oma1*^KO^ (*n* = 2) mice and cultured for 48 h before adding naive or pre-activated (bead) CD8^+^ T cells. Exhaustion markers were measured after 5 days in co-culture. Technical replicates for (**B**–**I**) are: *Oma1*^WT^ (*n* = 4) and *Oma1*^KO^ (*n* = 4). (**B**, **C**) Overall prevalence of Tim‑3 expression within total CD8^+^ T cells (*P* = 0.0209) (**B**) or within the effector‑memory (TEM) CD8^+^ T cells (*P* = 0.0083) (**C**) (mean ± SD). (**D**, **E**) Same as (**B**, **C**) but assessed by geometric Mean Fluorescence Intensity (gMFI) (in total CD8^+^ T cells *P* = 0.0043, in TEM CD8 + T cells *P* = 0.0012) (mean ± SD). (**F**, **G**) Activated CD8^+^ T cell subpopulations (in total CD8^+^ T cells *P* = 0.0159, in TEM CD8 + T cells *P* = 0.0049) (mean ± SD). (**H**, **I**) Committed to exhaustion CD8^+^ T cell subpopulations (in total CD8^+^ T cells *P* = 0.0030, in TEM CD8 + T cells *P* = 0.0001). (**J**) Co-culture model: primary hepatocytes were isolated from 26-week-old male Oma1^WT^ (*n* = 2) and Oma1^KO^ (*n *= 2) mice and cultured for 5 days in the presence of the antioxidant NAC before adding naive CD8^+^ T cells. Technical replicates for (**K**, **L**) were: *Oma1*^WT^ – NAC (*n* = 6), *Oma1*^WT^ + NAC (*n* = 4), *Oma1*^KO^ – NAC (*n* = 6), *Oma1*^KO^ + NAC (*n* = 3). (**K**) Overall prevalence of Tim‑3 expression within total CD8^+^ T cells in the presence or absence of NAC (%Tim-3: KO – vs. + NAC *P *= 0.0024, NAC WT vs. KO *P* = 0.0255; gFMI Tim-3: KO – vs. + NAC *P* = 0.0038) (mean ± SD). (**L**) Committed to exhaustion CD8^+^ T cell subpopulations in the presence of absence of NAC (*P* = 0.0026). *P* value from ordinary one-way ANOVA test post-hoc Multiple comparisons Sidak is represented in each graph (mean ± SD). ns *P* > 0.05, *P < 0.05, **P < 0.01, ***P < 0.001, and ****P < 0.0001.

To further isolate the effect of hepatocytes in the exhaustion of CD8^+^ T cells, we co-cultured *Oma1*^WT^ and *Oma1*^KO^ primary hepatocytes (isolated from 26 weeks-old male mice) with *Oma1*^WT^ CD8^+^ T cells and analyzed exhaustion markers in the immune cells (Fig. EV7A). Interestingly, co-culture with *Oma1*^KO^ hepatocytes led to an increase in the population of CD8^+^ T cells expressing the exhaustion marker (Tim-3^+^) with respect to the total CD8^+^ T population (Fig. EV7B) and with respect to the effector‑memory CD8^+^ T cells (TEM) (Fig. EV7C). This was confirmed by measuring the geometric Mean Fluorescence Intensity (gMFI) of Tim‑3 in both populations (Fig. EV7D,E). Interestingly, these Tim-3^+^ CD8^+^ T cell subsets co-expressed other characteristic exhaustion markers, such as TOX (Fig. EV7F,G) but not PD-1 (Fig. EV7H,I), suggesting a context of late exhaustion. In summary, *Oma1*^KO^ hepatocytes are sufficient to promote CD8^+^ T-cell overactivation and exhaustion.

We postulated that mitochondrial ROS-mediated NRF2 signaling in *Oma1*^KO^ hepatocytes leads to increased immunogenicity. We took advantage of this ex vivo system to assess whether controlling ROS in *Oma1*^KO^ hepatocytes could impact their immunogenicity. Before their co-culture, *Oma1*^WT^ and *Oma1*^KO^ primary hepatocytes were treated with N-acetylcysteine (NAC) (Fig. EV7J), which should blunt NRF2 signaling by scavenging reactive oxygen species. NAC treatment abolished the hepatocyte-promoted, *Oma1*^KO^-dependent, CD8^+^ T cell exhaustion induction, while not affecting CD8^+^ T cells co-cultured with *Oma1*^WT^ hepatocytes (Fig. EV7L,K). This supports our model that ROS production in *Oma1*^KO^ hepatocytes causes persistent NRF2 signaling, triggering immunogenicity and promoting CD8^+^ T cells exhaustion. Taken together, these data suggest that hepatic *Oma1* expression is crucial to shape the immune response in the liver, acting as an essential link between early liver dysfunction and the subsequent increased tumorigenesis observed during *Oma1*^KO^ aging.

**Figure EV8:**
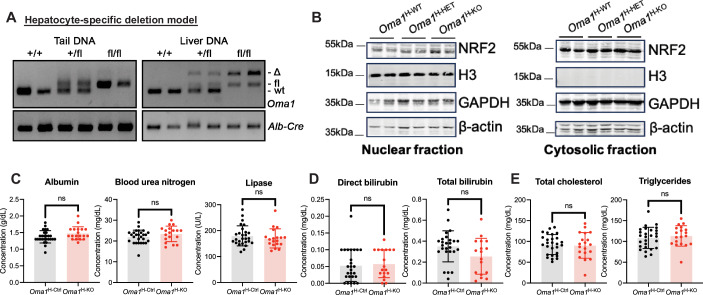
Hepatocyte-restricted ablation of Oma1. (**A**) *Oma1* genotype in tail and liver samples from 10 weeks-old male hepatocyte-specific *Oma1-*expressing (*Oma1*^H-WT^ *n* = 6 and *Oma1*^H-HET^ *n* = 5) or *-*deleted animals (*Oma1*^H-KO^ *n* = 6). (**B**) Representative Western Blot from 10-week-old male hepatocyte-specific *Oma1-*expressing (*Oma1*^H-WT^ *n* = 10) or *-*deleted animals (*Oma1*^H-KO^ *n* = 10) nuclear and cytosolic liver fractions. (**C**) Plasma albumin, blood urea nitrogen, and lipase values from 10-week-old male hepatocyte-specific *Oma1-*expressing (*Oma1*^H-WT^ *n* = 15 and *Oma1*^H-HET^ *n* = 12) or *-*deleted animals (*Oma1*^H-KO^ *n* = 19) (mean ± SD). “Albumin” values did not pass normality, while “BUN” and “Lipase” data passed normality. (**D**) Plasma direct and total bilirubin values from 10-week-old male hepatocyte-specific *Oma1-*expressing (*Oma1*^H-WT^ *n* = 15 and *Oma1*^H-HET^ *n* = 12) or *-*deleted animals (*Oma1*^H-KO^ *n* = 19) (mean ± SD). Values did not pass normality. (**E**) Plasma total cholesterol and triglycerides values from 10-week-old male hepatocyte-specific *Oma1-*expressing (*Oma1*^H-WT^ *n* = 15 and *Oma1*^H-HET^ *n* = 12) or *-*deleted animals (*Oma1*^H-KO^ *n* = 19) (mean ± SD). Values passed normality. *P* value from unpaired two-tailed Student’s *t* test (when data passed normality) is represented in each graph. ns *P* > 0.05, ^∗^*P* < 0.05, ^∗∗^*P* < 0.01, ^∗∗∗^*P* < 0.001, and ^∗∗∗∗^*P* < 0.0001.

Finally, to confirm that the loss of OMA1 in hepatocytes gives rise to *Oma1*^KO^ liver damage and enhanced immunogenicity, we generated and analyzed hepatocyte-specific *Oma1*-deleted mice (Figs. 7D and EV8A) (Albumin-Cre, Oma1^fl/fl^ animals (*Oma1*^H-KO^) vs. Albumin-Cre, Oma1^wt/wt^ (*Oma1*^H-WT^) and Oma1^wt/fl^ animals (*Oma1*^H-HET^)). *Oma1* transcript levels in *Oma1*^H-KO^ livers dropped to 9.3%, while in *Oma1*^H-HET^ livers they fell to 60%, with no changes in other tissues like the heart (Fig. 7E). Exclusively, *Oma1*^H-KO^ mice showed a dramatic enrichment of Nrf2-dependent *Ho1* transcription (Fig. 7F) and, accordingly, abnormal NRF2 translocation to the nucleus (Figs. 7G and EV8A,B). Plasma ALT-GPT and AST-GOT values were abnormally elevated in *Oma1*^H-KO^ mice (Fig. 7H), while *Oma1*^H-HET^ showed no difference compared to *Oma1*^H-WT^ animals (Fig. 7I), implying that their remaining 60% *Oma1*-expression is sufficient to protect hepatocytes from death. Other liver dysfunction parameters that typically change in constitutive *Oma1*^KO^ mice were not altered at this age in the hepatocyte-specific KO animals (Fig. EV8C–E), suggesting that other cell types could be contributing to this phenotype or that the phenotypic manifestation in hepatocyte-specific *Oma1*-depleted animals might be delayed with respect to the full *Oma1*^KO^ model.

Remarkably, hepatocyte-specific *Oma1*^H-KO^ aged livers ( > 50 weeks old) showed a sharp increase in CD8^+^ T cell exhaustion (Fig. 7J), confirming that hepatocyte-specific *Oma1* deletion is sufficient to boost their immunogenicity and drive immune exhaustion. In conclusion, our results evidence that the origin of *Oma1*^KO^ liver disease and immune modulation primarily comes from the liver parenchyma, instead of an immune-intrinsic defect. More specifically, *Oma1* deletion in hepatocytes is sufficient to initiate Nrf2-dependent oxidative stress and liver damage, abnormally activating and exhausting CD8^+^ T cells. Thus, by controlling hepatic immunogenicity, *OMA1* expression in hepatocytes acts as a brake against liver injury and tumorigenesis.

### Genetic background impact on OMA1-dependent phenotype

**Figure EV9:**
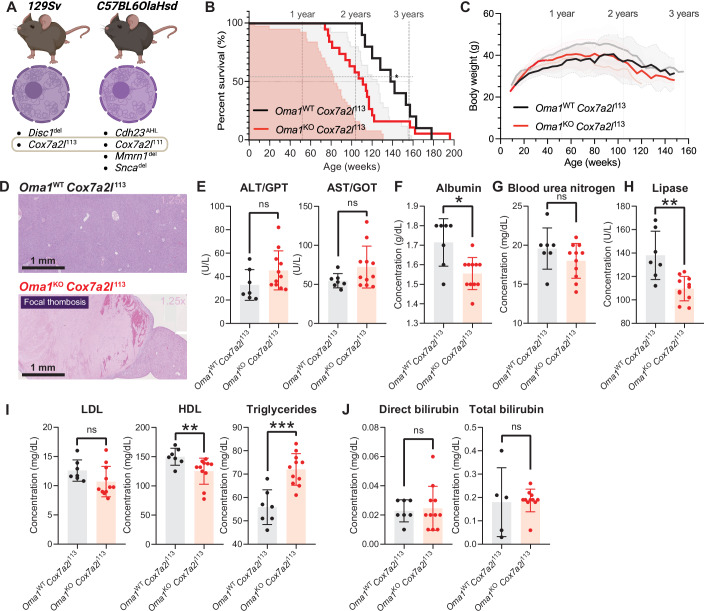
Genetic background impact on OMA1-dependent CLD phenotype. (**A**) Reported genetic background divergence between 129 Sv and C57BL6OlaHsd mice, including alterations in *Disc1*, *Cox7a2l*, *Cdh23*, *Mmrn1*, and *Snca*. (**B**) Survival analysis (*P* = 0.0151) of male *Oma1*^WT^ *Cox7a2l*^113^ (*n* = 10) and *Oma1*^KO^ *Cox7a2l*^113^ (*n* = 19) mice in an inbred C57BL6/JOlaHsd background. *P* value from Gehan–Breslow–Wilcoxon test is represented in the graph. (**C**) Body weight evolution of male *Oma1*^WT^ *Cox7a2l*^113^ (*n* = 10) and *Oma1*^KO^ *Cox7a2l*^113^ (*n* = 19) mice in an inbred C57BL6/JOlaHsd background. *P* value from a Mixed-effects model testing the “OMA1 expression” fixed effect is represented in the graph. (**D**). H&E histological analysis from a male *Oma1*^KO^ mouse autopsy presenting focal liver thrombosis at 78 weeks of age, compared to a representative 86-week-old *Oma1*^WT^ liver. (**E**) Plasma ALT-GPT, AST-GOT from 15-week-old male *Oma1*^WT^ *Cox7a2l*^113^ (*n* = 7) and *Oma1*^KO^ *Cox7a2l*^113^ (*n* = 11) mice fasted for 6 h. “ALT-GPT” and “AST-GOT” values passed normality (mean ± SD). (**F**) Plasma albumin (*P* = 0.0108) from 15-week-old male *Oma1*^WT^ *Cox7a2l*^113^ (*n* = 7) and *Oma1*^KO^ *Cox7a2l*^113^ (*n* = 11) mice fasted for 6 h. “Albumin” values did not pass normality (mean ± SD). (**G**) Plasma BUN from 15-week-old male *Oma1*^WT^ *Cox7a2l*^113^ (*n* = 7) and *Oma1*^KO^ *Cox7a2l*^113^ (*n* = 11) mice fasted for 6 h. “BUN” values passed normality (mean ± SD). (**H**) Plasma Lipase activity (*P* = 0.0013) from 15-week-old male *Oma1*^WT^ *Cox7a2l*^113^ (*n* = 7) and *Oma1*^KO^ *Cox7a2l*^113^ (*n* = 11) mice fasted for 6 h (mean ± SD). (**I**) Plasma LDL, HDL (*P* = 0.0052), and TAGs (*P* = 0.0002) in 15-week-old *Oma1*^WT^ *Cox7a2l*^113^ (*n* = 7) and *Oma1*^KO^ *Cox7a2l*^113^ (*n* = 11) mice fasted for 6 h. “LDL” and “TAGs” values passed normality, while “HDL” and “Total cholesterol” did not (mean ± SD). (**J**) Plasma direct bilirubin and total bilirubin from 15-week-old male *Oma1*^WT^ *Cox7a2l*^113^ (*n* = 7) and *Oma1*^KO^ *Cox7a2l*^113^ (*n* = 11) mice fasted for 6 h. “Direct bilirubin” and “Total bilirubin” values did not pass normality. *P* value from unpaired two-tailed Student’s *t* test (when data passed normality) or Mann–Whitney test (when data did not pass normality) is represented in each graph (mean ± SD). ns *P* > 0.05, ^∗^*P* < 0.05, ^∗∗^*P* < 0.01, ^∗∗∗^*P* < 0.001, and ^∗∗∗∗^*P* < 0.0001.

When constitutive *Oma1*^KO^ mice were generated by Quirós and colleagues (Quiros et al, 2012), we reported—in contrast to our current results (Fig. 2A)—that the lifespan of *Oma1*^KO^ mice was unaffected compared to control animals. An experimental difference between the *Oma1*^KO^ animals analyzed here and those from Quirós et al is their genetic background. The animals in the current study are full inbred C57BL/6JOlaHsd background (more than 20 generations of backcrosses), whereas animals from the 2012 study harbored a mixed 129Sv/C57BL/6J background. Genetic divergence between 129Sv and C57BL/6JOlaHsd backgrounds has been previously characterized (Enríquez, 2019). Some of the genetic differences between them rely on *Disc1*, *Cox7a2l*, *Cdh23*, *Mmrn1*, and *Snca* (Fig. EV9A).

To test whether the disparity in survival could come from the difference in genetic backgrounds, we analyzed the lifespan of *Oma1*^KO^ mice in our inbred C57BL/6JOlaHsd background, presenting a 129 Sv background-specific genetic difference: *Cox7a2l*^113^. Very interestingly, *Cox7a2l*^113^ is sufficient to extend *Oma1*^KO^ median survival (Fig. EV9B), even if it is still significantly reduced compared to their *Oma1*^WT^ controls. In line with this, *Oma1*^KO^ *Cox7a2l*^113^ mice do not present age-associated body weight loss, which correlates with their improved survival (compare Figs. EV9C and  1A). Nonetheless, autopsies also revealed liver pathology in *Oma1*^KO^ *Cox7a2l*^113^ mice at 80 weeks of age (Fig. EV9A), implying that *Cox7a2l*^113^ contributes to the *Oma1*-dependent CLD progression and severity but does not totally prevent it.

Furthermore, plasma analysis of 15-week-old *Oma1*^KO^ *Cox7a2l*^113^ mice showed very similar early liver alterations to those found in *Oma1*^KO^ *Cox7a2l*^111^ mice, including transaminases rise tendency (Fig. EV9B), albumin drop (Fig. EV9C), and other known alterations (Fig. EV9D–F). Exceptionally, bilirubin levels were fully recovered in *Oma1*^KO^ *Cox7a2l*^113^ mice (Fig. EV9G). The potential relevance of the additional genetic differences affecting *Disc1*, *Cdh23*, *Mmrn1*, or *Snca* was not evaluated. Nevertheless, this analysis strongly demonstrates the relevance of the genetic background on the debut and progression of CLD caused by *Oma1* ablation.

## Discussion

The results described in this study show that *Oma1* expression in hepatocytes acts as a modulator of liver metabolism and hepatic immunogenicity, preventing chronic inflammation, immune exhaustion, and liver tumorigenesis. Remarkably, this long-lasting study has been performed on physiologically aged animals, which provides invaluable new mechanistic insights into CLD initiation and progression.

Modeling CLD in rodents is challenging (Delire et al, 2015; Nevzorova et al, 2020). Up to now, there are no murine models that recapitulate CLD as a global entity, exhibiting all its relevant metabolic, immunologic, and specific hepatic alterations in an age-dependent manner. Age has been shown to be a determinant factor in human CLD progression and cirrhosis onset (Pinzani et al, 2001; Pradat et al, 2007; Kim et al, 2015). In fact, it has been argued that regardless the age of infection, untreated hepatitis C virus (HCV) patients are expected to develop cirrhosis at about 65 years of age (Pradat et al, 2007). Therefore, studying CLD progression with age in mice, as our study proposes, might represent the most accurate way to obtain relevant results for human CLD pathology.

We postulate that the mechanism behind OMA1 ablation-induced hepatic inflammation is based on the triggering of hepatic oxidative stress and early hepatocyte death. KEAP1-Nrf2 pathway activation has been demonstrated to be protective upon liver damage in murine and human data (Hammoutene et al, 2023; Seedorf et al, 2023). In young *Oma1*^KO^ mice, this pathway is persistently overactivated as a protective strategy against oxidative stress, as it is known to happen in human CLD patients (Cheng et al, 2015). Interestingly, it has been shown that persistent NRF2 activation can become insufficient or even negative upon chronicity (Lee et al, 2014; Umemura et al, 2016; Gan et al, 2024), in line with our murine observations. Oxidative stress is a well-known initiator of hepatic inflammation (Cichoz-Lach and Michalak, 2014). Hepatocytes that accumulate lipids become vulnerable to oxidative stress, which stimulates the production of DAMPs (Del Campo et al, 2018). These molecules are sensed by Kupffer cells and activate hepatocyte stellate cells (HSCs), which promote a pro-fibrogenic inflammatory phenotype. We have shown that OMA1 in hepatocytes is required to restrict oxidative stress, protecting against hepatic chronic inflammation and fibrosis.

The relationship between OMA1 and ROS is highly context-dependent; while OMA1 deficiency elevates ROS in yeast (Bohovych et al, 2016), several mammalian models report unchanged or decreased baseline levels (Wu et al, 2021; Chen et al, 2024). Although we have shown that NAC treatment prevents CD8^+^ T cell overactivation in our *Oma1*^KO^ hepatocyte co-cultures, these findings must be interpreted with caution. The cellular effects of NAC can be complex and stochastic rather than purely antioxidant-specific. Furthermore, hydrogen peroxide is typically a poor direct inducer of NRF2 due to its low reactivity with Keap1, often preferentially activating alternative pathways like NF-kb (Ishii et al, 2022). Thus, while our data implicate oxidative pathways in hepatic immunogenicity, the precise mechanistic link between OMA1, ROS, and NRF2 requires further analysis to rule out indirect signaling crosstalk.

Recently, it was shown that electron flow preference has a direct effect on cell immunogenicity (Mangalhara et al, 2023). Favoring mitochondrial complex I-mediated electron flow elevates succinate levels which, consequently, modulate epigenetically MHC-I expression and antigen presentation and processing genes (Mangalhara et al, 2023). Here, we show how *Oma1*^KO^ liver parenchyma, whose mitochondria oxidize succinate less efficiently, is in fact more immunogenic. Initially, this immunogenicity increase can promote an early activation of CD8^+^ T cells, but chronically, it results in massive T cell exhaustion and immunosurveillance impairment during aging. We believe this is the main mechanism behind liver tumorigenesis regulation by mitochondrial OMA1 expression.

The metalloprotease OMA1 exerts its main actions through the proteolysis of OPA1 (Ehses et al, 2009; Head et al, 2009; Baker et al, 2014) and DELE1 (Guo et al, 2020; Fessler et al, 2020). Interestingly, the *Oma1*-*Dele1*-mediated ISR axis has been shown to protect against ferroptosis in mice suffering from mitochondrial cardiomyopathy. Thus, ablation of *Oma1* worsens their cardiac phenotype (Ahola et al, 2022). Here, we have found no difference in the ATF4-dependent transcriptional program at any disease stage. Nonetheless, it is possible that an increased susceptibility to ferroptosis in the absence of OMA1 is indeed explaining the increased hepatocyte death found in young and aged *Oma1*^KO^ mice. OMA1-mediated iron homeostasis dysregulation might be playing a major role in the initiation and progression of CLD in this study.

The downstream target of OMA1, OPA1, has recently been linked to cancer (Herkenne et al, 2020; Zamberlan et al, 2022; Noguchi et al, 2023). Worse breast cancer prognosis and increased invasiveness have been observed with *Opa1* overexpression (Zamberlan et al, 2022). Accordingly, OPA1 silencing or specific pharmacological inhibition of its GTPase activity has been shown to reduce breast cancer proliferation and invasion, becoming a new promising target in cancer therapy. Given that OMA1 proteolyzes OPA1, *Opa1* overexpression has traditionally been equated to *Oma1* deletion, resonating with our data. Altogether, we believe that it is through the OMA1-OPA1 axis that tumor progression is regulated.

Nonetheless, we demonstrated that *Oma1* deletion does not intrinsically provide a growth advantage to tumor cells, as both the allograft and the chemically induced models revealed. On the contrary, another study showed that the absence of *Oma1* protected mice from colorectal cancer development (Wu et al, 2021), reinforcing that OMA1's relation to cancer might not be univocal, but rather complex and context-dependent.

The impact of the mice genetic background on our described phenotype is yet another evidence of this complex relationship. Phenotypic alterations coming from genetic background divergence is not a new concept (Doetschman, 2009). An illustrative example of this discordance can in fact be found in a closely related genetic model: the *Opa1*^tg^ mice model (Varanita et al, 2015). *Opa1*^tg^ mice generated in a C57BL/6 phenotype displayed normal survival compared to control mice. Conversely, *Opa1*^tg^ mice generated in a 129 Sv background presented increased mortality and tumor incidence. Modifier genes affecting the phenotype of a mutant gene can make the difference among genetic backgrounds (Doetschman, 2009). In our case study, *hybrid vigor* from the previously generated *Oma1*^KO^ mice (Quiros et al, 2012), which presented a mixed 129 Sv/C57BL/6 J background, could have hidden or ameliorated their phenotype (Birchler et al, 2006; GUSTAFSSON, 1946).

This variability in expression of the here described mouse phenotype may put into question its relevance to human pathophysiology. However, in humans, low expression of *OMA1* in breast, colorectal, and lung cancer is associated with poorer prognosis (Human Protein Atlas). In this line, silencing *OMA1* in patient-derived metastatic breast cancer cells significantly increased their proliferative and migratory properties and enhanced their epithelium to mesenchymal transition (Daverey et al, 2019), which is associated with increased tumor invasiveness. In any case, the fact that *OMA1* expression in humans inversely correlates with liver inflammation severity, which is reproduced in our experiments, stresses the relevance of our work for human pathology.

The protection that OMA1 ablation confers against heart failure (Acin-Perez et al, 2018) and neurodegenerative disease (Korwitz et al, 2016) has raised a significant interest in developing OMA1 inhibitors (Tobacyk et al, 2019; Houston et al, 2021; Alavi, 2021, 2022). We believe that our results do not compromise the suitability of OMA1 as a qualified pharmacological target of clinical interest. First, the selective inhibition activity of any compound will never recapitulate the loss of function achieved by constitutive genetic deletion. Constitutive genetic deletions lead to a total absence of a protein in all tissues during all developmental stages, including embryonic development, whereas protein inhibition does not affect protein presence (with few exceptions (Long et al, 2012; Burslem et al, 2018)) but its activity, which would never be absolutely ablated. Therefore, a constitutive genetic ablation model represents the most extreme effects potentially coming from the absence of a target protein. Drugs of broad clinical use demonstrated severe adverse effects in preclinical studies based on genetic ablation. One representative example is the widespread clinical use of statins (Trialists, 2005) (i.e., 3-hydroxy-3-methylglutaryl-coenzyme A reductase (HMGCR) inhibitors). Constitutive *Hmgcr*^KO^ mice die at an early stage of development (Ohashi et al, 2003) (i.e., before E8.5), and those mice whose genetic deletion is restricted to the liver present a median survival of 35 days (Nagashima et al, 2012). Nonetheless, adjusted doses in the chronic use of statins are considered safe and have proven to exhibit notorious benefits for patients (Trialists, 2005), even when statins are known to be potent inhibitors, binding to HMGCR with 10,000-fold higher affinity than its substrate (Bansal, 2022).

Second, drug-induced liver injury (DILI) is common to many drugs of clinical use (Katarey and Verma, 2016). Paracetamol overdose is a paramount example, but many other commonly used drugs, such as antibiotics (Amoxicillin-clavulanate, Nitrofurantoin), anti-inflammatories (Diclofenac and Ibuprofen), immuno-suppressors (Azathioprine), anti-cancer (Flutamide), antipsychotics (Chlorpromazine), etc., may cause DILI (Katarey and Verma, 2016). DILI severity ranges from asymptomatic alterations of liver parameters to its most severe manifestation: acute liver failure (ALF). Remarkably, it was reported that near 60% of ALF cases in the United Kingdom are due to paracetamol overdose (Bernal and Wendon, 2013). Mitochondrial impairment is frequently involved in DILI (Mihajlovic and Vinken, 2022). Here, we show that complete ablation of *Oma1* from embryonic development may cause DILI with a relatively slow progression, but ALF has never been observed. Our study has established the maximum severity that chronic inhibition of OMA1 can cause in animal models, excluding a risk for ALF. We have shown that genetic context in mice and humans modulates the liver response to *Oma1* ablation, which opens the possibility to determine the beneficial non-toxic level of OMA1 inhibition according to the patient's basal *OMA1* and *OPA1* expression levels.

In conclusion, we believe that these results are crucial to understand the consequences of the deletion of OMA1 in its complexity and entail relevant implications both for human pathology and the upcoming OMA1-targeted therapies.

## Methods

**Table:** Reagents and tools table

Reagent/resource	Reference or source	Identifier or catalog number
**Experimental models**		
Mouse: C57BL6/JOlaHsd	Harlan Laboratories/Inotiv	C57BL/6JRccHsd
Mouse: *Oma1*^KO^	Quiros et al, 2012	N/A
Mouse: *Oma1*^fl/fl^	Wai et al, 2015	N/A
Mouse: Albumin-Cre	Postic et al, 1999	N/A
Mouse: *Cox7a2l*^113^	Cogliati et al, 2016	N/A
Mouse: *Oma1*^KO^ *Cox7a2l*^113^	This paper	N/A
Cell: *Oma1*^WT^ E0771	This paper	N/A
Cell: *Oma1*^KO^ E0771	This paper	N/A
**Antibodies**		
ATF4	Cell Signaling	Cat # 11815S
B220	Biolegend	Cat # 103287
Caspase-3 (IHC)	Cell Signaling	Cat # 9661
CD11b	Biolegend	Cat # 101222
CD11c	Tonbo Bioscience	Cat # 20-0114-U100
CD206	Biolegend	Cat # 141723
CD27	Biolegend	Cat # 124216
CD3	BD Biosciences	Cat # 562332
CD4	Tonbo Bioscience	Cat # 75-0042-U100
CD44	Tonbo Bioscience	Cat # 50-0441-U100
CD45	BD Biosciences	Cat # 550539
CD45.1	Tonbo Bioscience	Cat # 25-0453-U100
CD62L	Biolegend	Cat # 104445
CD69	Biolegend	Cat # 104505
CD8	Biolegend	Cat # 100791
F4/80	Biolegend	Cat # 123133
F4/80 (IHC)	Cell Signaling	Cat # 70076
GAPDH	Abcam	Cat #
Ghost Dye Violet 540	Tonbo Bioscience	Cat # 13-0879-T500
TOX	eBioscience	Cat # 50-6502-80
Histone-3 (H3)	Abcam	Cat # ab1791
Ki67 (IHC)	Thermo Fisher	Cat # PA5-19462
Ly6C	Biolegend	Cat # 128041
Ly6G	Biolegend	Cat # 127628
MHC-II	Biolegend	Cat # 107662
NK1.1	Biolegend	Cat # 156519
NRF2	Santa Cruz	Cat # sc-365949
PD-1	eBioscience	Cat # 46-9985-80
Phospho-eIF2α (P-eiF2α)	Cell Signaling	Cat # 119A11
TIM-3	BD Biosciences	Cat # 747618
β-Actin	Sigma	Cat # A2066
**Oligonucleotides and other sequence-based reagents**		
Mouse *Oma1* gRNA-1 for CRISPR;	This paper	5’CACCGAAGCTGTCCTGCCGTAATAG
Mouse *Oma1* gRNA-1 for CRISPR;	This paper	5’AAACCTATTACGGCAGGACAGCTTC
Mouse Oma1 gRNA-2 for CRISPR;	This paper	5’CACCGACCTGCCCACGATGATAGCA
Mouse Oma1 gRNA-2 for CRISPR;	This paper	5’AAACTGCTATCATCGTGGGCAGGTC
Scr. gRNA for CRISPR;	This paper	5’CACCGTCGTTGTTAGCCTTGCGGG
Scr. gRNA for CRISPR;	This paper	5’AAACCCCGCAAGGCTAACAACGAC
**Chemicals, enzymes, and other reagents**		
Hematoxylin	Sigma	HSS80
Eosin	Sigma	HT1103128
Picrosirius red (PSR)	Sigma	365548
3-Methylcholanthrene (3MC)	Sigma	213942
Bodipy-493/503	Thermo Fisher	D3922
**Software**		
Fiji		https://fiji.sc
Prism 9	GraphPad	https://www.graphpad.com
SDS 2.4	Applied Biosystems	N/A
Quant Studio v.1.4.3.	Applied Biosystems	N/A
Ingenuity Pathway Analysis (IPA)	Qiagen	N/A
NDP view2		
Fiji Macro to extract TIFF files from .czi	This paper	N/A
Fiji Macro to quantify %PSR	This paper	N/A
Fiji Macro to quantify %IHC+	This paper	N/A
OMIQ	Dotmatics	https://app.omiq.ai/login
**Other**		
Whole liver proteome from 10-week-old mouse	This paper	
Whole liver proteome from 90-week-old mouse	This paper	
RNAseq liver from 90-week-old mouse	This paper	

### Study design

1. Experimental Groups:
  - Control: C57BL/6JOlaHsd wild-type (WT) mice
  - Case: C57BL/6JOlaHsd Oma1 knockout (Oma1^KO^) mice
2. Assessment of Tumor Incidence and Survival:
  - Age-matched control vs case mice monitored for primary hepatic tumor incidence and overall survival rates over a defined period.
3. Evaluation of Hepatic Inflammation and Fibrosis:
  - Histopathological analysis of liver tissues from control vs case mice to assess inflammation, fibrosis, and liver injury markers.
  - Measurement of inflammatory cytokine levels in liver homogenates.
4. Characterization of Immune Cell Populations:
  - Flow cytometric analysis of CD8^+^ and CD4^+^ T cell populations in liver tissues from control vs case mice at different stages of tumor progression.
  - Quantification of exhausted T cells using specific markers such as PD-1 and Tim-3.
5. Adoptive Transfer and Bone Marrow Transplant Experiments:
  - Adoptive transfer of T cells from WT and Oma1KO mice into recipient mice to evaluate their effects on hepatic immunogenicity and tumor progression.
  - Bone marrow transplant experiments to assess the contribution of hematopoietic cells to hepatic immunosurveillance in the absence of parenchymal OMA1.
6. Hepatocyte-Specific Deletion of Oma1:
  - Generation of hepatocyte-specific Oma1 knockout mice using Cre-loxP system.
  - Evaluation of NRF2 signaling activation, hepatocyte death, and hepatic immunosurveillance in these mice compared to controls.

### Mouse handling

All animal procedures conformed to the EU Directive 86/609/EEC and Recommendation 2007/526/EC regarding the protection of animals used for experimental and other scientific purposes, enforced by Spanish law under Real Decreto 1201/2005. Approval of the different experimental protocols required the estimation of the adequate sample size as well as the definition of the randomization and blinding criteria. Mice under endpoint criteria were excluded from analysis in long-term experiments. Mice were fed a standard chow diet. Initially, chow diet was Rod18 (LASQCdiet^®^), but from December 2021 onwards it was progressively changed to D184 (SAFE^®^).

Mice were predominantly housed at the specific pathogen-free (SPF) area, and, more rarely, at the conventional area (e.g., animals submitted to bone marrow transplantation or studied through imaging techniques like MRI). The temperature at the animal facility was kept at 22 °C ± 2. Relative humidity oscillated between 45 and 65%, though values closer to 45% were the most common. An artificial 12-h light cycle was maintained: lights were on from 06:00 A.M. to 06:00 P.M. during wintertime, while during summertime, the lights-on period went from 7:00 A.M. to 7:00 P.M. This enables mice to keep their light cycles unaltered.

### Mouse breeding

Constitutive *Oma1*^KO^ mice in a mixed C57BL/6/Sv129 background were originally generated by Quiros et al, as described in their methods (Quiros et al, 2012). These *Oma1*^KO^ (C57BL/6/Sv129) mice were further backcrossed more than 20 generations to inbred C57BL/6JOlaHsd mice, obtaining *Oma1*^KO^ mice in a pure C57BL/6JOlaHsd background. *Oma1*^WT^ C57BL/6JOlaHsd mice were regularly backcrossed to Harland’s C57BL/6JOlaHsd mice. Regular backcrosses of *Oma1*^KO^ mice to *Oma1*^WT^ C57BL/6JOlaHsd mice were performed to prevent genetic drift. Sample size and age of experimental animals are stipulated for every experiment.

Hepatocyte-specific *Oma1*-deleted mice (*Oma1*^H-KO^) were obtained by mating the Albumin-Cre mice line with the *Oma1*^fl/fl^ mice line. Albumin-Cre mice were originally generated by Postic et al, 1999 (Postic et al, 1999) and made commercially available by Jackson Laboratory. *Oma1*^fl/fl^ mice were originated by Prof. Dr. Thomas Langer (Wai et al, 2015), who generously sent them to us. Female C57BL/6JOlaHsd mice were artificially inseminated with epididymal sperm from *Oma1*^fl/fl^ mice, whose genetic background was C57BL/6N. Obtained heterozygous mice (in a mixed OlaHsd/N background) were crossed among them to generate homozygous *Oma1*^fl/fl^ animals. Female *Oma1*^fl/fl^ mice were further crossed with male Albumin-Cre mice, whose genetic background was C57BL/6J, obtaining heterozygous *Oma1*^wt/fl^ Albumin-Cre positive mice in a mixed OlaHsd/N/J background. Heterozygous *Oma1*^wt/fl^ Albumin-Cre positive mice were mated to generate littermate *Oma1*^wt/wt^ and *Oma1*^fl/fl^ Albumin-Cre positive mice: *Oma1*^H-WT^ and *Oma1*^H-KO^ animals. Sample size and age of experimental animals are stipulated for every experiment.

### Survival experiments

Survival was monitored in 10–24 animals per experimental group. Mouse age at natural death was considered the end-survival. When an animal’s condition was regarded as incompatible with continued survival by an independent veterinarian, animals were euthanized and represented as deaths in the curve. Dead animals were macroscopically analyzed at necropsy to determine the cause of death and, when possible, their tissues were further collected for histopathological analysis. Animals belonging to survival experiments were additionally monitored for body weight evolution. Mice in these two experiments were not used for functional or blood tests to avoid any potential confounding interventional factor. For statistical analysis, Log-rank (Mantel–Cox) tests were performed.

To analyze survival in male constitutive *Oma1*^WT^ (*n* = 37) and *Oma1*^KO^ (*n* = 25) mice, two independent experiments were carried out. For the second experiment, *Oma1*^KO^ mice were generated after an additional backcross to *Oma1*^WT^ C57BL6/JOlaHsd mice, to discard potential genetic drift effects in the observed phenotype. Median and maximum survival in male *Oma1*^KO^ mice were identical in both experiments. To study survival in female constitutive *Oma1*^WT^ (*n* = 15) and *Oma1*^KO^ (*n* = 15) mice, one survival experiment is being carried out. To study survival in male *Oma1*^WT^ *Cox7a2l*^113^ (*n* = 10) and *Oma1*^KO^ *Cox7a2l*^113^ (*n* = 19) mice, one survival experiment is being carried out.

### Histopathology analysis

Tissue samples were fixed in 4% paraformaldehyde solution in PBS 1×, processed, and embedded in paraffin. Sections (6–12 μm) were prepared and mounted on coverslips for staining with Hematoxylin and Eosin (H&E) or Picrosirius Red (PSR), or to perform immunohistochemistry and visualize Ki67, F4/80, and Caspase-3 (Casp3). H&E was used for the staining of nuclei and cytoplasmic inclusions in liver sections. Ki67 IHC was employed to mark proliferating cells. F4/80 was used to visualize macrophages in liver sections. Caspase-3 IHC was aimed at marking apoptotic cells. PSR was used for collagen deposition staining in liver sections.

Images were captured using a NanoZoomer 2.0RS digital slide scanner (Hamamatsu) and were digitized using NDP view2 Software. H&E staining was analyzed qualitatively by an independent histopathologist. For quantitative analysis of Ki67, F4/80, Casp3, and PSR staining, several macros were developed to quantify the percentage of areas of interest. An initial macro was obtained to extract single TIFF files from the *.czi* scans. Sequentially, a second macro calculating the stained areas vs. the total analyzed tissue was run on the generated TIFF files. Extensive liver areas (~ 90 mm^2^) were analyzed for each mouse, providing an unbiased, broad result from our measurements of interest. Microgranuloma areas were manually delimited using NDP view2 Software tools. Qualitative identification criterium for microgranuloma areas was F4/80-positive areas containing macrophage clusters. Microgranuloma areas (μm^2^) were normalized to the total area of analyzed liver tissue.

### Liver RNA extraction

RNA was extracted from mouse tissues using TRI-reagent (Sigma) or the RNeasy® Mini Kit (Qiagen), depending on the latter use of the extracted RNA. Briefly, tissues were collected at necropsy and immediately frozen in 2-ml Eppendorf tubes by including them in liquid nitrogen. When RNA was extracted to perform qRT-PCR, TRI-reagent was added to the samples at 4 °C, and homogenization was performed using a TissueRuptor II (Qiagen) homogenizer. RNA was extracted from homogenates by phenol-chloroform-based phase separation and isopropanol precipitation. RNA from four wild-type and three OMA1^KO^ was extracted for transcriptomics, the RNeasy® Mini Kit was followed, controlling for RNA integrity at its obtention. Final RNA pellets were diluted in milliQ water and quantified with the NanoDrop (Thermo Scientific™).

### Bulk liver RNAseq

For transcriptomics analysis, the reads from fastq files from all samples were pre-processed to remove adapters using Trimgalore v-0.6.6 (https://www.bioinformatics.babraham.ac.uk/projects/trim_galore/). Resulting trimmed reads were mapped on the mouse transcriptome (Ensembl gene-build GRCm38.v91) and genome, using STAR v2.5.1 and RSEM v1.3.1. Genes with less than 10 counts per million in each sample were filtered out. Then, expected counts were TMM-normalized using edgeR v-3.36.0. Then, differentially expressed genes between wild-type and OMA1^KO^ were identified using the limma v-3.50.3 bioconductor package, previous transformation and calculation of variance weights with the voom function.

### Bulk liver proteomics

Liver biopsies protein extracts were obtained from liver samples of 4 control and 4 Oma1^KO^ by tissue homogenization with ceramic beads (MagNa Lyser Green Beads, Roche, Germany) in CS buffer (Pipes pH 6.8, MgCl_2_, NaCl, EDTA, sucrose, SDS, sodium orthovanadate; Biochain Institute, Inc. #K3013010-5), freshly supplemented with protease and phosphatase inhibitors. Extracted proteins (around 200 µg) were subjected to in-filter reduction and alkylation using iodoacetamide, followed by trypsin digestion (Nanosep Centrifugal Devices with Omega Membrane-10K, PALL), and the resulting peptides were TMT-labeled following the manufacturer’s instructions. Labeled peptides were subjected to LC-MS analysis using a Proxeon Easy nano-flow HPLC system (Thermo Fisher Scientific, Bremen, Germany) coupled via a nanoelectrospray ion source (Thermo Fisher Scientific) to an Orbitrap Fusion mass spectrometer (Thermo Fisher using with a 2-cm trap column and a 50-cm analytical column (75 µm I.D, 2 µm particle size, Acclaim PepMap RSLC, 100 C18; Thermo Fisher Scientific) in a continuous acetonitrile gradient consisting of 0–30% A for 60 min, 50–90% B for 3 min (A = 0.1% formic acid; B = 100% acetonitrile, 0.1% formic acid) at a flow rate of 200 nL/min. Mass spectra were acquired in a data-dependent manner, with an automatic switch between MS and MS/MS using a top-speed method and dynamic exclusion. MS spectra were collected in the Orbitrap analyzer using a mass range of 400–1500 *m/z* at 60,000 resolution. HCD fragmentation was performed at 33 eV of normalized collision energy, and MS/MS spectra were analyzed at 30,000 resolution in the Orbitrap.

Protein identification was performed using the SEQUEST HT algorithm integrated in Proteome Discoverer 2.5 (Thermo Scientific). MS/MS scans were searched against a mouse reference proteome database (mouse_202306_pro-sw.target-decoy.fasta), (296316 sequences in total). For database searching, parameters were selected as follows: trypsin digestion with 2 maximum missed cleavage sites, precursor mass tolerance of 2 Da, and fragment mass tolerance of 0.03 Da. Methionine oxidation ( + 15.994915 Da) and asparagine and glutamine deamidation ( + 0.984016 Da) were set as variable modifications, while cysteine carbamidomethylation ( + 57.021464 Da) and TMT labeling ( + 229.162932 Da) at the peptide N-terminal end and Lys were considered as fixed modifications. False discovery rates (FDR) of peptide identifications were calculated using the refined method (Navarro and Vázquez, 2009) with an additional filter for precursor mass tolerance of 10 ppm (Bonzon-Kulichenko et al, 2015). 1% FDR was used as a criterion for peptide identification.

Quantitative information from TMT reporter intensities was integrated from the spectrum level to the peptide level, and then to the protein level, based on the WSPP model (Prieto and Vázquez, 2020; Trevisan-Herraz et al, 2019) using the GIA integration algorithm (García-Marqués et al, 2016). Relative protein abundances were expressed as standardized log2-ratios (Zq).

For differential proteomic analyses, proteins represented by more than one different peptide were selected for differential expression analysis. Subsequently, peptides differentially expressed between *Oma1*^WT^ and *Oma1*^KO^ were discovered using limma v-3.50.3 from the Zq values.

### IPA

Functional analysis was carried out by using IPA (Ingenuity Knowledge Database, http://www.ingenuity.com), applying a threshold of Benjamini–Hochberg method, adjusted *P* value < 0.05, in both RNAseq and proteomic. Then, resulting pathways and predicted upstream regulators were selected for a minimum size effect of z-score ≥ 2 or z-score ≤ −2 and Benjamini–Hochberg adjusted *P* values ≤ 0.05.

### Frailty assessment (Valencia Score)

The “Valencia Score” was performed to evaluate frailty in the old animals (Gomez-Cabrera et al, 2017). The score includes the measurement of the following 5 components: unintentional weight loss (change in body weight), low activity level (motor coordination), weakness (grip strength), poor endurance (total running time in the incremental treadmill test), and slowness (maximal running speed in the incremental treadmill test). The frailty score for each age group of animals was calculated as follows: total number of tests failed by the animals at each age group, divided by the total number of tests performed by these animals, expressed in percentage of frail mice. For more details, see (Gomez-Cabrera et al, 2017).

#### Grip strength

A modified protocol from Gómez-Cabrera et al (Gomez-Cabrera et al, 2017) was used. Briefly, mice were acclimated to the Grip Strength Meter (Panlab, Harvard Apparatus) for 2 days, performing three trials for every mouse with 5 min resting time between attempts. On the day of the experiment, the procedure was repeated, and maximal strength was registered for their three attempts. Since mice’s body weight influences their strength, relative grip strength was determined by dividing grip strength by the animal’s weight. For the analysis, maximal values achieved by every mouse were considered (including those values from acclimatation days). Diverse groups of male mice aged 10, 50, and 80 weeks old in the context of frailty evaluation were used for these experiments.

#### Hanging endurance

To evaluate muscle strength and resistance, we performed a hanging endurance test with a square grid (1 cm^2^ grid), where animals grabbed using their four extremities against gravity. When an animal was placed on the grid, it was immediately turned upside-down (180°), and the time the mouse endured in a hanging position with its four limbs was registered. This trial was assessed three times for every mouse, allowing mice to rest at least 5 min between attempts, and the longest recorded latency time to fall was considered for the statistical analysis.

#### Rotarod

To evaluate motor coordination, we used the rotarod (Panlab, Harvard Apparatus). Mice were acclimated to the rotarod for 2 days: mice were placed on the rotarod at 4 rpm—until they gained stability for 60 s—to later increase velocity (from 4 to 40 rpm in 5 min). Three attempts were carried out for every mouse. On the day of the experiment, mice were placed on the rotarod, and velocity was increased from 4 to 40 rpm in 5 min. When mice fell from the rotarod, their time registry was annotated. Three attempts were carried out for every mouse with 5 min of rest between each attempt. For the analysis, maximal values achieved by every mouse were considered (including those values from acclimatation days). Diverse groups of male mice aged 10, 50, and 80 weeks old in the context of frailty evaluation were used for these experiments.

#### Treadmill

A modified protocol from Gómez-Cabrera et al (Gomez-Cabrera et al, 2017) was used. Briefly, diverse groups of male mice aged 10, 50, and 80 weeks old were used for frailty evaluation. Mice were acclimated to running on a motorized treadmill (Panlab, Harvard Apparatus) for 2 days. The first day, mice ran for 6 min at 10 cm/s and 4 min at 14 cm/s. The second day, mice ran for 6 min at 10 cm/s, 4 min at 14 cm/s and 4 min at 16 cm/s. On the day of the experiment, mice ran at 10 cm/s for 4 min and then velocity was raised 4 cm/s every 2 min. When mice spent 2 s at the electric shock pad at the back of the treadmill, they were removed from the experiment, annotating the maximal velocity and the total running time each mouse had achieved.

### Allograft tumor model

*Oma1*^WT^ E0771 and *Oma1*^KO^ E0771 cell lines were generated using CRISPR/Cas9-mediated gene editing technology, as described previously (Ran et al, 2013). On the allograft tumor injection day, E0771 cells must be growing at an exponential rate. Cells were detached from tissue culture plastic with Trypsin-EDTA (Gibco™) and resuspended in PBS 1× and Matrigel®. In total, 1 × 10^6^ E0771 cells, in a volume of 32.5 μl PBS 1× and 17.5 μl Matrigel®, were injected in the flank of nude mice. Tumor volume (V = [length·(width)^2^]/2) was monitored using calipers. Twenty-three days later, when allografts reached a mean estimated volume of 0.6 cm^3^, mice were sacrificed, and their tumors analyzed.

### Chemically induced tumor model

Chemically induced tumors were induced in 21-week-old male C57BL/6JOlaHsd mice by a single intramuscular injection of 1 mg 3-methylcholanthrene (3MC) (Sigma-Aldrich) into their left rear leg (*n* = 10 *Oma1*^WT^ and *n* = 9 *Oma1*^KO^). 3MC was dissolved in sesame oil (Sigma) at 25 mg/ml. Body weight and tumor growth were monitored regularly. Differential leg volumes (as an estimate of fibrosarcoma growth) were analyzed through magnetic resonance imaging at three different timepoints: 30, 60, and 90 days after 3MC-injection. Mice were prematurely euthanized when: (i) tumor volume reached 1.5 cm (*n* = 1), (ii) tumors showed ulcerative processes (*n* = 2), and (iii) mice experienced >25% body weight loss (*n* = 1). The remaining mice were sacrificed 90 days after the injection.

In vivo MRI was performed at CNIC’s Advanced Imaging Unit with an Agilent/Varian scanner (Agilent, Santa Clara, USA) equipped with a DD2 console, an active-shielded 115-60 gradient insert coil, and a two-channel volume coil (NeosBiotec, Spain). Mice were anesthetized with 2% isoflurane (Abbott) and oxygen and positioned on a thermoregulated (37 °C) mouse bed with continuous monitoring of the respiratory cycle. Ophthalmic gel was placed in the eyes to prevent retinal drying. Consecutive axial 1 mm thick slices were acquired to image the tumor. Images were acquired in free-breathing animals, using a gradient-echo sequence with 4 ms/150 ms echo/repetition times, a 20° flip angle, 5 averages, 100 kHz bandwidth, and a 3 cm × 3 cm field of view with a matrix of 128 × 128 pixels.

### qRT-PCR

Reverse transcriptase (RT) reaction was performed using: 2 μg of the extracted RNA, a reverse transcriptase enzyme (MultiScribeTM), random primers, dNTPs, milliQ water, and the RT buffer (all belonging to Invitrogen). Obtained 20 μl cDNA solutions were diluted in milliQ water to a final volume of 100 μl and stored at −20 °C. Real-time cDNA amplification was performed using Applied Biosystems™ Power SYBR™ Green PCR Master Mix.

The efficiency and dissociation curves from every pair of primers used were tested before the amplification experiments. Pairs of primers presenting various peaks in their dissociation curves were discarded. For those presenting normal dissociation curves morphology, efficiency was calculated using a standard cDNA curve. A regression curve for every pair of primers was obtained by correlating log[cDNA] and the Ct for every given cDNA concentration, calculating its slope. Efficiency for those primers was 10^(-1/slope)^, and only primers presenting >1.8 and <2 efficiency were selected. The list of tested and used primers is included in the Supplementary Reagents and Tools Table.

qRT-PCR results were analyzed using the SDS 2.4 and Quant Studio v.1.4.3 (Applied Biosystems) software. Ct median values from every three technical replicates were used to calculate relative levels of a target RNA, using β-actin as the normalizing gene.

### Blood collection and plasma analysis

Blood was extracted by submandibular vein puncture on immobilized conscious animals using a 4–5 mm animal lancet (Goldenrod, MEDIpoin Inc). A maximal total volume of 500 μl was collected at every blood extraction procedure. When blood extraction was performed for posterior biochemistry analysis, a collecting Microvette® 200 or 500 EDTA was used. Anticoagulated samples were centrifuged for 12 min at 10,000 × *g* at 4 °C. Supernatant plasma was collected and transferred to 1.5 mL sterile Eppendorf tubes. Varying volumes of plasma were assessed with the Dimension RxL Max analyzer.

Max analyzer obtained data on blood concentration of: alanine transaminase (ALT-GPT), aspartate transaminase (AST-GOT), albumin, direct bilirubin, total bilirubin, blood urea nitrogen (BUN), low-density lipoproteins (LDL), high-density lipoproteins (HDL), total cholesterol, triglycerides, non-esterified fatty acids (NEFA), and lipase, among others.

### Subcellular fractionation

Mice were euthanized by cervical dislocation. Tissues were collected at necropsy and immediately frozen in 2-ml Eppendorf tubes by including them in liquid nitrogen. Tissues were defrosted briefly at 4 °C and homogenized right away in medium A (320 mM sucrose, 10 mM Tris-HCl, 1 mM EDTA, pH 7.4) supplemented with 0.1% fatty acid-free BSA with a glass tissue grinder using a motor-driven Teflon pestle (Heidolph RZR 2041) with 9–15 strokes at 600 rpm. Homogenates were centrifuged for 10 min at 4 °C at 1000×*g*. Resulting pellets were kept as “nuclear fraction”, and supernatants were isolated as “cytosolic fractions”. Protein concentration was quantified using the Bradford method (Gomez-Cabrera et al, 2017) (BioRad).

### Mitochondria extraction

Mitochondria were extracted from mice and cells as described previously (Gomez-Cabrera et al, 2017). Mice were euthanized by cervical dislocation. The liver was removed and immediately cooled down to 4 °C in PBS 1×. Tissue was cut and homogenized in medium A (320 mM sucrose, 10 mM Tris-HCl, 1 mM EDTA, pH 7.4) supplemented with 0.1% fatty acid-free BSA with a glass tissue grinder using a motor-driven Teflon pestle (Heidolph RZR 2041) with 9–15 strokes at 600 rpm. Homogenates were centrifuged for 10 min at 4 °C at 1000 × *g*. Resulting supernatants were collected to fresh new 2 ml Eppendorf tubes and centrifuged for 10 min at 4 °C at 10,000 × *g*. Supernatants were discarded, and mitochondria-enriched pellets were washed with medium A.

### ATP synthesis assay

ATP production was measured in freshly extracted liver mitochondria by a luminescence assay, where light production indicated increasing ATP concentrations. In total, 10–40 μg of liver mitochondria were resuspended in buffer A (KCl 150 mM, Tris-HCl 25 mM, EDTA 2 mM, 0.1% BSA fatty acid free, KPO4 10 mM, MgCl_2_ 0.1 mM, pH 7.4) at RT and dispensed into the wells (96-well luminescence reading plate, Costar).

Substrate cocktail and buffer B (Tris-acetate 0.5 M, pH 7.75, luciferine 0.4 mM, luciferase 10 μg/ml) were added, and luminescence was measured over 2 min in a plate luminometer (Orion Microplate Luminometer, Simplicity 4.2 software). Substrate cocktails contained diadenosin pentaphosphate 6 mM and ADP 6 mM supplemented with glutamate/malate (5 and 2 mM, respectively) for determination of CI activity-coupled ATP production or octanoyl-carnitine/malate (200 μM and 100 μM, respectively) for determination of β-oxidation-coupled ATP production.

A standard ATP curve was performed as a calibration method (from 0 to 10 mM final concentration of ATP). A regression curve was obtained by correlation of ATP concentration and luminescence. ATP production rate is expressed as a percentage of “nmol of ATP/min/mg of protein vs. control”. In the final plots, each dot represents mitochondria from an individual mouse; each sample is analyzed using technical triplicates.

### Seahorse experiment

Oxygen consumption in liver mitochondria (2 μg /well) was measured using the XF96 Analyzer (Seahorse Bioscience), as described before (Acin-Perez et al, 2020). Calibration was performed overnight at 37 °C in an atmosphere without CO_2_. Liver mitochondria respiration was stimulated using NADH or succinate ( + rotenone) (1st injection) to analyze complex I or complex II-dependent oxygen consumption rate (OCR), following injection of antimycin A + rotenone (2nd injection) to inhibit RCs-dependent respiration. Subsequent TMPD + ascorbate (3rd injection) was added to obtain complex IV-dependent respiration, and azide (4th injection) was used to inhibit complex IV-dependent respiration. Final drug concentrations were 1 μM. Oxygen consumption rates were normalized to protein concentration using the Bradford method (Gomez-Cabrera et al, 2017) (BioRad).

### Confocal microscopy

#### Lipid droplets visualization

Small liver samples were placed in 4% paraformaldehyde solution in PBS 1× during necropsy and left at RT overnight. Samples were washed in PBS 1× and incubated with staining mix (Bodipy-493/503 1 μg/ml, DAPI (1/1000), 1% Triton X100, 2% BSA, 0.1% NaAz in PBS 1×) overnight at 4 °C in a spinning wheel. Samples were washed and left in glycerol 70% overnight at 4 °C in a spinning wheel. Liver samples were mounted and visualized under a Leica SP5 confocal microscope. Quantification of the relative Bodipy to DAPI signal was obtained using Fiji.

#### Lymphocytes visualization

Livers were dissected and fixed in 4% PFA overnight. Organs were dehydrated in 70% EtOH and embedded in paraffin blocks. Sections of livers (5μm) were cut with a microtome (Leica). Sections were deparaffined and rehydrated. Antigen retrieval was performed by boiling the samples in sodium citrate 10 mM using a microwave. After cooling, the samples were washed in distilled water. Samples were blocked with 10% Donkey Serum (Millipore) and 1% Triton and further incubated with primary antibodies overnight. After washing, livers were incubated with conjugated secondary antibodies (1:200, Jackson Laboratories) and DAPI at room temperature. Samples were mounted with Fluoromount-G (SouthernBiotech).

The following primary antibodies were used: Purified Rat Anti-Mouse CD31 (1:400, 553370 Pharmingen); rabbit anti-ERG- Alexa647 (1:200, Abcam, Ab110639); Monoclonal rat anti-Endomucin (1:400, sc-53941 Santa Cruz Biotechnology); Purified Rat Anti-Mouse CD45 (1:200, BD Biosciences, Clone 30-F11 (RUO)). Liver sections were imaged at high resolution with a Leica SP8 confocal microscope ×20 objectives were used for confocal scanning. Individual fields or tiles of large areas were acquired. Fiji/ImageJ codes were used to automatically threshold, select and quantify object areas in confocal micrographs: Autofluorescence signal and DAPI were used to determine the total area of liver tissue, DAPI selection was used to determine the total number of cells, CD45 selection was used to determine the number and area of immune cells. CD45 cluster area and frequency were determined too. These areas were used to calculate the following properties: (1) CD45% = 100*CD45 counts/DAPI counts and (2) CD45 area = 100* CD45 area/Liver area.

### Denaturing gel electrophoresis

Sodium dodecyl sulfate polyacrylamide gel electrophoresis (SDS-PAGE) was performed for protein separation in denaturing conditions. Loading buffer (50 mM Tris-HCl pH 6.8, 2% SDS, 10% glycerol, 1% 2-mercaptoethanol, 0.02% bromophenol blue) was added to protein samples and incubated for 5 min at 95 °C. Samples were loaded into a polyacrylamide gel. Polyacrylamide gel concentration was 12.5%. Electrophoresis was performed in a Mini-PROTEAN Tetra cell (Biorad) using Tris-glycine buffer (Tris 25 mM, glycine 20 mM, SDS 0.5%), at 60 V during the stacking of the proteins and at 100–120 V once the samples reached the separating gel.

### Western blot

Separated proteins in a gel were electroblotted onto PVDF transfer membranes (Immobilon-FL, 0.45 μm, Merck Millipore, IPFL00010) for 1 h at 100 V in 4 °C transfer buffer (48 mM Tris, 39 mM glycine, 20% methanol). A Mini Trans-Blot Cell system (BioRad) was used. Electroblotted membranes were dried and washed with methanol. PBS 1× was used to wash methanol away before the blocking step. 5% BSA in PBS 0.1% Tween-20 buffer was used as a blocking solution for 1 h at room temperature (RT). Blocked membranes were incubated with primary antibodies. Primary antibodies used include: NRF2 (Santa Cruz), GAPDH (Abcam), H3 (Abcam), ATF4 (Cell Signaling), P-eIF2a (Cell Signaling), ß-Actin (Sigma). Immunoblotted membranes were incubated with fluorescent secondary antibodies for 45 min at RT. Membranes were washed with PBS 0.1% Tween-20, and fluorescent detection was performed with the Odyssey® scanner (LI-COR Biosciences). Quantification of relative protein content was performed using Fiji.

### Lymphoid cell isolation from blood

In total, 200 µl of blood was collected as previously stated in cold 2 mM EDTA PBS. Erythrocytes were removed using twice 5 ml of red blood cell lysis buffer (0.15 M ammonium chloride, 0.01 M sodium bicarbonate, 0.1 mM EDTA) for 5 min at 4 °C. After washing, the cells were ready to stain.

### Lymphoid cell isolation from the liver

Mice were euthanized using CO_2_ narcosis followed by perfusion with cold PBS. Liver was harvested and minced in pre-warmed 25 mM HEPES 10% FBS RPMI supplemented with 0.4 mg/ml collagenase type VIII (Sigma, C2139) under shaking at 180 rpm for 45 min at 37 °C. Digested tissue was then filtered through a 70-μm cell strainer and centrifuged at 350 × *g* for 5 min at 4 °C. Erythrocytes were removed using 5 ml of red blood cell lysis buffer for 5 min at 4 °C. After washing, supernatants were centrifuged in a 40%/70% Percoll gradient (Sigma, GE17-0891-01) at 1250 × *g* for 30 min at RT with acceleration on 6 and without brake to further enrich in leucocytes. Isolated cells were washed with PBS and resuspended in 1 ml 2% FBS RPMI for counting.

### Flow cytometry

#### Surface staining

Total blood cells, or 5 × 10^6^ cells from the liver or the spleen, were incubated with anti-CD16/32 (1:200, BD Pharmingen, 553142) and Ghost Dye Violet 540 (1:1000, Tonbo Bioscience, 13-0879-T500) for 15 min at 4 °C. After washing, surface staining was performed using fluorochrome-conjugated antibodies diluted in Brilliant Stain Buffer (BD Bioscience, 566349) for 30 min at 4 °C.

#### Intracellular staining

For transcription factor staining, cells were fixed in Foxp3/Transcription Factor Staining Buffer (eBioscience, 00-5523-00) for 30 min at RT. After washing with its Permeabilization Buffer, cells were incubated with the fluorochrome-conjugated antibodies diluted in Permeabilization Buffer overnight at 4 °C.

Samples were acquired using a 4-Laser (V/B/YG/R) Aurora flow cytometer and the Spectroflo software (Cytek Biosciences), with compensation beads as controls. Data were analyzed using the FlowJo software (BD Biosciences).

### High-dimensional flow cytometry analysis

Multiparametric analysis of spectral flow cytometry data was performed utilizing the OMIQ platform (www.omiq.ai) after preliminary cleaning of debris, dead cells, and aggregates. First, the flowCut algorithm was run to remove anomaly-acquired events in all files analyzed. Then, CD45^+^ cells were selected and subsampled 10,000–30,000 events, followed by dimension reduction and clustering using unsupervised uniform manifold approximation and projection (UMAP. Neighbors = 15, Minimum Distance = 0.4, Components = 2, Learning Rate = 1, Epochs = 200) and ClusterX (Alpha = 0.001) algorithms, respectively, to visualize the different CD45^+^ subsets in groups. Finally, results were plotted and identified depending on their mean fluorescence marker expression.

### Plasma cytokines measurement

Serum cytokines were quantified using multiplexed magnetic bead technology (Invitrogen, ProcartaPlex Mouse Th1/Th2 Cytokine Panel).

### Adoptive transfer assay

Spleen and lymph nodes were harvested from 10-week-old Ly5.1 (CD45.1) mice, mashed through a 70-μm cell strainer, and resuspended in 5 mL of Red Blood Cell Lysis Buffer (ammonium chloride 0.15 M, sodium bicarbonate 0.01 M, EDTA 0.0001 M, pH 7.4) for 5 min at 4 °C. Cells were resuspended in 4 mL of MojoSort^TM^ Buffer and filtered again through a 70-μm cell strainer. Total CD8^+^ T cells were isolated using MojoSort^TM^ Mouse CD8^+^ T Cell Isolation Kit (BioLegend) following the manufacturer’s instructions. Isolated cells were counted, checked for purity, and resuspended in sterile saline solution (0.9% NaCl). Finally, 10 × 10^6^ CD8^+^ T cells were intravenously injected to recipient mice.

### Bone marrow transplant assay

Recipient *Oma1*^WT^ and *Oma1*^KO^ male mice were lethally irradiated using two 5.5 Gy split doses separated by 3 h, and subsequently received bone marrow donor cells from female *Oma1*^WT^ and *Oma1*^KO^ mice via retro-orbital injection. Donor bone marrow cells were harvested by flushing both femora into Red Blood Cell Lysis Buffer, centrifuged, and resuspended in PBS 1× for their subsequent injection into irradiated mice.

### Primary hepatocytes isolation

Mice were anesthetized with isoflurane to ensure deep, sustained anesthesia during the procedure. Mice were placed on their back on a warm, absorbent pad, and their limbs were secured. 70% ethanol was used to sterilize their abdomen. Once the liver was exposed, the inferior vena cava was canulated for perfusion, opening the portal vein to allow for perfusion outflow. Pre-warmed buffers used for perfusion were first a calcium-free solution containing chelating agents (0.3 mM EGTA, 22 mM D-Glucose, 123 mM NaCl, 5 mM KCl, 25 mM NaHCO_3_, 1 mM KH_2_PO_4_, 1 mM MgSO_4_ pH 7.4), second an EGTA-free calcium-free washing solution, and third an enzymatic digestion buffer containing collagenase I (Worthington, Ref LS004196) and CaCl_2_. After digestion, the liver was excised and placed in culture buffer (MEM, FBS 10%, glutamine, P/S, where it was further mechanically teased apart. The final hepatocyte cell suspension was filtered through a sterile gauze, submitted to three washing steps, and seeded into 48-well plates for downstream experiments. Primary hepatocytes were cultured at 37 °C in a 5% CO_2_ incubator for 6 h before exchanging their culture buffer with one containing 5% FBS (MEM, 5% FBS, glutamine, P/S) until the day of the experiment.

### Co-culture experiments

Primary hepatocytes coming from *Oma1*^WT^ and *Oma1*^KO^ mice were seeded in 48-well plates, at a confluency of 2 × 10^4^ hepatocytes per well, and incubated with or without 1 mM N-acetylcysteine (NAC). In parallel, *Oma1*^WT^ CD8^+^ T cells were isolated as described above (see “Adoptive transfer assay” in “Methods”). After counting and assessing purity, CD8⁺ T cells were resuspended in complete RPMI medium (supplemented with 10% FBS, 2 mM glutamine, 50 nM β-mercaptoethanol, and 25 mM HEPES) and seeded at a density of 1.5 × 10⁶ cells/mL in 6-well culture plates. Cells were activated with αCD3/CD28 Dynabeads (20 µL per 1 × 10⁶ CD8⁺ T cells; Thermo Fisher, 11452D) and 20 ng/mL IL-2 (PeproTech, 212-12) for 48 h at 37 °C and 5% CO₂. After activation, αCD3/CD28 Dynabeads were removed from CD8⁺ T cells using a cell culture magnet. Cells were resuspended in complete RPMI medium supplemented with 20 ng/mL IL-2, and 2 × 10⁵ CD8⁺ T cells were added to primary hepatocytes with or without 1 mM NAC. After 5 days of co-culture, CD8⁺ T cells were harvested and processed for flow cytometry analysis as previously described (see “Flow cytometry” in “Methods”).

### Human data at GepLiver

GepLiver is a longitudinal and multidimensional liver expression atlas integrating transcriptomic profiles of human tissues, mouse models, and single cells to depict stepwise liver transitions across a full range of developmental stages and liver disease phases. Human transcriptomics data comparing *Oma1*, *Opa1*, and *Dele1* expression to liver disease-associated markers, including liver inflammation and liver tumor stage, were obtained using this database.

### Statistical analysis

Sample size for every measurement was based on previously reported results and varied depending on the type of data and/or study. The experiments were not randomized. The investigators were blinded to the animal experimental group in the in vivo imaging analysis.

Data are represented as mean values ± standard deviation (SD) in dot plots. Where applicable, normality was estimated using D’Agostino and Pearson or Shapiro–Wilk normality test. For comparison between two groups, paired or unpaired *t* test was used. When data presented a normal distribution, a *t* test was used, and the Mann–Withney test otherwise. For more than two groups, data were evaluated by one-way analysis of variance (ANOVA) with Dunnett’s multiple comparison (when data presented normal distribution) or Kruskal–Wallis with Dunn ´s multiple comparison (when data did not follow a normal distribution). When data on a quantitative dependent variable was collected for two categorical independent variables (say time and genotype, or genotype and treatment), we applied a two-way ANOVA or a Mixed-effects model (with Geisser-Greenhouse correction). A mixed-effects model was used when a group of individuals was tested several times (and in fact “time” was one of the independent variables), whereas a two-way ANOVA was applied when all samples belonged to different individuals.

Statistically significant outliers were identified using Grubb’s test (ESD method). All statistical analyses were performed using Prism v6 (GraphPad Software, California, USA) if not specified. A *P* value below 0.05 was considered statistically significant.

## Supplementary information

Peer Review File

Source data Fig. 1

Source data Fig. 2

Source data Fig. 3

Source data Fig. 4

Source data Fig. 5

Source data Fig. 6

Source data Fig. 7

Expanded View Figures

## Data Availability

Cell lines and mouse strains will be available under a materials transfer agreement (MTAs). All data are available in the main text or the supplementary materials. Proteomic data are available at: PRIDE: PXD076790. Transcriptomic data are available at GEO: GSE283516. The source data of this paper are collected in the following database record: biostudies:S-SCDT-10_1038-S44318-026-00839-4.
