# Supplementary material for: OMA1 protects from liver injury and tumorigenesis during aging by controlling hepatic immunogenicity
Source: EMBO J. 2026 Jun 23;45(15):5292–329. doi: 10.1038/s44318-026-00839-4 (PMC13434532; doi:10.1038/s44318-026-00839-4)
Supplement: Supplementary file 1 [file 44318_2026_839_MOESM1_ESM.pdf]

OMA1 protects from liver injury and tumorigenesis during aging by controlling hepatic immunogenicity

Yolanda Martí-Mateos, María del Mar Muñoz-Hernández, Manuel M. Gómez de las Heras, José Ignacio Escrig-Larena, José Luis Cabrera-Alarcón, Rebeca Acín-Pérez, Enrique Calvo, Sara Natalia Jaroszewicz, Lucía de Prado-Rivas, Eva Raquel Martínez-Jiménez, Macarena de Andrés Laguillo, Esther García-Domínguez, Mari Carmen Gómez-Cabrera, José Viña, Rui Benedito, Alejo Efeyan, Jesús Vázquez, María Mittelbrunn, María Concepción Jiménez-Gómez and José Antonio Enríquez

Corresponding author: Jose Antonio Enriquez (jaenriquez@cnic.es)

Review Timeline:

Submission Date:	30th Sep 24
Editorial Decision:	4th Oct 24
Appeal:	16th Oct 24
Editorial Decision:	14th Jan 25
Revision Received:	6th Apr 26
Editorial Decision:	21st May 26
Revision Received:	27th May 26
Accepted:	1st Jun 26

Editors: Cornelius Schneider / Daniel Klimmeck

Transaction Report:

(Note: With the exception of the correction of typographical or spelling errors that could be a source of ambiguity, letters and reports are not edited. Depending on transfer agreements, referee reports obtained elsewhere may or may not be included in this compilation. Referee reports are anonymous unless the Referee chooses to sign their reports.)

1st Editorial Decision 4th Oct 2024

2nd Oct 2024

Re: EMBOJ-2024-119185
OMA1 protects from liver injury and tumorigenesis during aging by controlling hepatic immunogenicity

Dear Prof. Enriquez,

Thank you for submitting your manuscript to The EMBO Journal. We have now considered it within our editorial team, and unfortunately concluded that we cannot offer its publication. Please note that since we publish only a small percentage of the many manuscripts that we receive at The EMBO Journal, we can subject only those submissions to in-depth review that would appear as sufficiently compelling candidates for an EMBOJ article already during our initial editorial assessment.

I am sorry that we cannot be more positive for The EMBO Journal on this occasion, but very much hope that you will find this transfer option worthwhile.

Yours sincerely,

Cornelius Schneider
on behalf of the EMBOJ editorial team

Cornelius Schneider, PhD
Editor
The EMBO Journal
c.schneider@embojournal.org

Dear Editor

I would like to have the opportunity to discuss your decision. Since I may have fail to transmit why we are convince that this work merits to be reviewed and eventually published in the interest of the EMBO J readers.
I will be happy to discuss it by phone or y video meeting.

Best

JAE

Dear Dr Enriquez,

Thank you again for the submission of your manuscript (EMBOJ-2024-119185R-Q) to The EMBO Journal, and providing us with a preliminary point-by-point response to the concerns raised by the referees. As mentioned, your study was assessed by three reviewers with expertise in liver biology, inflammation signaling and ageing, whose comments are enclosed below.

As you will see from their comments, the referees acknowledge the analysis and potential interest and value of your findings. However, they also express major concerns i.p. regarding the degree of mechanistic insights into the connection between OMA1 and Nrf2 oxidative stress response signalling (ref#1, pt.7; ref#2, pt.4). Reviewer #2 also states the need to complement with various rescue experiments to adequately consolidate the claims made (ref#2, pts3,5). Referee #3 is concerned about the relevance of the liver-specific depletion model (ref#3, pt.9). Further, there are requests of various additional analyses required on the biochemical and genetics side.

Given the overall interest stated and broader angle of your results, we are able to invite you to revise your manuscript experimentally to address the referees' comments, along the lines sketched in your outline. I need to stress though that we do require strong support from the referees on a revised version of the study in order to move on to publication of the work.

Please feel free to contact me if you have any questions or need further input on the referee comments.

We generally allow three months as standard revision time. As a matter of policy, competing manuscripts published during this period will not negatively impact on our assessment of the conceptual advance presented by your study. However, we request that you contact the editor as soon as possible upon publication of any related work, to discuss how to proceed. Should you foresee a problem in meeting this three-month deadline, please let us know in advance and we may be able to grant an extension.

When submitting your revised manuscript, please carefully review the instructions below.

Please feel free to approach me any time should you have additional questions related to this.

Thank you for the opportunity to consider your work for publication.

I look forward to your revision.

Best regards,

Daniel Klimmeck

Daniel Klimmeck, PhD
Senior Editor
The EMBO Journal

Instruction for the preparation of your revised manuscript:

1) a .docx formatted version of the manuscript text (including legends for main figures, EV figures and tables). Please make sure that the changes are highlighted to be clearly visible.

2) individual production quality figure files as .eps, .tif, .jpg (one file per figure).

3) a .docx formatted letter INCLUDING the reviewers' reports and your detailed point-by-point response to their comments. As part of the EMBO Press transparent editorial process, the point-by-point response is part of the Review Process File (RPF), which will be published alongside your paper.

4) a complete author checklist, which you can download from our author guidelines (https://wol-prod-cdn.literatumonline.com/pb-assets/embo-site/Author Checklist%20-%20EMBO%20J-1561436015657.xlsx). Please insert information in the checklist that is also reflected in the manuscript. The completed author checklist will also be part of the RPF.

5) Please note that all corresponding authors are required to supply an ORCID ID for their name upon submission of a revised manuscript.

6) It is mandatory to include a 'Data Availability' section after the Materials and Methods. Before submitting your revision, primary datasets produced in this study need to be deposited in an appropriate public database, and the accession numbers and database listed under 'Data Availability'. Please remember to provide a reviewer password if the datasets are not yet public (see https://www.embopress.org/page/journal/14602075/authorguide#datadeposition).
In case you have no data that requires deposition in a public database, please state so in this section. Note that the Data Availability Section is restricted to new primary data that are part of this study.
*** Note - All links should resolve to a page where the data can be accessed. ***

7) Our journal encourages inclusion of *data citations in the reference list* to directly cite datasets that were re-used and obtained from public databases. Data citations in the article text are distinct from normal bibliographical citations and should directly link to the database records from which the data can be accessed. In the main text, data citations are formatted as follows: "Data ref: Smith et al, 2001" or "Data ref: NCBI Sequence Read Archive PRJNA342805, 2017". In the Reference list, data citations must be labeled with "[DATASET]". A data reference must provide the database name, accession number/identifiers and a resolvable link to the landing page from which the data can be accessed at the end of the reference. Further instructions are available at <https://www.embopress.org/page/journal/14602075/authorguide#referencesformat >.

8) At EMBO Press we ask authors to provide source data for the main and EV figures. Our source data coordinator will contact you to discuss which figure panels we would need source data for and will also provide you with helpful tips on how to upload and organize the files.

Numerical data can be provided as individual .xls or .csv files (including a tab describing the data). For 'blots' or microscopy, uncropped images should be submitted (using a zip archive or a single pdf per main figure if multiple images need to be supplied for one panel). Additional information on source data and instruction on how to label the files are available at <https://www.embopress.org/page/journal/14602075/authorguide#sourcedata >.

9) We replaced Supplementary Information with Expanded View (EV) Figures and Tables that are collapsible/expandable online (see examples in https://www.embopress.org/doi/10.15252/embj.201695874). A maximum of 5 EV Figures can be typeset. EV Figures should be cited as 'Figure EV1, Figure EV2" etc. in the text and their respective legends should be included in the main text after the legends of regular figures.

- For the figures that you do NOT wish to display as Expanded View figures, they should be bundled together with their legends in a single PDF file called *Appendix*, which should start with a short Table of Content. Appendix figures should be referred to in the main text as: "Appendix Figure S1, Appendix Figure S2" etc. See detailed instructions regarding expanded view here: <https://www.embopress.org/page/journal/14602075/authorguide#expandedview >.

- Additional Tables/Datasets should be labelled and referred to as Table EV1, Dataset EV1, etc. Legends have to be provided in a separate tab in case of .xls files. Alternatively, the legend can be supplied as a separate text file (README) and zipped together with the Table/Dataset file.

10) When assembling figures, please refer to our figure preparation guideline in order to ensure proper formatting and readability in print as well as on screen:
http://bit.ly/EMBOPressFigurePreparationGuideline

Please remember: Digital image enhancement is acceptable practice, as long as it accurately represents the original data and conforms to community standards. If a figure has been subjected to significant electronic manipulation, this must be noted in the figure legend or in the 'Materials and Methods' section. The editors reserve the right to request original versions of figures and the original images that were used to assemble the figure.

11) For data quantification: please specify the name of the statistical test used to generate error bars and P values, the number (n) of independent experiments (specify technical or biological replicates) underlying each data point and the test used to calculate p-values in each figure legend. The figure legends should contain a basic description of n, P and the test applied. Graphs must include a description of the bars and the error bars (s.d., s.e.m.).

Further information is available in our Guide For Authors: https://www.embopress.org/page/journal/14602075/authorguide

The revision must be submitted online within 90 days; please click on the link below to submit the revision online before 14th Apr 2025.

Referee #1:

The study by Martí-Mateos and colleagues investigates the role of the mitochondrial stress integrator OMA1 in hepatic inflammation, chronic liver disease (CLD) progression, and tumorigenesis. The authors demonstrate that OMA1 deletion in mice leads to increased oxidative stress, chronic liver inflammation, T cell exhaustion, and a significant rise in hepatic tumor incidence during aging. By integrating proteomics, transcriptomics, and genetic models, the study links mitochondrial dysfunction to immune dysregulation in the liver, notably T cell exhaustion. The findings emphasize the significance of OMA1 in liver immunosurveillance and provide novel insights (previously overlooked) into the complex regulation of tissue health by OMA1.

The work offers a compelling mechanistic framework connecting mitochondrial stress, oxidative signaling, and immune exhaustion with hepatic tumorigenesis, and it has implications for both basic science and clinical strategies targeting OMA1.The claims are novel and supported by rigorous experimental evidence

The manuscript is a tour de force that makes a number of important discoveries that are relevant and, at times unexpected, both for liver physiology and mitochondrial biology. It is experimentally thorough and sound and the impressive use of mouse models (beyond the simple KO) and the use of adoptive transfers and genetics enables the study to delineate effects with cell/tissue autonomy, which adds an important layer of mechanistic specificity. Importantly, the conclusions are not overblown, and the interpretations of the data are sound. I found the discussion to be well-balanced and thoughtful. The following are some general comments.

The manuscript provides strong evidence that OMA1 deletion exacerbates liver dysfunction, immune exhaustion, and tumorigenesis through NRF2-mediated oxidative stress and altered immunogenicity. This is based on the NRF2 relocalization to the nucleus and downstream transcriptional remodeling. The data convincingly support the conclusion that hepatocyte-specific OMA1 expression is critical for maintaining liver function and immunosurveillance, filling a key knowledge gap in mitochondrial biology and liver disease. The manuscript succinctly discusses its findings in the context of previous literature, particularly highlighting the complexities of interpreting OMA1's roles in mice with a mixed genetic background. The authors force us to reconsider the interpretation of this literature.

Certain sections, such as those detailing histological findings were difficult to follow. I do not have the expertise to determine whether the histological findings fully support the claims of various tumor types and/or whether the molecular analyses are in line with these findings. The authors describe a very significant and subtle phenotype of liver dysfunction in young OMA1 KO mice, which they argue sets the stage for age-associated liver dysfunction later. This sounds reasonable, yet it would be helpful to know whether this is in fact true. While causality is challenging to establish, I wondered: has this been observed elsewhere?

The manuscript occasionally overstates the therapeutic relevance of OMA1-targeted therapies, particularly considering the

observed adverse effects of OMA1 deletion in this and other cited studies. While the manuscript includes a thorough review of prior studies, it could expand on the clinical contexts where NRF2 activation has been shown to be protective in liver disease, which would lend additional credence to their study.

The methodology for proteomics and transcriptomics analyses is sound but could include more details on data normalization and statistical thresholds for differential expression. In addition, the study would benefit from supplementary data providing more detailed raw data for histological analyses. I was unable to evaluate the raw datasets for RNAseq and therefore cannot comment on their worth. It would be helpful to have extended histological images, to aid reproducibility and clarity.

In the end, I am still left with the outstanding question: through which substrate(s) does the metalloprotease OMA1 orchestrate the phenotypes documented in this manuscript, if not for DELE1 (ISR) or OPA1 (mitochondrial structure)?

Referee #2:

In the present manuscript, the authors report a novel physiological role for the mitochondrial stress-sensitive protease OMA1 in hepatic immunosurveillance during the liver aging. They identify OMA1 as a crucial factor, the loss of which leads to a sustained activation of the KEAP1-NRF2 oxidative stress signaling pathway, which in turn promotes liver injury and chronic hepatic inflammation and progressive immune exhaustion. These events are further associated with an increased incidence of liver tumors and impaired survival in mice. The experiments described in this study are generally of high technical quality and clearly establish the role of OMA1 in immunosurveillance and prevention of chronic hepatic inflammation. The physiological roles of OMA1 remain incompletely understood and the protease is intensely investigated due to its role as a prospective target in several pathologies including cancers, so the reported advances are significant and will have high visibility. However, the manuscript has several shortcomings summarized below.

Major comments:
"OPA1 revealed a positive correlation with tumor severity (Fig 1B), in agreement
with the fact that OPA1 is a proteolytic target of OMA1." OMA1 mediates processing of OPA1 isoforms in a stress-dependent manner, but overall does not affect the GTPase's steady-state levels. Therefore, it is not entirely clear what this statement means as the figure refers to OPA1 expression.

The results demonstrating elevated NRF2 levels in OMA1 deleted cells and tissues are very interesting. However, the molecular weight of the band reported as NRF2 appears to be significantly distinct from what is typically reported in the literature (70-100 kDa). Also, the quality of western blots shown in Fig 4D is subpar. The nuclear fractions contain high levels of GAPDH (in some cases apparently as high as 30%), which further suggests suboptimal quality of their subcellular fractionations. Finally, Fig 7G would benefit from a representative Western blot demonstrating the quality of subcellular fractionation.

Would reducing NRF2 levels (e.g. by using available NRF2-targeting PROTACs) ameliorate the immune exhaustion effect seen in OMA1 KO livers? That could be a direct way to probe the authors' model.

Although clearly interesting and exciting, the present manuscript misses the mark in establishing a clear mechanistic link between OMA1 deletion and KEAP1-NRF2 associated effects. The proposed OMA1-mediated iron homeostasis dysregulation is an interesting hypothesis but there is virtually no evidence provided to back it up.

Do OMA1 KO livers or hepatocytes show altered GSH/GSSG ratios? Related to that, could administering mitochondria-targeted antioxidants such as MitoQ alleviate the negative effect of OMA1 deletion?

The authors should consider evaluating liver tolerance to mechanical and chemical challenges (e.g. hydrodynamic tail vein injection or acetaminophen challenge) to better support their conclusions regarding liver injury in aged OMA1 KO animals. Related to that, have they assessed levels of liver-produced GDF15 and FGF21 in the OMA1 KO mice?

Several independent reports established that loss of OMA1 leads to an apoptotic resistance. How do these findings fit with the enhanced hepatocyte death observed by the authors?

The part that addresses genetic background-specific effects is very interesting. Do previously reported cold sensitivity phenotypes in OMA1 KO animals relate to liver effects reported in the present study? Or were those effects specific to a certain genetic background too?

Minor comments:
The statement that OMA1 is protective in neurodegeneration is not entirely correct. While the Korwitz et al. study indeed had

shown that the loss of OMA1 was beneficial in their experimental model, other studies (e.g. Sprenger et al. 2019 EMBO Mol. Med.) reported a rather opposite effect of OMA1 deletion in a different cellular model of neurodegeneration. Therefore, the effects of OMA1 deletion in the context of neurodegeneration are more nuanced and need to be acknowledged that way.

Fig 3C. Direct bilirubin is down, whereas liver damage marker enzymes are up. These results are somewhat counterintuitive and need to be explained better.

The Fig 5 legend formatting is off.

Referee #3:

Marti-Mateos and collaborators analyses the role of the mitochondrial stress integrator OMA1 in hepatic inflammation and cancer progression. It highlights that deletion of OMA1 leads to increased primary liver tumor incidence and reduced survival in mice. This is associated with persistent activation of the KEAP1-Nrf2 oxidative stress pathway, early liver injury, chronic inflammation, and fibrosis. The absence of OMA1 also results in the accumulation of exhausted T cells, impairing immune surveillance. Further experiments suggest that hepatocyte-specific OMA1 deletion is sufficient to trigger NRF2 signaling, hepatocyte death, and increased liver immunogenicity. These findings emphasize the importance of OMA1 in maintaining hepatic health and immune surveillance. The analysis on the role of OMA1 on liver injury and tumorigenesis is novel. Nevertheless, the manuscript contains several significant issues, and the conclusions drawn are not adequately supported by the provided data.

Major Concerns

1- The provided patient data is insufficiently detailed to draw definitive conclusions. Key information such as the number of patients, tumor types, histological and biological parameters, and etiologies are missing. To establish a correlation between OMA1 expression and CLD development, a more comprehensive analysis is necessary, including comparisons with normal liver and preneoplastic tissues as cirrhotic tissue.
2- Figure EV1: The authors cannot definitively conclude that OmaKo animals exhibit a higher incidence of liver fibrosis based solely on a single histological image without quantification of F4/80 and PSR staining.
3- Similarly, the percentage of adenoma and HCC quantified in Oma1 ko tumoral livers lacks supporting histological analyses (Figure 2). The data are weak with only a single image of hepatoadenoma.
4- The transcriptomics and proteomics analyses (Figure 2D-E) lack clarity regarding the number of animals included. Additionally, it is unclear which specific samples (adenoma or HCC) were used for the tumoral analyses. To further substantiate the conclusions drawn that absence of OMA1 accelerates tumorigenesis by enhancing tumor aggressiveness, histological analyses of epithelial-to-mesenchymal transition, HNF4 alpha expression on liver tumoral tissue would be valuable. We imagine that theses specific features are related to Oma1 ko HCC tissue and not adenoma??
5- Why the validation of the implication of OMA1 on tumor aggressiveness is next perform on mouse breast cancer cells??? Could the authors also perform the same type of experiments with liver cancer cells?
6- Figure 4: in order to demonstrate that oxidative stress is elevated in young Oma1ko livers, it will be important to not only analyzed NRF2 expression. Others markers on redox control systems could be analyzed, DHE staining on tissue liver section can be an option.
7- Figure 5: At 50 weeks of age, Oma1ko livers exhibited characteristics of cholangiohepatitis. How does this finding relate to the observed progression of adenoma and hepatocellular carcinoma (HCC) in aging mice? Do they observed at this age an increased of oxidative stress (NRF2 signaling)?
8- It will be also important to show that exhausted CD8+ and CD4+ T cells gradually accumulate in Oma1KO livers in ageing mice when they developed tumors.
9- It will be also important to show that exhausted CD8+ and CD4+ T cells gradually accumulate in Oma1KO livers It will be also important to show that exhausted CD8+ and CD4+ T cells gradually accumulate in Oma1KO livers in ageing mice when they developed tumors.
10- Data on Albumin-Cre, Oma1fl/fl animals are really weak. We do not understand whether deleting Oma within hepatocytes has specific impact on liver pathologies as presented for the total KO. Do these mice present develop adenoma, HCC or cholangiohepatitis?
11- Data on Albumin-Cre, Oma1fl/fl animals are really weak. We do not understand whether deleting Oma within hepatocytes has specific impact on liver pathologies as presented for the total KO. Do these mice present develop adenoma, HCC or cholangiohepatitis?

Referee #1, additional cross-comment:

I have read the comments from Referee 2 and Referee 3 and fully agree with their points. The comment "Although clearly interesting and exciting, the present manuscript misses the mark in establishing a clear mechanistic link between OMA1 deletion

and KEAP1-NRF2 associated effects. The proposed OMA1-mediated iron homeostasis dysregulation is an interesting hypothesis but there is virtually no evidence provided to back it up." is spot on. In essence, it is unclear how the loss of the OMA1 metalloprotease triggers the phenotypes described from a molecular perspective. At the cellular level, it appears from Referee 3, that the claims regarding tumorigenesis are far from solid. I am not an expert in tumor biology, but it appears as though the data do not fully support the claims of the paper and this is concern. I had concerns regarding the early phenotypes the mice present and the very late-stage manifestation of liver dysfunction and it seems the proposed experiments and questions by Referee 3 underscore the same concerns about the underlying cellular mechanisms, kinetics and causality. The most novel discovery that is worth reporting is the immune remodelling/Tcell exhaustion and liver dysfunction that arises in vivo and it is important that these claims be solid. Whether the substrates of OMA1 (if it is in fact the catalytic activity of the protease that is requires) that mediate the signalling of NRF2-KEAP are formally identified is less of a concern for me as long as the cellular mechanism(s) are clear. Ideally the manuscript would be able to clarify or at least provide meaningful insights to both molecular and cellular mechanisms.

That being said, if OMA1-dependent NRF2-KEAP signalling is truly hepatocyte-specific, it should be possible to explore this process either in primary hepatocytes from these mice or perhaps in hepatocyte-like cell lines (AML12, Hepa1.6, HEPG2, Huh7) in which OMA1 activity is silenced. It is possible that the mitotically active cell-lines do not faithfully recapitulate what is seen in non-dividing, binucleate primary hepatocytes - this has been seen by several groups. It is also possible that the primary hepatocytes require a far longer time to generate oxidative damage, death, and immunogenicity than their standard culture time (e.g. 48 hours) allows, although nuclear translocation should be visible. If the NRF2-KEAP axis is a conserved pathway regulated by OMA1, it should be possible to explore this in OMA1 KO MEFs that the lab surely has/has access to. Alternatively, the proposed NRF2-KEAP axis activation is simply a consequence of progressive tissue dysfunction brought on by the loss of OMA1.

REF: EMBOJ: EMBOJ-2024-119185R-Q (OMA1 protects from liver injury and tumorigenesis durin...)

<u>Editor Remarks</u>:

The referees acknowledge the analysis and potential interest and value of your findings. However, they also express major concerns i.p. regarding:

1. The degree of mechanistic insights into the connection between OMA1 and Nrf2 oxidative stress response signaling (ref#1, pt.7; ref#2, pt.4).
   We have repeated and extended NRF2 WBs, demonstrating that NRF2 signaling is still active in *Oma1*^{KO} livers during aging. Furthermore, we have found a NRF2-dependent FGF21 repression in *Oma1*^{KO} livers across ages, further supporting this signaling.
2. Reviewer #2 also states the need to complement with various rescue experiments to adequately consolidate the claims made (ref#2, pts3,5).
   This point has been hard to address due to technical constrains. The ideal model system for these rescue experiments requires performing aging experiments with newly generated genetic mouse models, or with chemical interventions. In either case these experiments would require 2-3 years to be performed. Trying to find a trade-off between suitability and feasibility, we have successfully implemented an experimental set up that allow us to evaluate our main claims. It consists in exposing wild type in vitro CD8$^+$ T cells to isolated primary hepatocytes (wild type or *Oma1*^{KO}) and assess if, as we propose, CD8$^+$ T cells are differentially impacted by the co-culture with hepatocytes. This set up allow us to clearly demonstrate after 5 days of co-culture that of *Oma1*^{KO} hepatocytes induce CD8$^+$T cell exhaustion. In addition, we could also demonstrate that this is prevented by the preincubation of the *Oma1*^{KO} hepatocytes with the antioxidant NAC what should prevent the induction of NRF2-dependent mechanism. This in vitro rescue experiment confirm our claim that hepatocyte ROS mediated persistent induction of NRF2 signaling in *Oma1*^{KO} causes increasing immunogenicity in hepatocytes and, subsequently, promote immune exhaustion.

3. Referee #3 is concerned about the relevance of the liver-specific depletion model (ref#3, pt.9).
   We have extended our analysis of the liver-specific depletion model confirming that hepatocyte-specific *Oma1*-deletion leads to immune exhaustion *in vivo*, demonstrating that hepatocyte-specific *Oma1* expression modulates liver immunogenicity and immune exhaustion. Orthogonally, as mentioned in the previous point, we have shown that *Oma1*^{KO} isolated hepatocytes are sufficient to trigger increased exhaustion in co-culture with CD8$^+$ T cells. These two fundamental pieces of data definitively demonstrate that specific deletion of *Oma1* in hepatocytes is what triggers increased immunogenicity, immune exhaustion and subsequent premature tumorigenesis, filling an important gap in knowledge with relevant clinical implications.
4. Further, there are requests of various additional analyses required on the biochemical and genetics side.
   We performed most of these requests.

   Additionally, we have finished the survival study for female *Oma1*^{KO} mice (in a pure C57BL/6JOlaHsd background) and for male *Cox7a2l*^{113} *Oma1*^{WT} and *Oma1*^{KO} mice (**Figure R1**). Interestingly, we found that median *Oma1*^{KO} survival in females is partially rescued, in line with epidemiological data for chronic liver disease in MASLD and HCC patients (PMID: 21623852 and 14623619), where males are especially vulnerable. Regarding male *Cox7a2l*^{113} mice, there was still a significant survival reduction *Oma1*^{KO} mice compared to their controls, demonstrating yet in a different genetic background that these findings hold true. It is interesting to note that *Cox7a2l*^{113} mice presented an augmented median survival overall, which is of high relevance given the extended use of the C57BL/6J mouse strain, that presents the inactive version of this protein (*Cox7a2l*^{111}).

Figure for reviewers removed.

Referee Remarks:

- **Reviewer access details for Proteomic data:**
  Log in to the PRIDE website using the following details:
  Project accession: PXD076790
  Token: 2Ad7cAFSRCbI
  Alternatively, reviewer can access the dataset by logging in to the PRIDE website using the following account details:
  Username: reviewer_pxd076790@ebi.ac.uk
  Password: 90ItKCA2TzWj

- **Reviewer access details for Transcriptomic data:**
  Log in to the GEO website using the following details
  Project accession: GSE283516
  https://www.ncbi.nlm.nih.gov/geo/query/acc.cgi?acc=GSE283516
  Password Token: ulutsgwsfhmnrcn

Referee #1:

The study by Martí-Mateos and colleagues investigates the role of the mitochondrial stress integrator OMA1 in hepatic inflammation, chronic liver disease (CLD) progression, and tumorigenesis. The authors demonstrate that OMA1 deletion in mice leads to increased oxidative stress, chronic liver inflammation, T cell exhaustion, and a significant rise in hepatic tumor incidence during aging. By integrating proteomics, transcriptomics, and genetic models, the study links mitochondrial dysfunction to immune dysregulation in the liver, notably T cell exhaustion. The findings emphasize the significance of OMA1 in liver immunosurveillance and provide novel insights (previously overlooked) into the complex regulation of tissue health by OMA1.

The work offers a compelling mechanistic framework connecting mitochondrial stress, oxidative signaling, and immune exhaustion with hepatic tumorigenesis, and it has implications for both basic science and clinical strategies targeting OMA1.The claims are novel and supported by rigorous experimental evidence.

We thank the reviewer for the positive comments.

The manuscript is a tour de force that makes a number of important discoveries that are relevant and, at times unexpected, both for liver physiology and mitochondrial biology. It is experimentally thorough and sound and the impressive use of mouse models (beyond the simple KO) and the use of adoptive transfers and genetics enables the study to delineate effects with cell/tissue autonomy, which adds an important layer of mechanistic specificity. Importantly, the conclusions are not overblown, and the interpretations of the data are sound. I found the discussion to be well-balanced and thoughtful.

We are sincerely grateful to the reviewer for this encouraging evaluation. We are particularly pleased that the reviewer recognized the 'experimentally thorough and sound' nature of the work, and the value of our diverse mouse models in defining effects of cell/tissue autonomy.

The following are some general comments. The manuscript provides strong evidence that OMA1 deletion exacerbates liver dysfunction, immune exhaustion, and tumorigenesis through NRF2-mediated oxidative stress and altered immunogenicity. This is based on the NRF2 relocalization to the nucleus and downstream transcriptional remodeling. The data convincingly support the conclusion that hepatocyte-specific OMA1 expression is critical for maintaining liver function and immunosurveillance, filling a key knowledge gap in mitochondrial biology and liver disease. The manuscript succinctly discusses its findings in the context of previous literature, particularly highlighting the complexities of interpreting OMA1's roles in mice with a mixed genetic background. The authors force us to reconsider the interpretation of this literature.

Certain sections, such as those detailing histological findings were difficult to follow. I do not have the expertise to determine whether the histological findings fully support the claims of various tumor types and/or whether the molecular analyses are in line with these findings. The authors describe a very significant and subtle phenotype of liver dysfunction in young OMA1 KO mice, which they argue sets the stage for age-associated liver dysfunction later. This sounds reasonable, yet it would be helpful to know whether this is in fact true. While causality is challenging to establish, I wondered: has this been observed elsewhere?

We thank the reviewer for their thoughtful observation. C57BL/6 mice are known to naturally develop hepatic tumors during aging (PMID: 1324182). We do not propose that OMA1 depletion cause the generation of any particular type of tumor, instead what we show is that the same panoply of different hepatic tumors naturally observed in this mouse strain have a more premature onset when OMA1 is ablated. No causal relation of OMA1 depletion and any particular type of tumor is suggested, but rather an acceleration of a known age-related phenotype. We have succinctly clarified this point in our revised text.

However, the early liver microgranuloma phenotype young *Oma1*^{KO} mice present has previously been described in models for liver injury (PMID: 8287121) or liver infection (PMID: 15617528). Therefore, the increased presence of these histological findings in these young mice indicate premature liver damage. These references have been included to the text for clarity.

1. The manuscript occasionally overstates the therapeutic relevance of OMA1-targeted therapies, particularly considering the observed adverse effects of OMA1 deletion in this and other cited studies. While the manuscript includes a thorough review of prior studies, it could expand on the clinical contexts where NRF2 activation has been shown to be protective in liver disease, which would lend additional credence to their study.

Thank you for the suggestion. Given the complexities of NRF2 signaling and its implication in liver disease we agree it is valuable to discuss it further in our manuscript. While published literature found NRF2 signaling to be protective upon acute liver disease, it has been shown that upon chronicity this signaling can become insufficient or even negative (PMID: 25294219, 27211490 and 39286246). We have made sure to clarify this in the text, to provide an enriched background for our findings.

2. The methodology for proteomics and transcriptomics analyses is sound but could include more details on data normalization and statistical thresholds for differential expression. In addition, the study would benefit from supplementary data providing more detailed raw data for histological analyses. I was unable to evaluate the raw datasets for RNAseq and therefore cannot comment on their worth. It would be helpful to have extended histological images, to aid reproducibility and clarity.

We apologize for the insufficient information provided regarding the omics data. We have explained the normalization and statistical procedures in the new version of material and methods. We provided access through the editor to all proteomic and transcriptomic raw data, and as far as we know that there were no issues in accessing them. Regarding the histological data, we will provide the extended raw histology images with the updated revised manuscript.

3. In the end, I am still left with the outstanding question: through which substrate(s) does the metalloprotease OMA1 orchestrate the phenotypes documented in this manuscript, if not for DELE1 (ISR) or OPA1 (mitochondrial structure)?

We do believe that it is mainly by OPA1, key regulator of mitochondrial structure. Mild overexpression of OPA1 showed increased tumorigenesis susceptibility in humans, which goes in line with a decreased activity of OMA1 (its inactivating protease). Additionally, the fact that mitochondria fragmentation participates in beta-oxidation regulation (PMID: 36917141) –which is compromised in *Oma1*^{KO} hepatocytes– suggests that OMA1's structural role through OPA1 proteolysis is what causes the hepatocyte metabolic pathology. In fact, lipid degradation is known to be compromised in *Oma1*^{KO}

animals (PMID: 22433842). Thus, OPA1-dependent mitochondrial morphology is the most likely driver behind this fascinating and complex hepatic phenotype. We have included a concise comment on this speculative mechanism in the revised manuscript.

Referee #2:

In the present manuscript, the authors report a novel physiological role for the mitochondrial stress-sensitive protease OMA1 in hepatic immunosurveillance during the liver aging. They identify OMA1 as a crucial factor, the loss of which leads to a sustained activation of the KEAP1-NRF2 oxidative stress signaling pathway, which in turn promotes liver injury and chronic hepatic inflammation and progressive immune exhaustion. These events are further associated with an increased incidence of liver tumors and impaired survival in mice. The experiments described in this study are generally of high technical quality and clearly establish the role of OMA1 in immunosurveillance and prevention of chronic hepatic inflammation. The physiological roles of OMA1 remain incompletely understood and the protease is intensely investigated due to its role as a prospective target in several pathologies including cancers, so the reported advances are significant and will have high visibility.

We thank the reviewer for the positive comments.

However, the manuscript has several shortcomings summarized below.

Major comments:

1. "OPA1 revealed a positive correlation with tumor severity (Fig 1B), in agreement with the fact that OPA1 is a proteolytic target of OMA1." OMA1 mediates processing of OPA1 isoforms in a stress-dependent manner, but overall does not affect the GTPase's steady-state levels. Therefore, it is not entirely clear what this statement means as the figure refers to OPA1 expression.

Thank you for bringing up this point, as we see how this statement could be misleading. While it is true that OMA1 mediates the processing of OPA1 isoforms and does not affect the GTPase's steady-state levels, stress-dependent OPA1 proteolysis by OMA1 is mostly inactivating in nature. We are aware of the proposed functions of short OPA1 forms (PMID: 28298442), but it is thought that only long OPA1 forms are responsible for mitochondrial fusion (PMID: 24616225 and 28298442). Therefore, total absence of OPA1 expression phenocopies mitochondrial fragmentation induced by OMA1 overactivation, while overexpression of OPA1 tends to phenocopy the hyperfused mitochondria network typical of OMA1 deletion (PMID: 17003040, 24055366 and 24550258). In fact, it has been shown that mild OPA1 overexpression induces tumorigenesis in mice (PMID: 32315597). We have clarified this point in the text to limit their cited relation to mitochondrial fusion activity.

2. The results demonstrating elevated NRF2 levels in OMA1 deleted cells and tissues are very interesting. However, the molecular weight of the band reported as NRF2 appears to be significantly distinct from what is typically reported in the literature (70-100 kDa). Also, the quality of western blots shown in Fig 4D is subpar. The nuclear fractions contain high levels of GAPDH (in some cases apparently as high as 30%), which further suggests suboptimal quality of their subcellular fractionations. Finally, Fig 7G would benefit from a representative Western blot demonstrating the quality of subcellular fractionation.

We agree with the reviewer that the manuscript would benefit from the improvements suggested. We obtained new nuclear and mitochondrial fractions and analyzed them by Western Blot. Our new results show that *Oma1*^{KO} mice present increased NRF2 nuclear localization, as previously shown in our manuscript (**Figure R2**). As fractionation quality has been enhanced in this new analysis, we have decided to replace our previous blot with these new images (**Figure 4** of Revised Manuscript).

Figure for reviewers removed.

3. Would reducing NRF2 levels (e.g. by using available NRF2-targeting PROTACs) ameliorate the immune exhaustion effect seen in OMA1 KO livers? That could be a direct way to probe the authors' model.

This is a great point. We acknowledge we did not include rescue experiments to support this part of our model, mainly due to the fact that such long *in vivo* experiments are in the scale of 2-3 years. However, there is extensive literature supporting that NRF2 activation happens as a protective response to hepatocyte stress acutely and that, upon chronicity, this signaling can become insufficient or even negative (PMID: 25294219, 27211490 and 39286246). Therefore, we would expect that reducing NRF2 levels might accelerate hepatocyte damage and exacerbate immune exhaustion.

4. Although clearly interesting and exciting, the present manuscript misses the mark in establishing a clear mechanistic link between OMA1 deletion and KEAP1-NRF2 associated effects. The proposed OMA1-mediated iron homeostasis dysregulation is an interesting hypothesis but there is virtually no evidence provided to back it up.

Our current interpretation is that NRF2 activation –which is known to be triggered by H_2O_2 production by mitochondria– relies in the inefficiency of β-oxidation in the absence of OMA1. To establish the role of NRF2 in the complex *Oma1*^{KO}-mediated effects, we established a primary hepatocytes co-culture model where we could assess if $CD8^+$ T cell exhaustion depends on the ROS-dependent NRF2 signaling (**Figure R3**). *Oma1*^{WT} and *Oma1*^{KO} primary hepatocytes in co-culture with $CD8^+$ T cells were treated with N-acetylcysteine (NAC), which blunts NRF2 signaling by scavenging reactive oxygen species (PMID: 34420083). NAC treatment abolished the *Oma1*^{KO}-dependent increase in exhaustion, while not affecting $CD8^+$ T cells co-cultured with *Oma1*^{WT} hepatocytes (**Figure R3** and **Figure EV7** of Revised Manuscript). This suggests that blunting NRF2 signaling rescues the *Oma1*^{KO}-mediated increase in immune exhaustion, mechanistically linking the observed phenotype in hepatocytes to the subsequent triggering of the immune axis.

Figure for reviewers removed.

Regarding iron homeostasis, we never claimed that our results support this hypothesis. Our mention through the discussion was purely speculative and we are open to remove it if the reviewers do not find it appropriate.

5. Do OMA1 KO livers or hepatocytes show altered GSH/GSSG ratios? Related to that, could administering mitochondria-targeted antioxidants such as MitoQ alleviate the negative effect of OMA1 deletion?

The study of *Oma1*^{KO} redox state would be of significant interest for the molecular definition of *Oma1* deletion in hepatocytes. However, we believe that the use of MitoQ would not have an easy interpretation. MitoQ, consist of redox-active quinones conjugated to lipophilic cations that accumulate in the mitochondrial matrix driven by the ΔΨmt. Once reduced to ubiquinol, MitoQ can limit lipid peroxidation and modulate mitochondrial redox status (PMID: 17014364). However, as a redox-cycling molecule, MitoQ may also influence ETC activity, thereby altering mitochondrial beyond simple scavenging (PMID: 15788391). Consequently, reductions in oxidative readouts following MitoQ treatment may reflect broader metabolic reprogramming rather than selective neutralization of mtROS. Other mitochondrial antioxidants like MitoTEMPO act a superoxide dismutase mimetic that removes superoxide and, in doing so, generates H_2O_2 (PMID: 17854275). Since H_2O_2 would then activate NRF2 (PMID: 36064071), it would increase NRF2 activation. Instead, we prefer to use N-acetylcysteine (NAC) to increases cysteine availability for glutathione synthesis boosting intracellular antioxidant capacity. Therefore, NAC treatment can provide a clearer interpretation, as it efficiently promotes the neutralization of H_2O_2, the direct activator of NRF2 signaling. More specifically, when NAC enters the cell, it is converted to L-cysteine, which is then used to build GSH, allowing glutathione peroxidase to neutralize H_2O_2. As NRF2 signaling stems from H_2O_2, this intervention allows to study the attenuation of NRF2 signaling in cells. Results for this intervention are included in the previous question and figure (**Figure R3** and **Figure EV7** of Revised Manuscript).

6. The authors should consider evaluating liver tolerance to mechanical and chemical challenges (e.g. hydrodynamic tail vein injection or acetaminophen challenge) to better support their conclusions regarding liver injury in aged OMA1 KO animals. Related to that, have they assessed levels of liver- produced GDF15 and FGF21 in the OMA1 KO mice?

Aged *Oma1*^{KO} livers show severe fibrosis and inflammation, therefore, they are likely less tolerant to any chemical or mechanical challenge. We do not see what inducing additional damage to the liver will add to our current study. On the other hand, we checked the plasma levels of liver-produced GDF15 and FGF21 in *Oma1*^{KO} vs. WT mice. Interestingly, plasma FGF-21 was significantly reduced in *Oma1*^{KO} livers (**Figure R4A**), while GDF-15 did not change across genotypes but only upon aging (**Figure R4B**), as previously reported (PMID: 35051643). These observations are consistent with our current model, as NRF2 is known to repress the synthesis of FGF-21 in a cell-autonomous way (PMID: 21852674 and **Figure R4C**). Thus, it would be expect that NRF2 upregulation in *Oma1*^{KO} hepatocytes would lead to a decrease in FGF-21 synthesis and secretion to the plasma, as shown here.

Figure for reviewers removed.

7.= Several independent reports established that loss of OMA1 leads to an apoptotic resistance.=How do these findings fit with the enhanced hepatocyte death observed by the authors?

The reviewer is correct, as OMA1 loss in most cell systems leads to apoptosis resistance, including previous experiments in our lab using mouse adult fibroblasts. In fact, this phenotype can explain the protective effect of *Oma1* deletion to ischemia/reperfusion injury in kidney, several insults causing heart failure, etc. All of them, to some extent, were assayed as acute stresses. What we see here is a condition that develops over months (years, if we translate it to the human equivalent) and whose expression is modulated by the genomic context. The trigger of apoptosis is an acute event but the exhaustion of the immune system derives from a chronic, sustained stress, derived from OMA1-dependent altered lipid metabolism in hepatocytes. We do not see any contradiction here.

8. The part that addresses genetic background-specific effects is very interesting. Do previously reported cold sensitivity phenotypes in OMA1 KO animals relate to liver effects reported in the present study? Or were those effects specific to a certain genetic background too?

The reviewer is again correct, cold sensitivity phenotypes in *Oma1*^{KO} animals are general to all the

genetic backgrounds that we have tested. Both in the mixed background and in the pure C57BL/6N genetic context (**Figure R5A-B**) *Oma1*^{KO} mice show impaired thermogenic ability compared to their control counterparts.

Figure for reviewers removed.

Minor comments:

1. The statement that OMA1 is protective in neurodegeneration is not entirely correct. While the Korwitz et al. study indeed had shown that the loss of OMA1 was beneficial in their experimental model, other studies (e.g. Sprenger et al. 2019 EMBO Mol. Med.) reported a rather opposite effect of OMA1 deletion in a different cellular model of neurodegeneration. Therefore, the effects of OMA1 deletion in the context of neurodegeneration are more nuanced and need to be acknowledged that way.

This is correct, however the nature of the mutation in the Sprenger paper is very special. As the reviewer probably knows, OPA1 is proteolytically processed by OMA1 but also by YME1L. The processing of each protease is specific, and they are triggered by different signals and pathways. This means that their function is not redundant. While in the study of Korwith et al. the lack of OMA1 is protective against the neurodegeneration caused by the loss of a prohibitin, in the study of Sprenger the lack of OMA1 exacerbates the neurodegeneration caused by the ablation of YME1L. Both are proteases that target OPA1. We have added a comment on this particular nuance to the manuscript.

2. Fig 3C. Direct bilirubin is down, whereas liver damage marker enzymes are up. These results are somewhat counterintuitive and need to be explained better.

One of the known functions of the liver is the conjugation of bilirubin which results in direct bilirubin. Therefore, liver dysfunction can compromise this conjugating function, while not affecting the levels of total bilirubin (also shown in our manuscript). We do not see a contradiction in this finding that needs further clarification.

3. The Fig 5 legend formatting is off.

Thank you for pointing this out. We have reviewed Fig 5 formatting and did not find errors, could you please clarify what the issue is?

Referee #3:

Marti-Mateos and collaborators analyses the role of the mitochondrial stress integrator OMA1 in hepatic inflammation and cancer progression. It highlights that deletion of OMA1 leads to increased primary liver tumor incidence and reduced survival in mice. This is associated with persistent activation of the KEAP1-Nrf2 oxidative stress pathway, early liver injury, chronic inflammation, and fibrosis. The absence of OMA1 also results in the accumulation of exhausted T cells, impairing immune surveillance. Further experiments suggest that hepatocyte-specific OMA1 deletion is sufficient to trigger NRF2 signaling, hepatocyte death, and increased liver immunogenicity. These findings emphasize the importance of OMA1 in maintaining hepatic health and immune surveillance. The analysis on the role of OMA1 on liver injury and tumorigenesis is novel. Nevertheless, the manuscript contains several significant issues, and the conclusions drawn are not adequately supported by the provided data.

Major Concerns

1. The provided patient data is insufficiently detailed to draw definitive conclusions. Key information such as the number of patients, tumor types, histological and biological parameters, and etiologies are missing. To establish a correlation between OMA1 expression and CLD development, a more comprehensive analysis is necessary, including comparisons with normal liver and preneoplastic tissues as cirrhotic tissue.

We believe this point needs clarification, as we have not provided any data from patients. We guess that the reviewer is referring to the information in Figure 1, that was obtained from GepLiver. As we explain in the manuscript GepLiver (http://www.gepliver.org/#/home)is a longitudinal and multidimensional liver expression atlas integrating transcriptomic profiles of human tissues, mouse models and single cells to depict stepwise liver transitions across a full range of developmental stages and liver disease phases. Human transcriptomics data comparing *Oma1*, *Opa1* and *Dele1* expression to liver disease associated markers, including liver inflammation and liver tumor stage was obtained using this database. During this year the group of Thomas Langer has identified two additional targets of *Oma1* (named *Dnajc15* and *Afm1*) in new version of the manuscript we show that the expression of none of the *Oma1* targets but *Opa1* correlate with tumor stage. We do not draw any conclusion here, we just illustrate how OMA1 expression level negatively impacts –while OPA1 expression level positively correlates– with liver tumor formation (while DELE1 does not show any correlation). We cannot provide more information than that available in GepLiver. We have not analyzed human material in our study.

2. Figure EV1: The authors cannot definitively conclude that OmaKo animals exhibit a higher incidence of liver fibrosis based solely on a single histological image without quantification of F4/80 and PSR staining.

We should clarify here that in Figure EV1 we just presented a representative histological image of the first identifications of liver pathology during autopsies in *Oma1*^{KO} mice. By no means we conclude anything based only in that finding. This figure serves as a mere hint towards the latter thorough analysis of liver pathology. Quantitative, longitudinal analyses on liver fibrosis and infiltrated macrophages (PSR and F4/80 stainings, respectively, as requested by the reviewer) in *Oma1*^{KO} livers throughout their life are already provided in Figure 5 of our manuscript (simplified here for reference, **Figure R6**).

Figure for reviewers removed.

3. Similarly, the percentage of adenoma and HCC quantified in Oma1 ko tumoral livers lacks supporting histological analyses (Figure 2). The data are weak with only a single image of hepatoadenoma.

We believe that we may have not explained properly the goal of this figure. Here, we just show different types of spontaneous tumor lesions observed in *Oma1*^{KO} livers at an age when wild type C57BL/6 mice do not present them yet. C57BL/6 mice are known to naturally develop hepatic tumors, whose characterization is not the focus of this manuscript. Moreover, we do not propose that OMA1 depletion causes the generation of any particular type of tumor, what we found is that the same panoply of hepatic tumors naturally observed in this mouse strain at 2 and a half years of age have their debut one year earlier when OMA1 is ablated. We do not propose causal relation of OMA1 depletion with any particular type of tumor. The only relevant point here is the early appearance of liver tumors.

4. The transcriptomics and proteomics analyses (Figure 2D-E) lack clarity regarding the number of animals included. Additionally, it is unclear which specific samples (adenoma or HCC) were used for the tumoral analyses. To further substantiate the conclusions drawn that absence of OMA1 accelerates tumorigenesis by enhancing tumor aggressiveness, histological analyses of epithelial-to-mesenchymal transition, HNF4 alpha expression on liver tumoral tissue would be valuable. We

imagine that theses specific features are related to Oma1 ko HCC tissue and not adenoma??

Thank you for noticing the need of clarification the number of animals in the -omic analysis (for transcriptomic 4 WT and 3 $Oma1^{KO}$; for proteomics: 4 WT and 4 $Oma1^{KO}$). In line with the previous answer, we never intended to study the tumors. All our analyses were focused in the liver tissue. Again, we should clarify that our conclusion is that OMA1 caused immune exhaustion and that this allows the early debut of naturally occurring tumors. What is very relevant is that immune exhaustion is locally occurring in the liver and caused by immune activation induced by hepatocyte metabolic alteration and not by cell-autonomous mechanisms. In fact, our analysis suggests the opposite: that tumor aggressiveness *per se* is not modified by the presence of OMA1 in tumor cells, as shown by our chemical-induced fibrosarcoma model in *Oma1*^{KO} and WT mice.

5. Why the validation of the implication of OMA1 on tumor aggressiveness is next perform on mouse breast cancer cells??? Could the authors also perform the same type of experiments with liver cancer cells?

Our intention with this experiment was to assess whether tumoral cells presented any advantage when growing in *Oma1*^{KO} vs. WT immunocompetent animals, in a cell-type agnostic way. Breast cancer E0771 cells were perfect for this purpose, as they were originally isolated from a naturally occurring tumor in a C57BL/6 mice, making them suitable for injection to immunocompetent mice. With this experiment, we demonstrated that the lack of OMA1 in the environment does not modify the intrinsic aggressiveness of WT tumoral cells.

6. Figure 4: in order to demonstrate that oxidative stress is elevated in young Oma1ko livers, it will be important to not only analyzed NRF2 expression. Others markers on redox control systems could be analyzed, DHE staining on tissue liver section can be an option.

We agree with the reviewer, as this would help us better define the stress that triggers NRF2 activation. Unfortunately, after using Amplex Red to measure H_2O_2 in isolated liver mitochondria, we found the measurements very noisy, so interpretation was very limited.

7. Figure 5: At 50 weeks of age, Oma1ko livers exhibited characteristics of cholangiohepatitis. How does this finding relate to the observed progression of adenoma and hepatocellular carcinoma (HCC) in aging mice? Do they observed at this age an increased of oxidative stress (NRF2 signaling)

At 50 weeks of age we find evidence of cholangiohepatitis in *Oma1*^{KO} livers, but they still need more than 40 weeks to progress to adenoma and HCC. Therefore, we do not expect these two phenomena to be linked in a simple and direct cause-effect way, but rather be part of the more complex non cell-autonomous mechanism we have thoroughly described.

On the other hand, to assess whether NRF2 signaling keeps being upregulated at more chronic timepoints, we analyzed liver samples of 70 weeks of age by WB. Interestingly, we showed that NRF2 signaling was still upregulated in aged *Oma1*^{KO} mice (**Figure R7**), demonstrating that this signaling never resolves and, in turn, it keeps activated chronically throughout their lives.

Figure for reviewers removed.

8. It will be also important to show that exhausted CD8+ and CD4+ T cells gradually accumulate in Oma1KO livers in ageing mice when they developed tumors.

Following the suggestion of the reviewer we have extended our previously observation showing that lymphocytes accumulate with aging in constitutive *Oma1*^{KO} mice. In the new version of the manuscript, through immunophenotyping, we have further broadened our observations to aged hepatocyte-specific *Oma1*^{KO} mice. With this analysis, we have demonstrated that specific *Oma1* deletion in hepatocytes is sufficient to trigger accumulation of exhausted CD8^{+} T cells (**Figure R8**). This is a major finding as it allows us to define the tissular pathway behind the OMA1- and age-dependent immune exhaustion and liver disease progression. We have included this data in the updated version of the manuscript (**Figure 7** of Revised Manuscript). This is in agreement with the co-culture experiments shown above.

Figure for reviewers removed.

9.= Data on Albumin-Cre, Oma1fl/fl animals are really weak. We do not understand whether deleting= Oma within hepatocytes has specific impact on liver pathologies as presented for the total KO. Do

these mice present develop adenoma, HCC or cholangiohepatitis?

As mentioned in the previous point, we have now analyzed in depth the phenotype of Albumin-Cre, Oma1fl/fl animals at 50 weeks of age. We found that just the ablation of OMA1 in hepatocytes reproduces the phenotype of immune exhaustion observed in the whole body *Oma1*^{KO} mice (**Figure R8** and **Figure 7** of Revised Manuscript). This confirms that the alteration of hepatocyte lipid metabolism due to OMA1 deletion is necessary and sufficient to induce local immune exhaustion in the liver, which then leads to early development of naturally occurring hepatic tumors.

Finally, to provide further evidence of this mechanism, we established an *ex vivo* co-culture assay with primary hepatocytes (*Oma1*^{KO} and WT) and CD8^{+} T cells (WT). With this orthogonal technique, we showed that *Oma1*^{KO} hepatocytes are uniquely efficient inducing exhaustion in WT CD8+ T cells (**Figure R9**), definitively demonstrating that it is the absence of OMA1 in hepatocytes what triggers a differential CD8^{+} T cell response, characterized by enhanced exhaustion. The results of this experiment have been added to the revised manuscript supplementary data (**Figure EV7** of Revised Manuscript).

Figure for reviewers removed.

2nd Revision - Editorial Decision 21st May 2026

Dear Dr Enriquez,

Thank you again for sending us your amended manuscript (EMBOJ-2024-119185R1) for reconsideration by The EMBO Journal, together with a point-by-point response to the issues raised during peer-review. Please accept my apologies for the unusual protraction in getting back to you due to delayed expert input and detailed discussion in the editorial team. Your revised study was sent back to the referees for their scientific reassessment, and we have received detailed re-reports from all of them, which I enclose below. As you will see, the reviewers state that the work has been reasonably enhanced by the revisions and they can now support publication at the EMBO Journal, pending minor revision.

Thus, we are pleased to inform you that your manuscript has been accepted in principle for publication in The EMBO Journal.

Please address the remaining remarks of experts #1 and #2's by complementing data annotation and amending the manuscript text - discussion of the results and related literature - introducing caveats where appropriate.

As to the remaining critique raised by referee #2 on mechanistic depth, we carefully considered this input, but concluded that in light of your NAC rescue experiment added during revision, which solidifies the functional connection of OMA1 to oxidative metabolism in the current setting, we do not require additional mechanistic insights for publication at our venue at this time.

We do now need you to take care of a number of minor issues related to formatting and data annotation, as detailed below.

Please submit a revised version of the manuscript using the link enclosed below.

As you might remember from previous experience, every paper at the EMBO Journal includes a 'Synopsis', displayed on the html and freely accessible to all readers. The synopsis includes a 'model' figure as well as 2-5 one-short-sentence bullet points that summarize the article. I would appreciate if you could provide this figure and the bullet points.

Thank you again for giving us the chance to consider your manuscript for The EMBO Journal, I look forward to hearing from you and receiving your final revised version of the manuscript.

Best regards,

Daniel Klimmeck

Daniel Klimmeck PhD
Senior Editor
The EMBO Journal

Formatting changes required for the revised version of the manuscript:

>> Author Contributions: Remove the author contributions information from the manuscript text. Note that CRediT has replaced the traditional author contributions section as of now because it offers a systematic machine-readable author contributions format that allows for more effective research assessment. and use the free text boxes beneath each contributing author's name to add specific details on the author's contribution.

More information is available in our guide to authors.
https://www.embopress.org/page/journal/14602075/authorguide

>> Author information: please revisit discrepancies on author information provided in our system vs the manuscript text for co-author J.A.E., E.R.M., J.V. .

>> Update the order and headings of the sections in the manuscript text to: Abstract / Introduction / Results / Discussion / Methods / Data Availability / Acknowledgements / Disclosure and Competing Interests Statement / References / Figure Legends

/ Expanded View Figure Legends

>> Adjust the title of the 'Competing Interests' section to 'Disclosure and Competing Interests Statement'.

>> Funding: merge with Ascknowledements; Y2020/BIO-6350, PRE2018-083478 and MCIN/AEI /10.13039/501100011033 are missing in our system, please add.

>> Figure callouts: there is a callouts for a Fig 2I but no such figure - should this be Fig 3I? missing for Fig 5D, Fig 7G.

>> Data availability section: remove the referee tokens and make sure the GEO and PRIDE datasets are made publicly accessible.

>> Please provide a complete set of source data files. Currently, Fig 4G is missing; the uploaded folder is empty, authors please check the labeling of Fig 7J and K.

>> Consider additional changes and comments from our production team as indicated below:

- Figure legends:
1. Please note that the figure EV4 D is mislabeled as figure EV4 C in the manuscript. This needs to be rectified.
2. Please note that the exact p values are not provided in the legends of figures 1A, B; 2A, 3A, B, C, D, E, F, H, I; 5D, F, G; 6B, C; 7B, C, E, F, G, H, J; EV1 B, D, D; EV3 H, I; EV5 E, F, G, I; EV7 B-I, K, L; EV9 B, F, H, I;
3. Please indicate the statistical test used for data analysis in the legends of figures 2E, F; 4A, B; 6B, C; 7B, C, E, F, G, H; EV1 H, EV3 B, D; EV4 B, C
4. Please note that the box plots need to be defined in terms of minima, maxima, centre, bounds of box and whiskers, and percentile in the legends of figures 6B, C; 7J
5. Please note that information related to n is missing in the legends of figures 1A, B; 7J, EV2 A, B, D; EV3 H, I; EV4 A, EV7 D-I, K, L
6. Please note that the error bars are not defined in the legends of figures EV1 H, I; EV2 A, B, C, D, F; EV4 A, B, C, E; EV7 D-I, K, L; EV8 C, D, E; EV9 E-J.
7. Please note that the black arrow heads are not defined in the legend of figure 5A, EV2 G. This needs to be rectified.
8. Please note that the yellow arrow heads are not defined in the legend of figure 5A. This needs to be rectified.

Further information is available in our Guide For Authors: https://link.springer.com/journal/44318/submission-guidelines

----

Referee #1:

The authors have provided compelling additional evidence to satisfy the points raised by all the reviewers, including myself.The co-culture experiment was particularly informative regarding the impact on t cell exhaustion markers. Given the importance of age (and sex) in this study, I would encourage the authors to indicate the age and sex of the mice (or cells derived thereof i.e. EV6) in the figure legends and when relevant, in the main text. Following these changes, the study should now be published. I caution the other two reviewers to consider experimental requests in mouse models that would require considerable resources and 2-3 years of additional time, especially since this paper was origianlly submitted in solid form (in my opinion) in 2024. It's now mid-2026.

Referee #2:

In their revised manuscript, Marti-Mateos et al. have added a significant amount of new data and adequately addressed most of this reviewer's concerns. However, the main issue that remains is an insufficient mechanistic link between Oma1 and NRF2. The data provided fall short of establishing compelling evidence to support proposed connection through sustained H2O2/ROS

production. First, the evidence that Oma1 KO cells produce more ROS is thin. In fact, several studies have reported no increase in ROS production in Oma1 deficient cells. Second, the effects of NAC on NRF2 are complex and stochastic and should be interpreted with caution. Third, hydrogen peroxide is well known to be a poor NRF2 inducer due to its low reactivity with Keap1 and higher affinity to other response pathways such as NF-kB signaling pathway.

Addressing these concerns would make this study suitable for publication. Without a solid mechanistic link, the present manuscript offers a lot of somewhat superficial data and would be a better fit for more specialized forum.

Referee #3:

The authors reply do all my concerns

The authors addressed the remaining editorial issues.

Dear Dr Enriquez,

Thank you for submitting the revised version of your manuscript. I have now evaluated your amended manuscript and concluded that the remaining minor concerns have been sufficiently addressed.

I am thus pleased to inform you that your manuscript has been accepted for publication in the EMBO Journal.

Your manuscript will be processed for publication by EMBO Press. It will be copy edited and you will receive page proofs prior to publication. Please note that you will be contacted by Springer Nature Author Services to complete licensing and payment information.

Please note that it is The EMBO Journal policy for the transcript of the editorial process (containing referee reports and your response letter) to be published as an online supplement to each paper. If you do NOT want this, you will need to inform the Editorial Office via email immediately. More information is available here: https://www.embopress.org/transparent-process#Review_Process

You may qualify for financial assistance for your publication charges - either via a Springer Nature fully open access agreement or an EMBO initiative. Check your eligibility: https://www.embopress.org/page/journal/14602075/authorguide#chargesguide

Should you be planning a Press Release on your article, please notify the production team via this form in order to coordinate publication and release dates: https://forms.cloud.microsoft/e/eH4A8JVP6g

If you have any questions, please do not hesitate to contact the Editorial Office. Thank you for your contribution to The EMBO Journal.

Thank you again for this contribution to The EMBO Journal and congratulations on a successful publication! Please consider us again in the future for your most exciting work.

Best regards,

Daniel Klimmeck

Daniel Klimmeck, PhD
Senior Editor
The EMBO Journal
EMBO
Postfach 1022-40
Meyerhofstrasse 1
D-69117 Heidelberg
contact@embojournal.org

*****
Please note that it is The EMBO Journal policy for the transcript of the editorial process (containing referee reports and your response letters) to be published as an online supplement to each paper. If you should prefer removal of any referee-only figures included in the point-by-point response(s), e.g. because they may still be used for future publication or because they have been reproduced from published work by others, please do let us know immediately via response email.
More information is available here: https://link.springer.com/partners/embo-press/editorial-policies#Peer%20review

EMBO Press Author Checklist

Corresponding Author Name: Jose Antonio Enriquez	
Journal Submitted to: EMBO Jouranal	
Manuscript Number: EMBOJ-2024-119185R-Q	

USEFUL LINKS FOR COMPLETING THIS FORM
The EMBO Journal - Author Guidelines
EMBO Reports - Author Guidelines
Molecular Systems Biology - Author Guidelines
EMBO Molecular Medicine - Author Guidelines

Reporting Checklist for Life Science Articles (updated January
This checklist is adapted from Materials Design Analysis Reporting (MDAR) Checklist for Authors. MDAR establishes a minimum set of requirements in transparent reporting in the life sciences (see Statement of Task: 10.31222/osf.io/9sm4x). Please follow the journal's guidelines in preparing your
Please note that a copy of this checklist will be published alongside your article.

Abridged guidelines for figures
1. Data
The data shown in figures should satisfy the following conditions:
- the data were obtained and processed according to the field's best practice and are presented to reflect the results of the experiments in an accurate and unbiased manner.
- ideally, figure panels should include only measurements that are directly comparable to each other and obtained with the same assay.
- plots include clearly labeled error bars for independent experiments and sample sizes. Unless justified, error bars should not be shown for technical
- if n<5, the individual data points from each experiment should be plotted. Any statistical test employed should be justified.
- Source Data should be included to report the data underlying figures according to the guidelines set out in the authorship guidelines on Data

2. Captions
Each figure caption should contain the following information, for each panel where they are relevant:
- a specification of the experimental system investigated (eg cell line, species name).
- the assay(s) and method(s) used to carry out the reported observations and measurements.
- an explicit mention of the biological and chemical entity(ies) that are being measured.
- an explicit mention of the biological and chemical entity(ies) that are altered/varied/perturbed in a controlled manner.
- the exact sample size (n) for each experimental group/condition, given as a number, not a range;
- a description of the sample collection allowing the reader to understand whether the samples represent technical or biological replicates (including how many animals, litters, cultures, etc.).
- a statement of how many times the experiment shown was independently replicated in the laboratory.
- definitions of statistical methods and measures:
  - common tests, such as t-test (please specify whether paired vs. unpaired), simple χ2 tests, Wilcoxon and Mann-Whitney tests, can be unambiguously identified by name only, but more complex techniques should be described in the methods section;
  - are tests one-sided or two-sided?
  - are there adjustments for multiple comparisons?
  - exact statistical test results, e.g., P values = x but not P values < x;
  - definition of 'center values' as median or average;
  - definition of error bars as s.d. or s.e.m.

Please complete ALL of the questions below.
Select "Not Applicable" only when the requested information is not relevant for your study.

Materials

Newly Created Materials	Information included in the manuscript?	In which section is the information available? (Reagents and Tools Table, Materials and Methods, Figures, Data Availability Section)
New materials and reagents need to be available; do any restrictions apply?	Not Applicable	

Antibodies	Information included in the manuscript?	In which section is the information available? (Reagents and Tools Table, Materials and Methods, Figures, Data Availability Section)
For **antibodies** provide the following information:<br>- Commercial antibodies: RRID (if possible) or supplier name, catalogue number and or/clone number<br>- Non-commercial: RRID or citation	Yes	Reagents and Tools Table

DNA and RNA sequences	Information included in the manuscript?	In which section is the information available? (Reagents and Tools Table, Materials and Methods, Figures, Data Availability Section)
**Short novel DNA or RNA including primers, probes**: provide the sequences.	Not Applicable	

Cell materials	Information included in the manuscript?	In which section is the information available? (Reagents and Tools Table, Materials and Methods, Figures, Data Availability Section)
**Cell lines:** Provide species information, strain. Provide accession number in repository **OR** supplier name, catalog number, clone number, and/**OR** RRID.	Not Applicable	
**Primary cultures:** Provide species, strain, sex of origin, genetic modification status.	Yes	Materials and Methods
Report if the cell lines were recently **authenticated** (e.g., by STR profiling) and tested for mycoplasma contamination.	Not Applicable	

Experimental animals	Information included in the manuscript?	In which section is the information available? (Reagents and Tools Table, Materials and Methods, Figures, Data Availability Section)
**Laboratory animals or Model organisms:** Provide species, strain, sex, age, genetic modification status. Provide accession number in repository **OR** supplier name, catalog number, clone number, **OR** RRID.	Yes	Reagents and Tools Table
**Animal observed in or captured from the field:** Provide species, sex, and age where possible.	Not Applicable	
Please detail **housing and husbandry conditions**.	Yes	Material and Methods

Plants and microbes	Information included in the manuscript?	In which section is the information available? (Reagents and Tools Table, Materials and Methods, Figures, Data Availability Section)
**Plants:** provide species and strain, ecotype and cultivar where relevant, unique accession number if available, and source (including location for collected wild specimens).	Not Applicable	
**Microbes:** provide species and strain, unique accession number if available, and source.	Not Applicable	

Human research participants	Information included in the manuscript?	In which section is the information available? (Reagents and Tools Table, Materials and Methods, Figures, Data Availability Section)
If collected and within the bounds of privacy constraints report on age, sex and gender or ethnicity for all study participants.	Not Applicable	

Core facilities	Information included in the manuscript?	In which section is the information available? (Reagents and Tools Table, Materials and Methods, Figures, Data Availability Section)
If your work benefited from core facilities, was their service mentioned in the acknowledgments section?	Yes	Material and Methods

Design

Study protocol	Information included in the manuscript?	In which section is the information available? (Reagents and Tools Table, Materials and Methods, Figures, Data Availability Section)
If study protocol has been **pre-registered, provide DOI in the manuscript**. For clinical trials, provide the trial registration number **OR** cite DOI.	Not Applicable	
Report the **clinical trial registration number** (at ClinicalTrials.gov or equivalent), where applicable.	Not Applicable	

Laboratory protocol	Information included in the manuscript?	In which section is the information available? (Reagents and Tools Table, Materials and Methods, Figures, Data Availability Section)
Provide DOI OR other citation details if **external detailed step-by-step protocols** are available.	Not Applicable	

Experimental study design and statistics	Information included in the manuscript?	In which section is the information available? (Reagents and Tools Table, Materials and Methods, Figures, Data Availability Section)
Include a statement about **sample size** estimate even if no statistical methods were used.	Yes	Material and Methods
Were any steps taken to minimize the effects of subjective bias when allocating animals/samples to treatment (e.g. **randomization procedure**)? If yes, have they been described?	Yes	Material and Methods
Include a statement about **blinding** even if no blinding was done.	Yes	Material and Methods
Describe **inclusion/exclusion criteria** if samples or animals were excluded from the analysis. Were the criteria pre-established?<br><br>If sample or data points were omitted from analysis, report if this was due to attrition or intentional exclusion and provide justification.	Yes	Material and Methods
For every figure, are **statistical tests** justified as appropriate? Do the data meet the assumptions of the tests (e.g., normal distribution)? Describe any methods used to assess it. Is there an estimate of variation within each group of data? Is the variance similar between the groups that are being statistically compared?	Yes	Figures

Sample definition and in-laboratory replication	Information included in the manuscript?	In which section is the information available? (Reagents and Tools Table, Materials and Methods, Figures, Data Availability Section)
In the figure legends: state number of times the experiment was **replicated** in laboratory.	Yes	Figures
In the figure legends: define whether data describe **technical or biological replicates**.	Yes	Figures

Ethics

Ethics	Information included in the manuscript?	In which section is the information available? (Reagents and Tools Table, Materials and Methods, Figures, Data Availability Section)
Studies involving **human participants**: State details of **authority granting ethics approval** (IRB or equivalent committee(s), provide reference number for approval.	Not Applicable	
Studies involving **human participants**: Include a statement confirming that **informed consent** was obtained from all subjects and that the experiments conformed to the principles set out in the WMA Declaration of Helsinki and the Department of Health and Human Services Belmont Report.	Not Applicable	
Studies involving **human participants: For** publication of **patient photos**, include a statement confirming that consent to publish was obtained.	Not Applicable	
Studies involving experimental **animals**: State details of **authority granting ethics approval** (IRB or equivalent committee(s), provide reference number for approval. Include a statement of compliance with ethical regulations.	Yes	Material and Methods
Studies involving **specimen and field samples:** State if relevant **permits** obtained, provide details of authority approving study; if none were required, explain why.	Not Applicable	

Dual Use Research of Concern (DURC)	Information included in the manuscript?	In which section is the information available? (Reagents and Tools Table, Materials and Methods, Figures, Data Availability Section)
Could your study fall under dual use research restrictions? Please check biosecurity documents and list of **select agents and toxins** (CDC): https://www.selectagents.gov/sat/list.htm	Not Applicable	
If you used a select agent, is the security level of the lab appropriate and reported in the manuscript?	Not Applicable	
If a study is subject to dual use research of concern regulations, is the name of the **authority granting approval and reference number** for the regulatory approval provided in the manuscript?	Not Applicable	

Reporting

The MDAR framework recommends adoption of discipline-specific guidelines, established and endorsed through community initiatives. Journals have their own policy about requiring specific guidelines and recommendations to complement MDAR.

Adherence to community standards	Information included in the manuscript?	In which section is the information available? (Reagents and Tools Table, Materials and Methods, Figures, Data Availability Section)
State if relevant guidelines or checklists (e.g., **ICMJE, MIBBI, ARRIVE, PRISMA**) have been followed or provided.	Not Applicable	
For **tumor marker prognostic studies,** we recommend that you follow the **REMARK** reporting guidelines (see link list at top right). See author guidelines, under 'Reporting Guidelines'. Please confirm you have followed these guidelines.	Not Applicable	
For **phase II and III randomized controlled trials**, please refer to the **CONSORT** flow diagram (see link list at top right) and submit the CONSORT checklist (see link list at top right) with your submission. See author guidelines, under 'Reporting Guidelines'. Please confirm you have submitted this list.	Not Applicable	

Data Availability

Data availability	Information included in the manuscript?	In which section is the information available? (Reagents and Tools Table, Materials and Methods, Figures, Data Availability Section)
Have **primary datasets** been deposited according to the journal's guidelines (see 'Data Deposition' section) and the respective accession numbers provided in the Data Availability Section?	Yes	Data Availability Section
Were **human clinical and genomic datasets** deposited in a public access-controlled repository in accordance to ethical obligations to the patients and to the applicable consent agreement?	Not Applicable	
Are **computational models** that are central and integral to a study available without restrictions in a machine-readable form? Were the relevant accession numbers or links provided?	Not Applicable	
If publicly available data were reused, provide the respective **data citations in the reference list**.	Yes	Material and Methods
