# Supplementary material for: OMA1 protects from liver injury and tumorigenesis during aging by controlling hepatic immunogenicity
Source: EMBO J. 2026 Jun 23;45(15):5292–329. doi: 10.1038/s44318-026-00839-4 (PMC13434532; doi:10.1038/s44318-026-00839-4)
Supplement: Supplementary file 2 [file 44318_2026_839_MOESM9_ESM.pdf]

Expanded View Figures

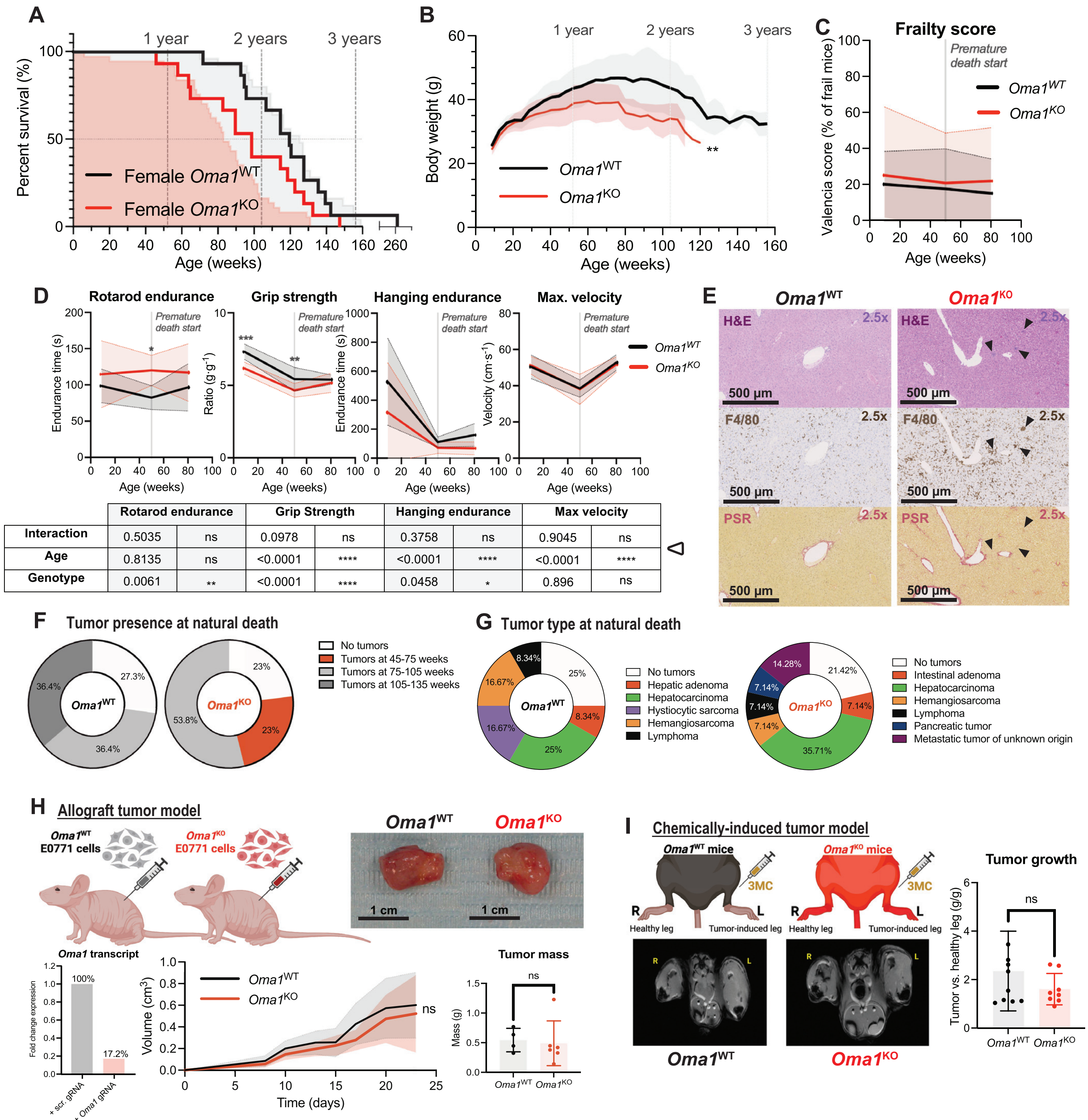

	Rotarod endurance		Grip Strength		Hanging endurance		Max velocity	
Interaction	0.5035	ns	0.0978	ns	0.3758	ns	0.9045	ns
Age	0.8135	ns	<0.0001	****	<0.0001	****	<0.0001	****
Genotype	0.0061	**	<0.0001	****	0.0458	*	0.896	ns

 © The Author(s)

Figure EV1. Decreased survival and increased hepatic primary tumor incidence in *Oma1*^{KO} mice.

(**A**) Survival analysis of female *Oma1*^{WT} ($n = 15$) and *Oma1*^{KO} ($n = 15$) mice in an inbred C57BL6/JOlaHsd background. (**B**) Body weight evolution ($P = 0.0012$) of male *Oma1*^{WT} ($n = 25$) and *Oma1*^{KO} ($n = 37$) mice in an inbred C57BL6/JOlaHsd background. This graph contains data from two separate experiments. *P* value from a Mixed-effects model testing the "OMA1 expression" fixed effect is represented in the graph. (**C**) Frailty score from male *Oma1*^{WT} and *Oma1*^{KO} mice at 10, 50, and 80 weeks of age, including rotarod endurance, grip strength, hanging endurance, and maximum velocity. Mice cohorts at the analyzed time points were not the same individuals. Graphical representation using continuous lines has been chosen for visual simplicity. The number of animals used at these 10, 50, and 80 weeks of age cohorts were *Oma1*^{WT}: $n = 10$, $n = 10$, and $n = 5$, respectively; and *Oma1*^{KO}: $n = 10$, $n = 12$, and $n = 8$, respectively. (**D**) Performance evolution at individual functional tests using male *Oma1*^{WT} and *Oma1*^{KO} mice at 10, 50, and 80 weeks of age, including rotarod endurance (50w $P = 0.0187$), grip strength (9w $P = 0.0056$, 50w $P < 0.0001$), hanging endurance, and maximum velocity. Mice cohorts at the analyzed time points were not the same individuals. Graphical representation using continuous lines has been chosen for visual simplicity. The number of animals used at these 10, 50, and 80 weeks of age cohorts were *Oma1*^{WT}: $n = 10$, $n = 10$, and $n = 5$, respectively; and *Oma1*^{KO}: $n = 10$, $n = 12$, and $n = 8$, respectively. *P* values from two-way ANOVA tests assessing "Age", "Genotype", and "Interaction" as possible sources of variation are indicated below. (**E**) Representative H&E, F4/80, and PSR histological analysis from a male *Oma1*^{KO} mouse autopsy presenting liver damage at 58 weeks of age compared to a representative 60-week-old *Oma1*^{WT} liver. Arrows indicate multifocal liver microgranuloma lesions. (**F**) Percentage of analyzed *Oma1*^{WT} ($n = 11$) and *Oma1*^{KO} ($n = 13$) carcasses presenting tumors in survival experiments subdivided by their age at demise. (**G**) Tumor type percentages in analyzed *Oma1*^{WT} ($n = 11$) and *Oma1*^{KO} ($n = 13$) carcasses presenting tumors in survival experiments. (**H**) Allograft tumor model, depicting: the experimental design, consisting of the injection of *Oma1*^{WT} and *Oma1*^{KO} E0771 lines generated by CRISPR-Cas9 technology into *nude* mice (above, left); tumor volume growth estimation of *Oma1*^{WT} and *Oma1*^{KO} allografts (mean ± SD) (below, left); representative images of *Oma1*^{WT} and *Oma1*^{KO} allografts at sacrifice (above, right); and final tumor dimensions (volume and mass) of *Oma1*^{WT} and *Oma1*^{KO} allografts at sacrifice (below, right). "Tumor volume" and "Tumor mass" values did not pass normality. *P* values from Mann–Whitney tests are represented in each graph. (**I**) Chemically induced tumor model, depicting: the experimental design, consisting of the intramuscular injection of 3MC in male constitutive *Oma1*^{WT} and *Oma1*^{KO} mice (above, left); representative MRI images from *Oma1*^{WT} ($n = 10$) and *Oma1*^{KO} ($n = 9$) mice after 3 months of 3MC-induction (R = right, L = left) (below, left); and final tumor-induced to healthy leg mass from *Oma1*^{WT} and *Oma1*^{KO} mice after 3 months of 3MC-induction (mean ± SD) (right). "Tumor growth" values passed normality. *P* value from an unpaired two-tailed Student's *t* test is represented in the graph. ns $P > 0.05$, $^{*}P < 0.05$, $^{**}P < 0.01$, $^{***}P < 0.001$, and $^{****}P < 0.0001$.

© The Author(s)

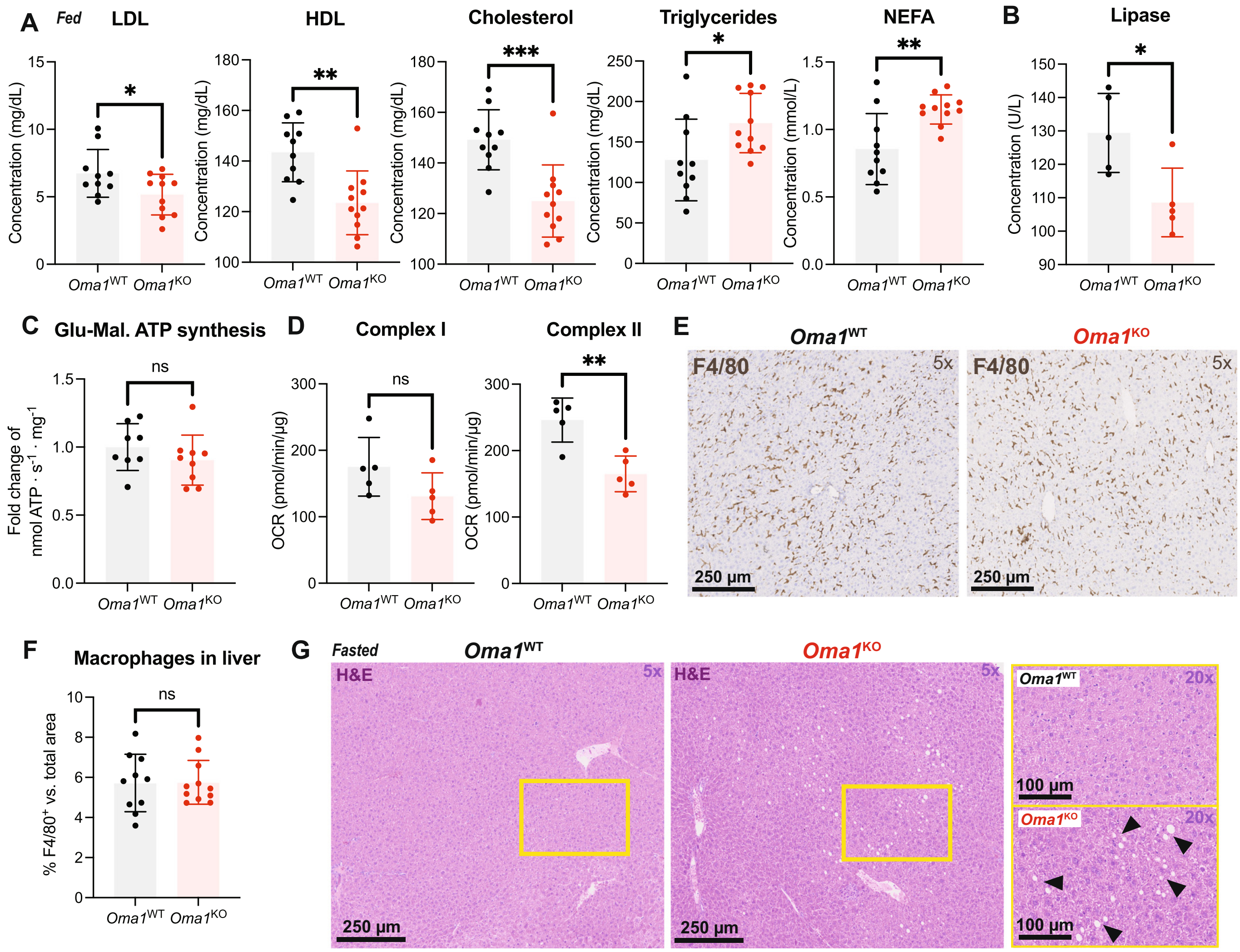

Figure EV2. Early liver dysfunction in young *Oma1*KO mice.

(**A**) Fed-state values of plasma LDL ($P = 0.0410$), HDL ($P = 0.0014$), total cholesterol ($P = 0005$), NEFAs ($P = 0.0029$), and TAGs ($P = 0.0272$) in 10-week-old male *Oma1*WT ($n = 10$) and *Oma1*KO ($n = 11$) mice (mean ± SD). (**B**) Plasma lipase ($P = 0.0180$) in 10-week-old male *Oma1*WT ($n = 5$) and *Oma1*KO ($n = 5$) mice. (**C**) ATP synthesis production using glutamate-malate in liver isolated mitochondria from 10-week-old male mice fed *Oma1*WT ($n = 8$) and *Oma1*KO ($n = 9$) mice (mean ± SD). (**D**) Basal oxygen consumption rate from complex I and complex II ($P = 0.0028$) substrates in frozen isolated mitochondria from male *Oma1*WT ($n = 5$) and *Oma1*KO ($n = 5$) livers (left). Complex II-driven OCR seahorse profile (right) (mean ± SD). (**E**) Representative F4/80 staining analysis of 10-week-old male fed *Oma1*WT ($n = 10$) and *Oma1*KO ($n = 11$) paraffin liver sections. (**F**) F4/80 staining analysis of 10-week-old male fed *Oma1*WT ($n = 10$) and *Oma1*KO ($n = 11$) paraffin liver sections (mean ± SD). (**G**) Representative H&E staining analysis of 6-h fasted 10-week-old male *Oma1*WT ($n = 10$) and *Oma1*KO ($n = 11$) paraffin liver sections. Magnifications are depicted to the right to better visualize lipid droplets (black arrows) in fasted livers. *P* value from unpaired two-tailed Student's *t* test (when data passed normality) or Mann–Whitney test (when data did not pass normality) is represented in each graph. ns $P > 0.05$, $^{*}P < 0.05$, $^{**}P < 0.01$, $^{***}P < 0.001$, and $^{****}P < 0.0001$.

 © The Author(s)

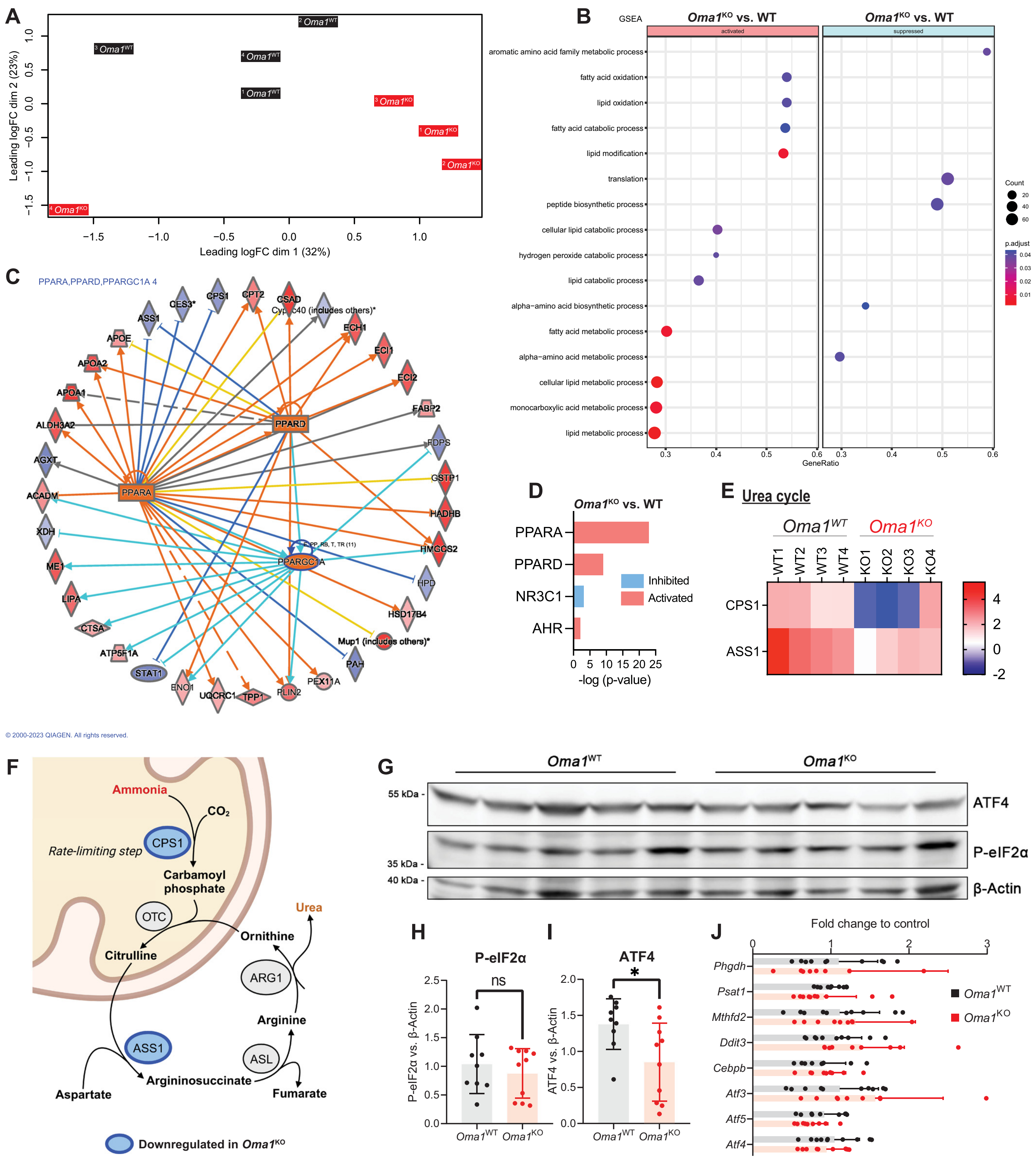
A
Leading logFC dim 1 (32%)
Leading logFC dim 2 (23%)
B
GSEA
Oma1KO vs. WT
activated
suppressed
aromatic amino acid family metabolic process
fatty acid oxidation
lipid oxidation
fatty acid catabolic process
lipid modification
translation
peptide biosynthetic process
cellular lipid catabolic process
hydrogen peroxide catabolic process
lipid catabolic process
alpha-amino acid biosynthetic process
fatty acid metabolic process
alpha-amino acid metabolic process
cellular lipid metabolic process
monocarboxylic acid metabolic process
lipid metabolic process
GeneRatio
Count
p.adjust
C
PPARA,PPARD,PPARGC1A 4
© 2000-2023 QIAGEN. All rights reserved.
D
Oma1KO vs. WT
PPARA
PPARD
NR3C1
AHR
Inhibited
Activated
-log (p-value)
E
Urea cycle
Oma1WT
Oma1KO
CPS1
ASS1
F
Ammonia
CO2
CPS1
Rate-limiting step
Carbamoyl phosphate
OTC
Citrulline
Ornithine
Urea
ARG1
Arginine
ASS1
ASL
Aspartate
Argininosuccinate
Fumarate
Downregulated in Oma1KO
G
Oma1WT
Oma1KO
55 kDa
35 kDa
40 kDa
ATF4
P-eIF2α
β-Actin
H
P-eIF2α
ns
P-eIF2α vs. β-Actin
I
ATF4
ATF4 vs. β-Actin
J
Fold change to control
Phgdh
Psat1
Mthfd2
Ddit3
Cebpb
Atf3
Atf5
Atf4

© The Author(s)

Figure EV3. Increased oxidative stress and liver degeneration in young *Oma1*^{KO} livers.

(**A**) Principal Component Analysis using proteomics data from 9-week-old *Oma1*^{WT} ($n = 4$) and *Oma1*^{KO} ($n = 4$) liver samples. (**B**) Gene Set Enrichment Analysis using proteomics data from 9-week-old male *Oma1*^{WT} ($n = 4$) and *Oma1*^{KO} ($n = 4$) liver samples. Kolmogorov–Smirnov test (one-sided) and Benjamini–Hochberg method, adjusted P value < 0.05. (**C**) Gene networks under the control of the predicted upstream regulators PPARA, PPARD, and PPARGC1A according to IPA analysis using proteomics data from 9-week-old male *Oma1*^{KO} ($n = 4$) liver proteomics samples compared to *Oma1*^{WT} ($n = 4$) mice. (**D**) IPA-obtained upstream regulators in young 9-week-old male *Oma1*^{KO} ($n = 4$) mice compared to *Oma1*^{WT} ($n = 4$) animals filtered by ligand-dependent nuclear receptors. Benjamini–Hochberg method, adjusted P value < 0.05. (**E**) Proteomics heatmap of urea cycle gene expression in young 9-week-old male *Oma1*^{KO} ($n = 4$) mice compared to *Oma1*^{WT} ($n = 4$) animals. (**F**) Simplified representation of the urea cycle showing the downregulated proteins according to proteomics in 9-week-old male *Oma1*^{KO} ($n = 4$) mice compared to *Oma1*^{WT} ($n = 4$) animals. (**G**) Representative western blot analysis of ATF4 and P-eIF2α protein signal from young 10 weeks-old male *Oma1*^{WT} ($n = 5$) and *Oma1*^{KO} ($n = 5$) homogenate liver samples, using β-Actin as a loading control. (**H**) Quantification of relative P-eIF2α to β-Actin protein signal ($P = 0.7802$) according to Western Blot analyses from young 10-week-old male *Oma1*^{WT} ($n = 9$) and *Oma1*^{KO} ($n = 10$) homogenate liver samples. The data did not pass normality. P value from the Mann–Whitney test is represented in the graph. (**I**) Quantification of relative ATF4 to β-Actin protein signal ($P = 0.0223$) according to western blot analyses from young 10-week-old *Oma1*^{WT} ($n = 9$) and *Oma1*^{KO} ($n = 10$) homogenate liver samples. Data passed normality. P value from Welch's t test (SDs differed) is represented in the graph. (**J**) Relative expression of *Atf4* and *Atf4*-dependent transcriptional targets in young 10 weeks-old *Oma1*^{WT} ($n = 10$) and *Oma1*^{KO} ($n = 10$) liver samples. ns $P > 0.05$, $^{*}P < 0.05$, $^{**}P < 0.01$, $^{***}P < 0.001$, and $^{****}P < 0.0001$.

© The Author(s)

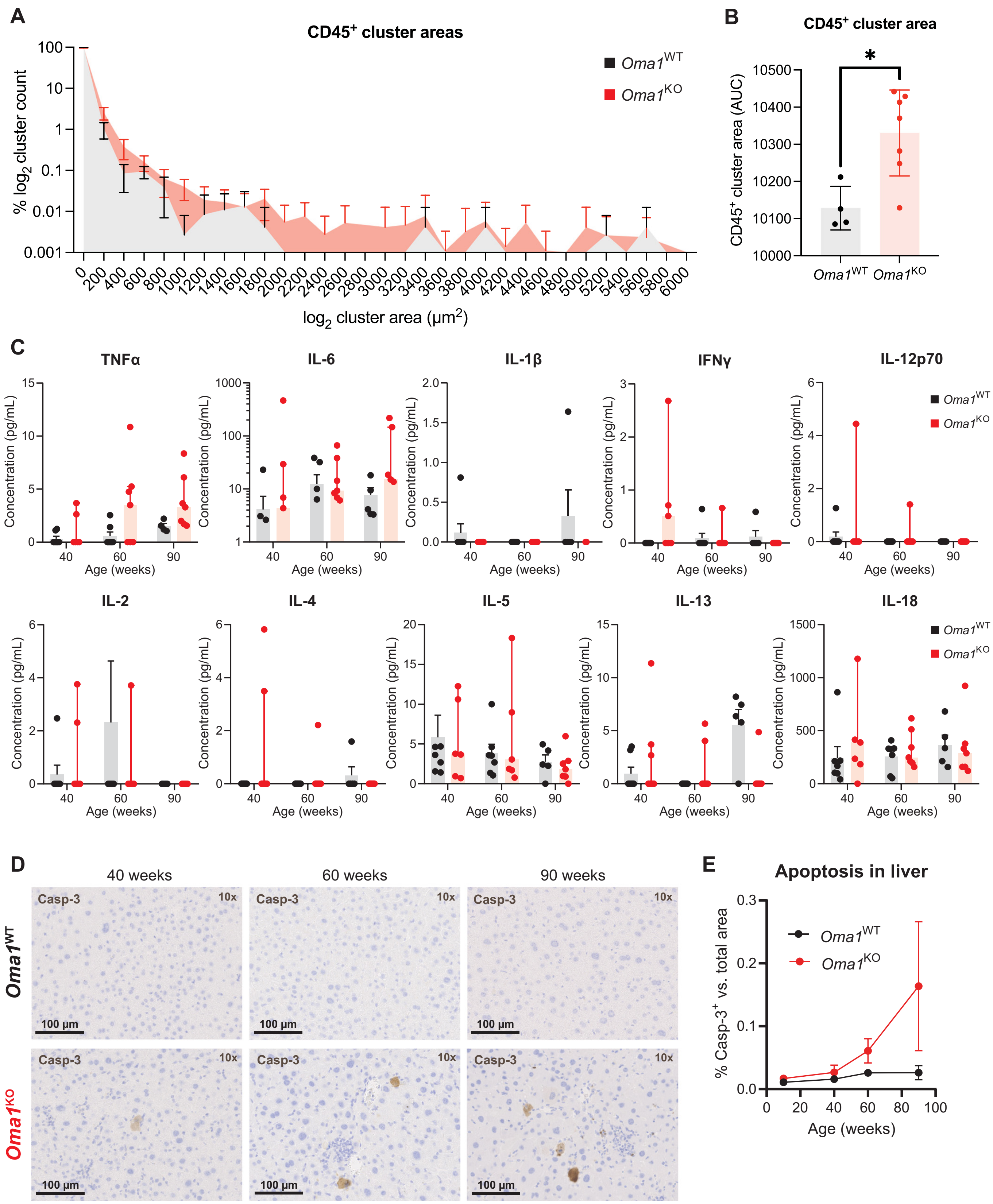
A
CD45+ cluster areas
Oma1WT
Oma1KO
% log2 cluster count
100
10
1
0.1
0.01
0.001
log2 cluster area (μm2)
0 200 400 600 800 1000 1200 1400 1600 1800 2000 2200 2400 2600 2800 3000 3200 3400 3600 3800 4000 4200 4400 4600 4800 5000 5200 5400 5600 5800 6000
B
CD45+ cluster area
*
CD45+ cluster area (AUC)
10500
10400
10300
10200
10100
10000
Oma1WT
Oma1KO
C
TNFα
IL-6
IL-1β
IFNγ
IL-12p70
IL-2
IL-4
IL-5
IL-13
IL-18
Concentration (pg/mL)
Age (weeks)
40
60
90
Oma1WT
Oma1KO
D
40 weeks
60 weeks
90 weeks
Oma1WT
Oma1KO
Casp-3
10x
100 μm
E
Apoptosis in liver
Oma1WT
Oma1KO
% Casp-3+ vs. total area
0.3
0.2
0.1
0.0
Age (weeks)
0 20 40 60 80 100

Figure EV4. Chronic liver inflammation and fibrosis in *Oma1*^{KO} mice.

(**A**) CD45$^+$ cluster areas histogram from 90-week-old male *Oma1*^{WT} ($n = 4$) and *Oma1*^{KO} ($n = 7$) immunofluorescence liver images (mean ± SD). (**B**) Area under the curve (AUC) from CD45$^+$ cluster areas histograms using 90-week-old male *Oma1*^{WT} ($n = 4$) and *Oma1*^{KO} ($n = 7$) immunofluorescence liver images (mean ± SD). Data passed normality, *P* value from unpaired two-tailed Student's *t* test is represented in the graph. (**C**) Plasma cytokine values from 40, 60, and 90-week-old male *Oma1*^{WT} and *Oma1*^{KO} mice. Mice cohorts at the analyzed time points were not the same individuals. The number of animals used at these 40, 60, and 90 weeks of age cohorts were *Oma1*^{WT}: $n = 7$, $n = 7$, and $n = 5$, respectively; and *Oma1*^{KO}: $n = 7$, $n = 7$, and $n = 7$, respectively (mean ± SD). Two-way ANOVA. (**D**) Representative Caspase-3 staining analysis of 10, 40, 60, and 90-week-old male *Oma1*^{WT} and *Oma1*^{KO} tumor-free liver tissue. (**E**) Relative Caspase-3-stained area to whole liver tissue area quantification from 10, 40, 60, and 90-week-old *Oma1*^{WT} and *Oma1*^{KO} livers. Mice cohorts at the analyzed time points were not the same individuals. Graphical representation using continuous lines has been chosen for visual simplicity. The number of animals used at these 10, 40, 60, and 90 weeks of age cohorts were *Oma1*^{WT}: $n = 5$, $n = 5$, $n = 5$, and $n = 4$, respectively; and *Oma1*^{KO}: $n = 6$, $n = 4$, $n = 4$, and $n = 7$, respectively (mean ± SD). *P* value from a two-way ANOVA testing "OMA1 expression" as a source of variation is represented in the graph. ns $P > 0.05$, $^{*}P < 0.05$, $^{**}P < 0.01$, $^{***}P < 0.001$, and $^{****}P < 0.0001$.

 © The Author(s)

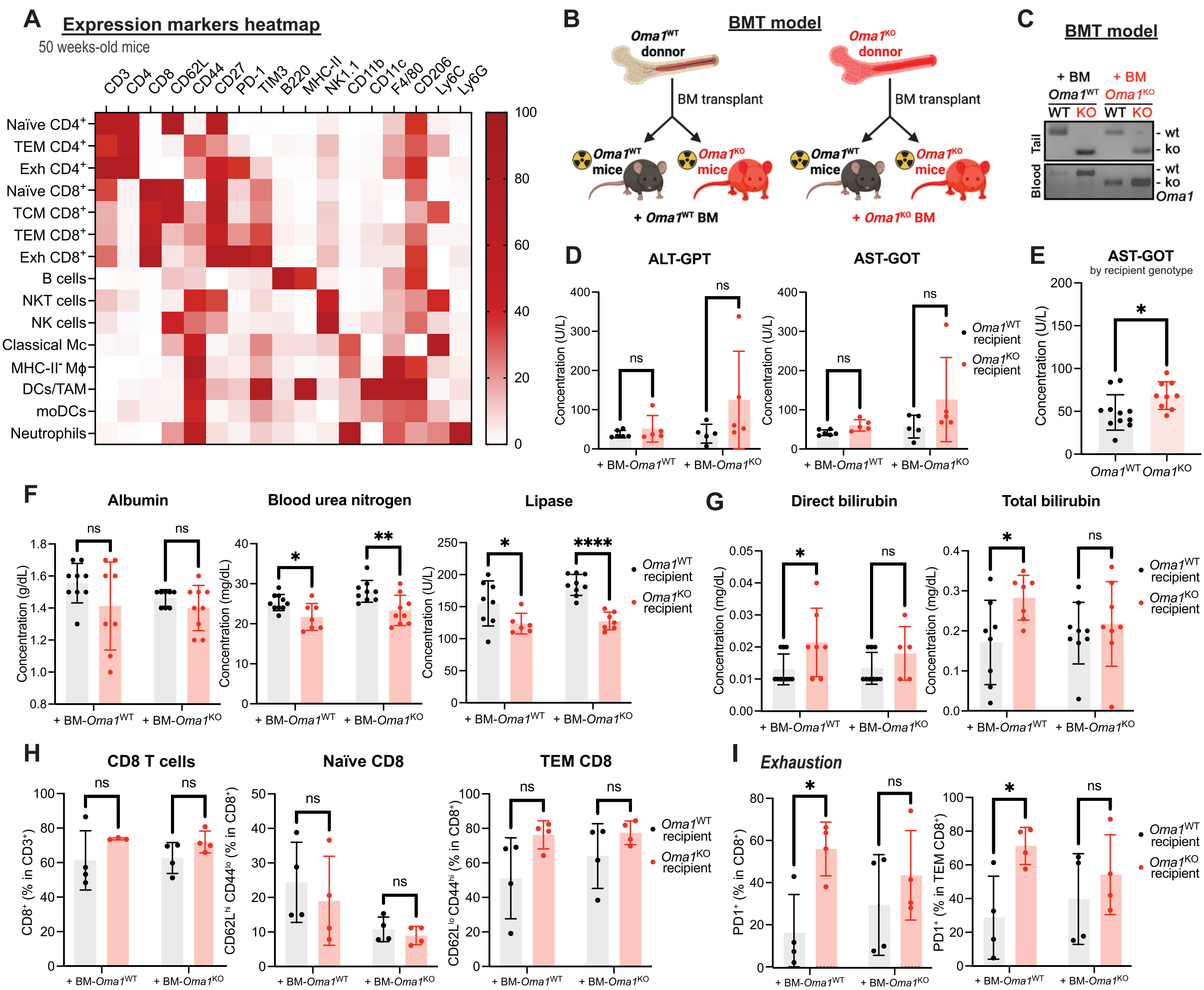

Figure EV5. Progressive immune exhaustion in *Oma1*^{KO} livers.

(**A**) Expression markers heatmap from 50-week-old male *Oma1*^{WT} ($n = 4$) and *Oma1*^{KO} ($n = 5$) mice, assigning expression patterns to diverse immune populations using OMIQ. (**B**) "BMT model" experimental design scheme, depicting *Oma1*^{WT} and *Oma1*^{KO} bone marrow transfer to male *Oma1*^{WT} ($n = 10$ and $n = 9$, respectively) and *Oma1*^{KO} ($n = 9$ and $n = 9$, respectively) lethally irradiated mice. (**C**) *Oma1* genotype in tail and blood samples from male irradiated *Oma1*^{WT} and *Oma1*^{KO} mice transferred with *Oma1*^{WT} and *Oma1*^{KO} BM. (**D**) Plasma ALT-GPT and AST-GOT values from 40-week-old male irradiated *Oma1*^{WT} and *Oma1*^{KO} mice transferred with *Oma1*^{WT} ($n = 5$ and $n = 5$, respectively) and *Oma1*^{KO} ($n = 5$ and $n = 5$, respectively) BMs. *P* values from Šídák's multiple comparisons tests comparing *Oma1*^{WT} and *Oma1*^{KO} recipient animals in the presence of each bone marrow are represented in each graph. (**E**) Plasma AST-GOT values ($P = 0.0313$) from 40-week-old male irradiated *Oma1*^{WT} and *Oma1*^{KO} mice, independently of the bone marrow genotype (*Oma1*^{WT} or *Oma1*^{KO}) they were transferred with. "AST-GOT" values passed normality. (**F**) Plasma albumin, blood urea nitrogen (+BM-*Oma1*^{WT} $P = 0.0411$, +BM-*Oma1*^{KO} $P = 0.0038$) and lipase (+BM-*Oma1*^{WT} $P = 0.0306$, +BM-*Oma1*^{KO} $P < 0.0001$) values from 20 weeks-old male irradiated *Oma1*^{WT} and *Oma1*^{KO} mice transferred with *Oma1*^{WT} ($n = 10$ and $n = 9$, respectively) and *Oma1*^{KO} ($n = 9$ and $n = 9$, respectively) BMs. *P* values from Šídák's multiple comparisons tests comparing *Oma1*^{WT} and *Oma1*^{KO} recipient animals in the presence of each bone marrow are represented in each graph. (**G**) Plasma direct bilirubin (+BM-*Oma1*^{WT} $P = 0.0470$) and total bilirubin values (+BM-*Oma1*^{WT} $P = 0.0440$) from 20-week-old male irradiated *Oma1*^{WT} and *Oma1*^{KO} mice transferred with *Oma1*^{WT} ($n = 10$ and $n = 9$, respectively) and *Oma1*^{KO} ($n = 9$ and $n = 9$, respectively) BMs. *P* values from Šídák's multiple comparisons tests comparing *Oma1*^{WT} and *Oma1*^{KO} recipient animals in the presence of each bone marrow are represented in each graph. (**H**) Liver CD8$^+$ T cells populations from 20-week-old male irradiated *Oma1*^{WT} and *Oma1*^{KO} mice transferred with *Oma1*^{WT} ($n = 10$ and $n = 9$, respectively) and *Oma1*^{KO} ($n = 9$ and $n = 9$, respectively) BMs, including percentages of total, naive and TEM CD8$^+$ T cell populations. *P* values from Šídák's multiple comparisons tests comparing *Oma1*^{WT} and *Oma1*^{KO} recipient animals in the presence of each bone marrow are represented in each graph. (**I**) Percentages of exhausted (PD-1$^+$) liver CD8$^+$ T cells (+BM-*Oma1*^{WT} $P = 0.0267$) and TEM CD8$^+$ cells (+BM-*Oma1*^{WT} $P = 0.0393$) populations from 20 weeks-old male irradiated *Oma1*^{WT} and *Oma1*^{KO} mice transferred with *Oma1*^{WT} ($n = 10$ and $n = 9$, respectively) and *Oma1*^{KO} ($n = 9$ and $n = 9$, respectively) BMs. *P* values from Šídák's multiple comparisons tests comparing *Oma1*^{WT} and *Oma1*^{KO} animals within every cell population are represented in each graph. ns $P > 0.05$, $^*P < 0.05$, $^{**}P < 0.01$, $^{***}P < 0.001$, and $^{****}P < 0.0001$.

© The Author(s)

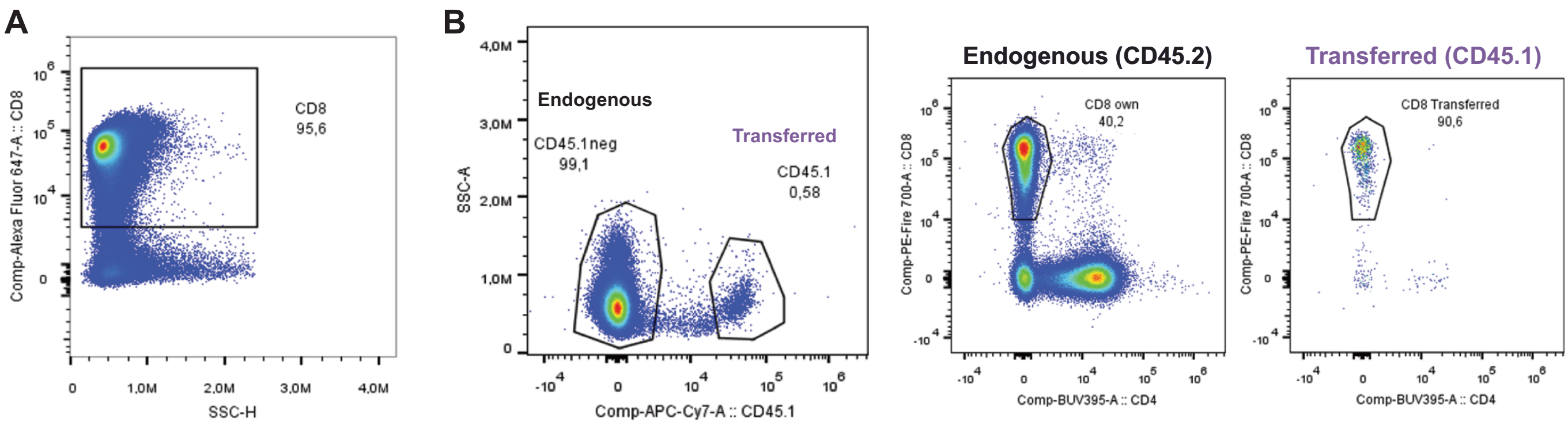

Figure EV6. Analysis of transferred and endogenous liver CD8$^+$ T cells populations.

(**A**) CD8$^+$ cells content in CD45.1 CD8$^+$ T cells extracts used for adoptive transfer. (**B**) Gating strategy to analyze separately CD45.2 (endogenous) from CD45.1 (transferred) liver CD8$^+$ T cells after 16 days of the adoptive transfer experiment. Cells were gated separately according to their CD45.1 level of expression (left). CD8$^+$ vs. CD4$^+$ expression pattern in CD45.1-negative cells (endogenous) and CD45.1-expressing cells (transferred) (right).

 © The Author(s)

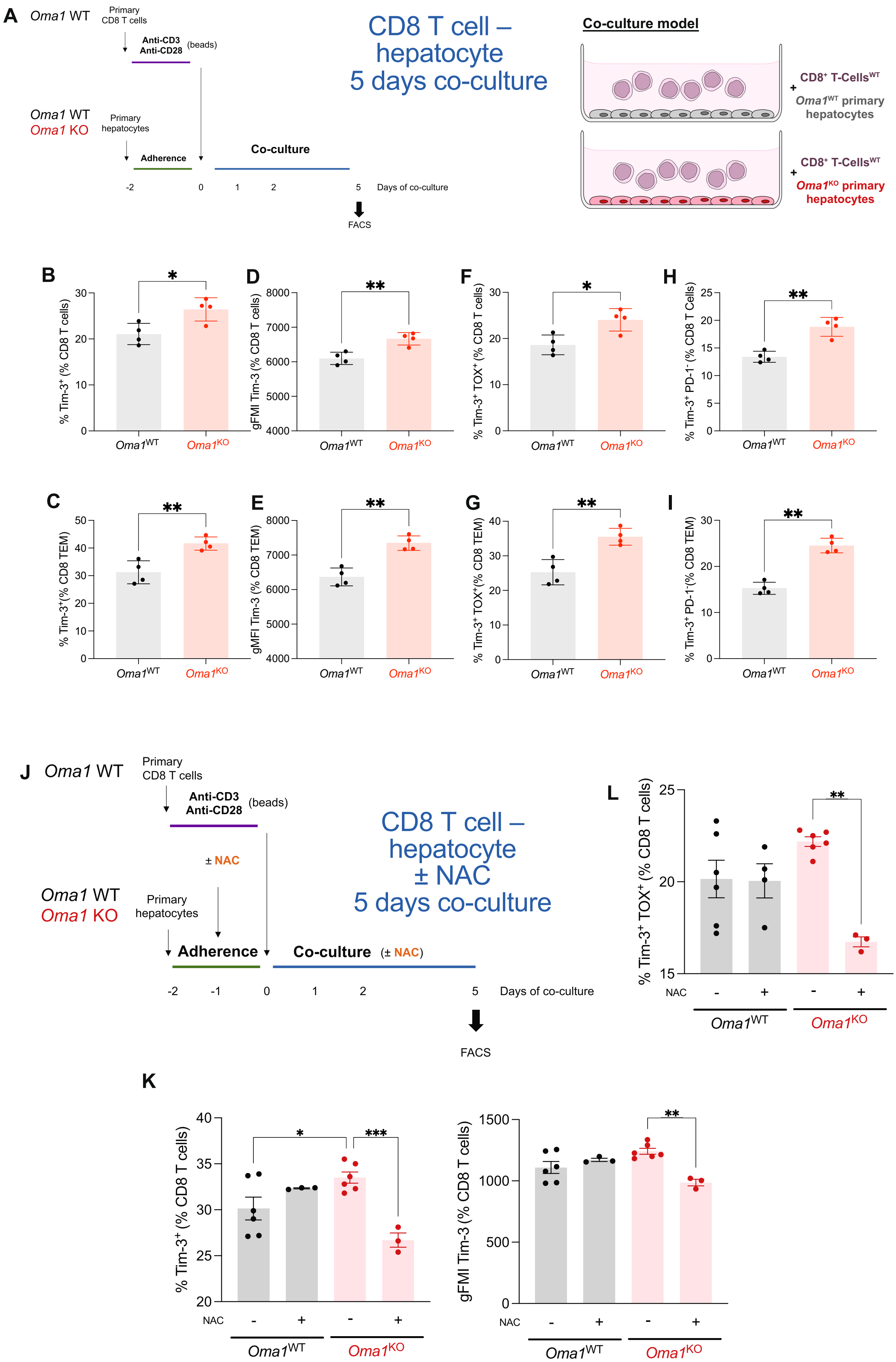
A
Oma1 WT
Primary CD8 T cells
Anti-CD3 Anti-CD28 (beads)
Oma1 WT
Oma1 KO
Primary hepatocytes
Adherence
Co-culture
Days of co-culture
FACS
CD8 T cell – hepatocyte 5 days co-culture
Co-culture model
CD8+ T-CellsWT + Oma1WT primary hepatocytes
CD8+ T-CellsWT + Oma1KO primary hepatocytes
B
% Tim-3+ (% CD8 T cells)
C
% Tim-3+(% CD8 TEM)
D
gFMI Tim-3 (% CD8 T cells)
E
gMFI Tim-3 (% CD8 TEM)
F
% Tim-3+ TOX+ (% CD8 T cells)
G
% Tim-3+ TOX+(% CD8 TEM)
H
% Tim-3+ PD-1- (% CD8 T Cells)
I
% Tim-3+ PD-1-(% CD8 TEM)
Oma1WT
Oma1KO
J
Oma1 WT
Primary CD8 T cells
Anti-CD3 Anti-CD28 (beads)
± NAC
Oma1 WT
Oma1 KO
Primary hepatocytes
Adherence
Co-culture (± NAC)
Days of co-culture
FACS
CD8 T cell – hepatocyte ± NAC 5 days co-culture
K
% Tim-3+ (% CD8 T cells)
gFMI Tim-3 (% CD8 T cells)
L
% Tim-3+ TOX+ (% CD8 T cells)
NAC
Oma1WT
Oma1KO

Figure EV7. Assessment of immunogenicity of *Oma1*^{KO} hepatocytes.

(**A**) Co-culture model: primary hepatocytes were isolated from 26-week-old male *Oma1*^{WT} ($n = 2$) and *Oma1*^{KO} ($n = 2$) mice and cultured for 48 h before adding naive or pre-activated (bead) CD8^{+} T cells. Exhaustion markers were measured after 5 days in co-culture. Technical replicates for (**B–I**) are: *Oma1*^{WT} ($n = 4$) and *Oma1*^{KO} ($n = 4$). (**B**, **C**) Overall prevalence of Tim-3 expression within total CD8^{+} T cells ($P = 0.0209$) (**B**) or within the effector-memory (TEM) CD8^{+} T cells ($P = 0.0083$) (**C**) (mean ± SD). (**D**, **E**) Same as (**B**, **C**) but assessed by geometric Mean Fluorescence Intensity (gMFI) (in total CD8^{+} T cells $P = 0.0043$, in TEM CD8 + T cells $P = 0.0012$) (mean ± SD). (**F**, **G**) Activated CD8^{+} T cell subpopulations (in total CD8^{+} T cells $P = 0.0159$, in TEM CD8 + T cells $P = 0.0049$) (mean ± SD). (**H**, **I**) Committed to exhaustion CD8^{+} T cell subpopulations (in total CD8^{+} T cells $P = 0.0030$, in TEM CD8 + T cells $P = 0.0001$). (**J**) Co-culture model: primary hepatocytes were isolated from 26-week-old male Oma1^{WT} ($n = 2$) and Oma1^{KO} ($n = 2$) mice and cultured for 5 days in the presence of the antioxidant NAC before adding naive CD8^{+} T cells. Technical replicates for (**K**, **L**) were: *Oma1*^{WT} – NAC ($n = 6$), *Oma1*^{WT} + NAC ($n = 4$), *Oma1*^{KO} – NAC ($n = 6$), *Oma1*^{KO} + NAC ($n = 3$). (**K**) Overall prevalence of Tim-3 expression within total CD8^{+} T cells in the presence or absence of NAC (%Tim-3: KO – vs. + NAC $P = 0.0024$, NAC WT vs. KO $P = 0.0255$; gFMI Tim-3: KO – vs. + NAC $P = 0.0038$) (mean ± SD). (**L**) Committed to exhaustion CD8+ T cell subpopulations in the presence of absence of NAC ($P = 0.0026$). *P* value from ordinary one-way ANOVA test post-hoc Multiple comparisons Sidak is represented in each graph (mean ± SD). ns $P > 0.05$, *$P < 0.05$, **$P < 0.01$, ***$P < 0.001$, and ****$P < 0.0001$.

 © The Author(s)

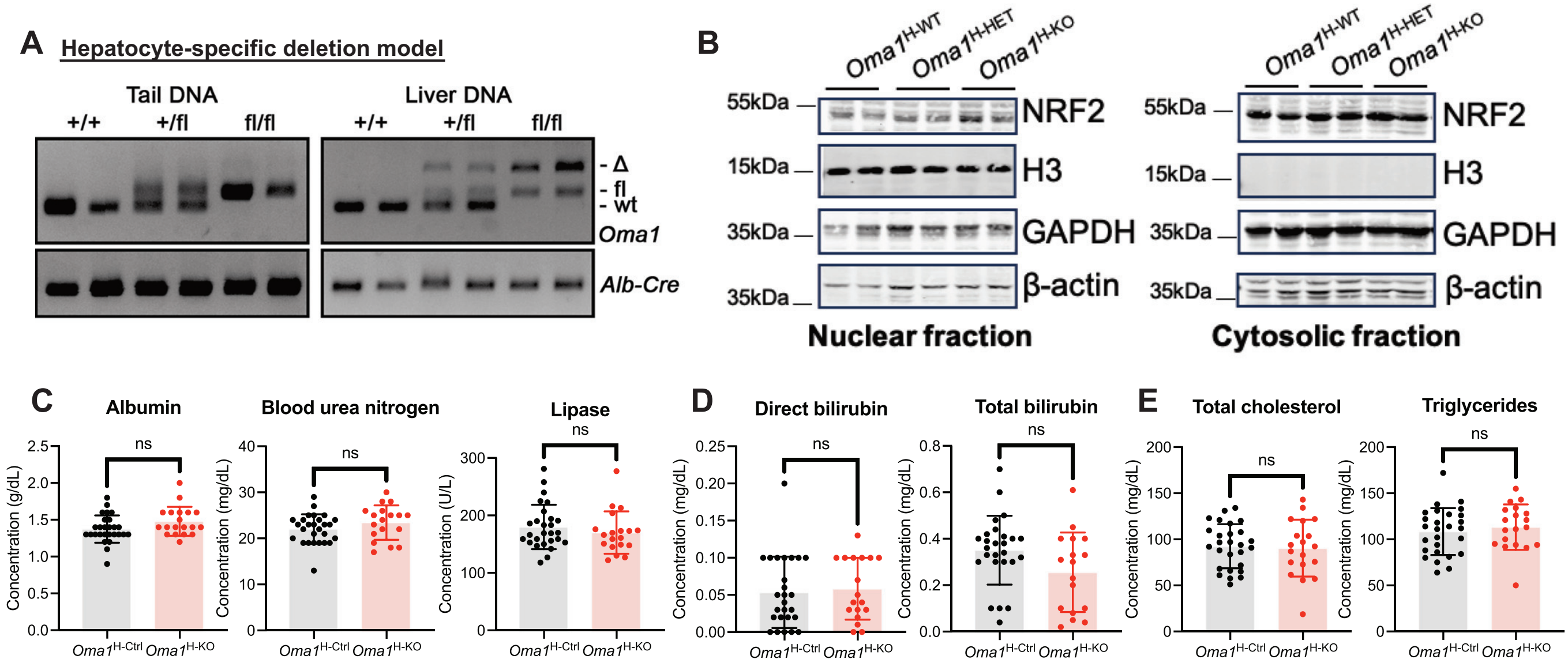

Figure EV8. Hepatocyte-restricted ablation of Oma1.

(**A**) *Oma1* genotype in tail and liver samples from 10 weeks-old male hepatocyte-specific *Oma1*-expressing (*Oma1*$^{\text{H-WT}}$ $n = 6$ and *Oma1*$^{\text{H-HET}}$ $n = 5$) or -deleted animals (*Oma1*$^{\text{H-KO}}$ $n = 6$). (**B**) Representative Western Blot from 10-week-old male hepatocyte-specific *Oma1*-expressing (*Oma1*$^{\text{H-WT}}$ $n = 10$) or -deleted animals (*Oma1*$^{\text{H-KO}}$ $n = 10$) nuclear and cytosolic liver fractions. (**C**) Plasma albumin, blood urea nitrogen, and lipase values from 10-week-old male hepatocyte-specific *Oma1*-expressing (*Oma1*$^{\text{H-WT}}$ $n = 15$ and *Oma1*$^{\text{H-HET}}$ $n = 12$) or -deleted animals (*Oma1*$^{\text{H-KO}}$ $n = 19$) (mean ± SD). "Albumin" values did not pass normality, while "BUN" and "Lipase" data passed normality. (**D**) Plasma direct and total bilirubin values from 10-week-old male hepatocyte-specific *Oma1*-expressing (*Oma1*$^{\text{H-WT}}$ $n = 15$ and *Oma1*$^{\text{H-HET}}$ $n = 12$) or -deleted animals (*Oma1*$^{\text{H-KO}}$ $n = 19$) (mean ± SD). Values did not pass normality. (**E**) Plasma total cholesterol and triglycerides values from 10-week-old male hepatocyte-specific *Oma1*-expressing (*Oma1*$^{\text{H-WT}}$ $n = 15$ and *Oma1*$^{\text{H-HET}}$ $n = 12$) or -deleted animals (*Oma1*$^{\text{H-KO}}$ $n = 19$) (mean ± SD). Values passed normality. *P* value from unpaired two-tailed Student's *t* test (when data passed normality) is represented in each graph. ns $P > 0.05$, $^{*}P < 0.05$, $^{**}P < 0.01$, $^{***}P < 0.001$, and $^{****}P < 0.0001$.

© The Author(s)

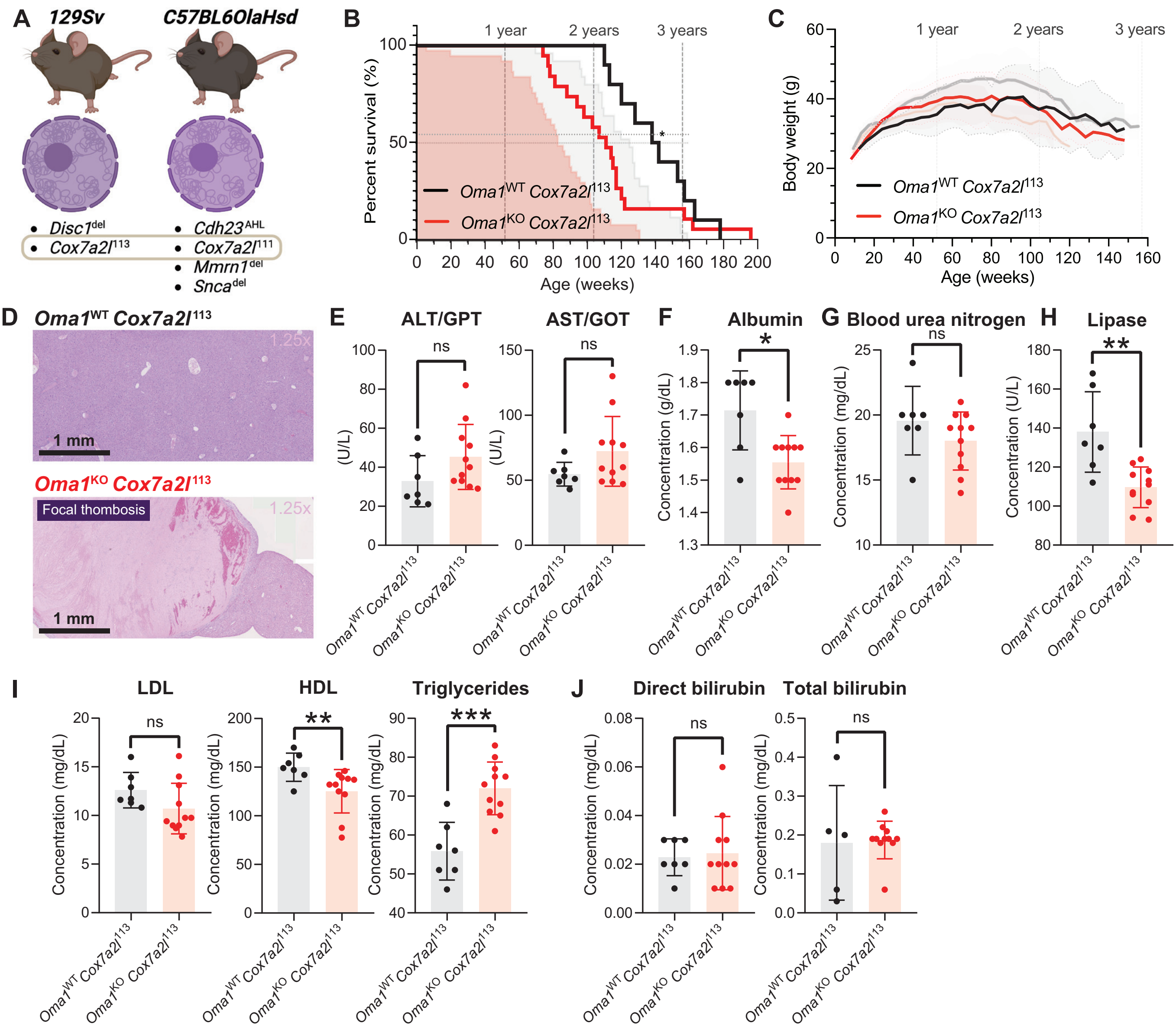

Figure EV9. Genetic background impact on OMA1-dependent CLD phenotype.

(**A**) Reported genetic background divergence between 129 Sv and C57BL6OlaHsd mice, including alterations in *Disc1*, *Cox7a2l*, *Cdh23*, *Mmrn1*, and *Snca*. (**B**) Survival analysis ($P = 0.0151$) of male $Oma1^{WT}$ $Cox7a2l^{113}$ ($n = 10$) and $Oma1^{KO}$ $Cox7a2l^{113}$ ($n = 19$) mice in an inbred C57BL6/JOlaHsd background. *P* value from Gehan–Breslow–Wilcoxon test is represented in the graph. (**C**) Body weight evolution of male $Oma1^{WT}$ $Cox7a2l^{113}$ ($n = 10$) and $Oma1^{KO}$ $Cox7a2l^{113}$ ($n = 19$) mice in an inbred C57BL6/JOlaHsd background. *P* value from a Mixed-effects model testing the "OMA1 expression" fixed effect is represented in the graph. (**D**). H&E histological analysis from a male $Oma1^{KO}$ mouse autopsy presenting focal liver thrombosis at 78 weeks of age, compared to a representative 86-week-old $Oma1^{WT}$ liver. (**E**) Plasma ALT-GPT, AST-GOT from 15-week-old male $Oma1^{WT}$ $Cox7a2l^{113}$ ($n = 7$) and $Oma1^{KO}$ $Cox7a2l^{113}$ ($n = 11$) mice fasted for 6 h. "ALT-GPT" and "AST-GOT" values passed normality (mean ± SD). (**F**) Plasma albumin ($P = 0.0108$) from 15-week-old male $Oma1^{WT}$ $Cox7a2l^{113}$ ($n = 7$) and $Oma1^{KO}$ $Cox7a2l^{113}$ ($n = 11$) mice fasted for 6 h. "Albumin" values did not pass normality (mean ± SD). (**G**) Plasma BUN from 15-week-old male $Oma1^{WT}$ $Cox7a2l^{113}$ ($n = 7$) and $Oma1^{KO}$ $Cox7a2l^{113}$ ($n = 11$) mice fasted for 6 h. "BUN" values passed normality (mean ± SD). (**H**) Plasma Lipase activity ($P = 0.0013$) from 15-week-old male $Oma1^{WT}$ $Cox7a2l^{113}$ ($n = 7$) and $Oma1^{KO}$ $Cox7a2l^{113}$ ($n = 11$) mice fasted for 6 h (mean ± SD). (**I**) Plasma LDL, HDL ($P = 0.0052$), and TAGs ($P = 0.0002$) in 15-week-old $Oma1^{WT}$ $Cox7a2l^{113}$ ($n = 7$) and $Oma1^{KO}$ $Cox7a2l^{113}$ ($n = 11$) mice fasted for 6 h. "LDL" and "TAGs" values passed normality, while "HDL" and "Total cholesterol" did not (mean ± SD). (**J**) Plasma direct bilirubin and total bilirubin from 15-week-old male $Oma1^{WT}$ $Cox7a2l^{113}$ ($n = 7$) and $Oma1^{KO}$ $Cox7a2l^{113}$ ($n = 11$) mice fasted for 6 h. "Direct bilirubin" and "Total bilirubin" values did not pass normality. *P* value from unpaired two-tailed Student's *t* test (when data passed normality) or Mann–Whitney test (when data did not pass normality) is represented in each graph (mean ± SD). ns $P > 0.05$, $^{*}P < 0.05$, $^{**}P < 0.01$, $^{***}P < 0.001$, and $^{****}P < 0.0001$.

 © The Author(s)
